# Supplementary material for: Study protocol for a randomised controlled trial of a virtual antenatal intervention for improved diet and iron intake in Kapilvastu district, Nepal: VALID
Source: BMJ Open. 2023 Feb 16;13(2):e064709. doi: 10.1136/bmjopen-2022-064709 (PMC9936277; doi:10.1136/bmjopen-2022-064709)
Supplement: Supplementary data [file bmjopen-2022-064709supp001.pdf]

Information sheet for VALID Baseline survey with Pregnant Women

Introduction

Namaste! My name is ____________________. I have come from HERD International located in Thapathali, Kathmandu. HERD International is a national level research organization. This organization has been conducting various programmes and research upon health, environment and social development. Currently, HERD International, in partnership with University College London, is conducting a study that aims to reduce anaemia in pregnant women in Kapilbastu. I would like to invite you to be a part of this study.

Before you decide whether to participate, it is important for you to understand why this research is being done and what participation will involve. I will read what is written in this information sheet aloud to you. You can ask me if there is anything that you do not understand or if you want more information. You will be given a copy of this information sheet. Take your time to decide whether or not you want to take part in the study or not. Thank you for reading this/listening to me.

Details of the Study

Anaemia is a condition when there is decreased haemoglobin in blood, it is associated with illness and complications during pregnancy and childbirth. HERD International, in partnership with University College London, is conducting a study with an aim to reduce anaemia in pregnant women. This study is aiming to improve anemia status among pregnant women in the community through virtual counselling session on nutrition, diet and antenatal care. Therefore, we would like to invite you to participate in this baseline survey we are conducting to understand your eligibility for the follow up study, socio demographics, dietary habits and antenatal care seeking behavior among pregnant women in the study area.

Why this survey?

We are conducting this study in 54 clusters or study areas in Kapilvastu district. Iron deficiency anemia is the main reason for anemia among pregnant women. Pregnant women who are anaemic are much more likely to die during childbirth than those women who are not and their infants are more likely to be born small for gestational age. During the baseline survey we will visit homes of all pregnant women in the study area to collect data, we expect to interview at least 1000 pregnant women, out of these women we hope to enroll at least 300 who are eligible (≤28 weeks' gestation) into the follow up study.

Why am I being invited to participate in this survey?

You can take part in this study if you are a pregnant woman or girl aged 13 to 49 years, live in the study area and are able to respond to the survey questions. If you are interested to take part in the study, we will give you this information sheet to read or have others read to you. Once you have listened-to (or read) and understood the information sheet we will ask you to give us sign the consent form agreeing to participate in the survey. If you

decide to participate, we will ask personal questions about you and your family (for eg: household head name, family size, ethnicity, religion, income and education) as well as your dietary habits, health problems and utilization of health care.

Will my taking part in this project be kept confidential?

All the information that we collect about you during the course of the research will be kept strictly confidential. Your information will be recorded using tablets by the researchers are involved in finding you to interview you. Only they will have access to your name and address, all other researchers who look at the information you share with us will not be able to identify you as your name and address will be removed.

Ethical approval

This study has been approved by the Nepal Health Research Council Approval ID number 570/2021.

Agreeing to take part

Your participation is voluntary. If you don't want to take part, you can refuse without giving a reason. If you decide to take part in the study, you will be given this information sheet to keep and be asked to sign or thumb print the consent. If you agree to participate and then change your mind at any time, please tell us and we will stop the interview at any point.

Are there any risks if you participate?

We do not think that any harm will come to you, but it is possible that you might find sharing information about your pregnancy uncomfortable or upsetting. You don't have to continue to take part if you don't feel like it.

Are there any benefits if you participate?

There is no direct benefit of participating in this study. However, the findings from this study will generate evidence to help develop health and nutrition programs and policies for pregnant women in future.

Contact for further information

You are encouraged to ask any questions you wish, before, during or after getting involved. If you require any further information or have any concerns while taking part in the study you can contact:

HERD International, Prasuti Griha Marg, Thapathali, Kathmandu. Tel 01-4238045

HERD International District Office, Taulihawa, Kapilbastu. Tel: number: 076-590090

Thank you for listening to / reading this information sheet and for considering whether to take part in this research study.

Virtual Antenatal Intervention for improved Diet and Iron intake (VALID) Trial

Participant Information Sheet for Pregnant Women

Introduction

Namaste! My name is ____________________. I have come from HERD International located in Thapathali, Kathmandu. HERD International is a national level research organization. This organization has been conducting various programmes and research upon health, environment and social development. Currently, HERD International, in partnership with University College London, is conducting a study that aims to reduce anaemia in pregnant women in Kapilbastu. I would like to invite you to be a part of this study.

Before you decide whether to participate, it is important for you to understand why this research is being done and what participation will involve. I will read what is written in this information sheet aloud to you. You can ask me if there is anything that you do not understand or if you want more information. You will be given a copy of this information sheet. Take your time to decide whether or not you want to take part in the study or not. Thank you for reading this/listening to me.

Details of the study

HERD International, in partnership with University College London, is conducting a study with an aim to reduce anaemia in pregnant women. The Medical Research Council (UK) is funding this research.

Anaemia is a condition when there is decreased haemoglobin in blood, and this is caused by various factors. In Nepal, lack of iron is the most common cause of anaemia in pregnancy. It is important to reduce anaemia in pregnancy because low iron levels are associated with illness and complications during pregnancy and childbirth. Pregnant women who are anaemic are much more likely to die during childbirth than those women who are not and their infants are more likely to be born small for gestational age.

In Nepal, Kapilbastu is one of the districts where anaemia is highly prevalent. Hence, we have chosen 54 clusters (103 old-wards) within 9 pallikas of Kapilbastu for this study. The government of Nepal recommends minimum 4 ANC visits during pregnancy and consumption of 180 tablets of iron and folic acid starting from 20 week's gestation. However, compliance to these recommendations is low, increasing the risk of anemia during pregnancy. The situation has become worse since March 2020 with ongoing COVID-19 imposed travel restrictions and lockdowns, which have made health care less accessible. The health and nutrition status of pregnant women has deteriorated as women are afraid to visit health facilities to seek care.

Objective:

The VALID trial is designed to assess if providing antenatal virtual counselling on a tablet increases compliance to intake of the required dosage of IFA tablets, and improves dietary diversity and dietary practices, compared with women who have access to routine antenatal care (ANC) only (control).

This trial is being implemented in 9 pallikas of Kapilvastu district among 13 to 49 years old married pregnant women. Around 300 pregnant women will be enrolled and allocated to either of the 2 trial arms.

In the intervention arm, in addition to routine pregnancy care, 150 pregnant women will be provided with electronic tablets (like large Smart Phones) which are fitted with sim cards to receive virtual counselling sessions. A female member of HERD staff who is qualified as an ANM (a “nutrition assistant”) will visit you in your home to lend you a tablet to provide virtual counselling session. After this she will provide you with two virtual counselling sessions: one between 12 to 28 week’s gestation and another two weeks after the first session. At each session she will discuss with you and your family members about your diet and health in pregnancy and give you advice based on what you are already eating / doing. She will encourage you to discuss and develop an action plan to improve your diet, consume iron tablets and antenatal care to minimize the risk of anaemia during pregnancy.

In the control arm, 150 pregnant women will be encouraged to go for routine pregnancy related services provided by the government of Nepal that includes antenatal care and iron and folic acid tablets.

Who are we inviting to participate?

You can take part in this research if you are a pregnant woman or girl aged 13 to 49 years, less than 28 weeks of gestation, planning to live in ,and/or seek services from the health facilities in, the study area and are able to respond to the survey questions. Pregnant women will be enrolled with the help of the health workers or female community health volunteers in these communities. If you are interested to take part in the trial, we will give you this trial information sheet to read or have others read to you. Once you have listened-to (or read) and understood the information sheet we will ask you to give us sign the consent form agreeing to participating in the trial. The risk or danger of participating in the trial is minimal. Participation in this trial is voluntary. If for any reason you want to withdraw your consent you can do so at any time.

What will happen if you agree to take part in this study?

If you decide to take part and give your consent (by signing or thumb-printing a consent form), first I will give you a unique identification number. On a tablet, I will record some personal details about you such as your age, education, household details, obstetric history, eating habits, iron supplements, physical activity and antenatal care. After responding to these questions, you will be assigned randomly to one of the two arms of the trial (intervention or control). It will take around 90 minutes to answer the questions and to randomly assign you to the trial arm.

Regardless of which arm you are assigned to, I will visit you again in about one month to enquire about your eating habits, iron and folic acid consumptions, antenatal care during pregnancy. This will take additional 90 minutes or so.

But if you are assigned to the intervention arm (virtual counselling) we will provide you a tablet with sim card to use during the intervention. You will receive two virtual counselling sessions 2 to 3 weeks apart and each session will last around 90 to 120 minutes. During these sessions we will discuss with you and your family members the importance of eating iron rich food during pregnancy, identify problems or barriers to eating iron rich food, identify solutions and develop action plans to implement them.

Therefore, if you are assigned to the intervention group, overall, you will need to provide around 5 hours' time, whereas if you fall in the control group (routine care) group you will need to provide 3 hours' minutes to the trial.

At the end of data collection, we will transfer 200 rupees' worth of mobile top up to your cell phone number or to a number of a person of your choice if you do not have your own cell phone.

Are there any risks if you participate?

We do not think that any harm will come to you, but it is possible that you might find sharing information about your pregnancy uncomfortable or upsetting. You don't have to continue to take part if you don't feel like it. If you would like to talk to someone about the feelings generated by the questions, please contact a member of HERD staff.

Are there any benefits if you participate?

There is no direct benefit of participating in this study, however you might enjoy interacting with the counsellor on the tablet. If found effective and feasible, the results of this trial will provide evidence that counselling can be provided virtually to improve health and nutrition of pregnant women from excluded communities in Kapilbastu and rest of Nepal.

Will my taking part in this project be kept confidential?

All the information that we collect about you during the course of the research will be kept strictly confidential. Only researchers directly associated with this project, who are involved in finding you to interview you, will have access to your name and address. All other researchers who look at the information you share with us will not be able to identify you as your name and address will be removed. You will be allocated a unique number, which will be used as a code to identify you instead of your name. You will not be able to be identified in any ensuing reports or publications.

If you consent to take part in this study, the records obtained while you are in this study (age, ethnicity, religion, education and so on) will remain strictly confidential at all times. The information will be held securely on either paper or electronically at HERD International and in University College London in the UK under the provisions the local Data Protection laws. Your name will not be passed to anyone else outside the research team who is not involved in the trial. Your records will be available to people authorized to work on the trial and those responsible for ensuring that the study is carried out correctly. By signing the consent form you agree to this access for the

current study. Further research might involve other researchers using the information you give us, but without your name attached to it. Alternatively, we or other researchers might seek to find you in the future to undertake further research with you.

If you withdraw consent from further study, unless you object, your data and samples will remain on file and will be included in the final study analysis.

Ethical approval

This study has been approved by the Nepal Health Research Council Approval ID number 570/2021.

Agreeing to take part

Your participation is voluntary. If you don't want to take part, you can refuse without giving a reason. If you decide to take part in the study, you will be given this information sheet to keep and be asked to sign or thumb print the consent. If you agree to participate and then change your mind at any time, please tell us and we will stop visiting you or telephoning you. We will take a photo of the consent form with your signature which will be filed in your records. You can have more time to think this over if you are at all unsure.

Data safety procedure

All participants will need to provide information two times during the trial, once at enrolment ≤ 28 weeks' gestation and another at 16 to 33 weeks' gestation. Depending on the COVID-19 situation, the information will be collected either over the phone or in person. Data assistants will assess eligibility to confirm pregnancy and gestational age, and take written consent before collecting data. The collected data will be kept confidential, stored in protected servers that can be assessed by authorised personnel only.

Expected outcome of the Trial"

In this study, the virtual nutritional counselling given to pregnant women through a tablet will help us to understand what kinds of improvements can be made in the regular intake of iron and folic acid, the consumption of iron-rich foods, and antenatal check-ups during pregnancy. As nutritional assistants will encourage antenatal check-ups and institutional delivery during the virtual counselling sessions, we hope that antenatal check-ups and institutional delivery will increase.

Use of the trial results:

The evidence generated by the trial can guide government and other stakeholders in development of plans and programs for maternal nutrition and to reduce the burden of anaemia during pregnancy.

Contact for further information

You are encouraged to ask any questions you wish, before, during or after getting involved. If you have any questions about the study, please speak to the HERD International researchers who visit you, who will be able to provide you with up to date information about the trial. If you require any further information or have any concerns while taking part in the study you can contact:

Trial Manager, HERD International, Prasuti Griha Marg, Thapathali, Kathmandu. Tel 01-4238045

Or

Dr Naomi Saville, Senior Research Associate, University College London Institute for Global Health and Technical advisor to HERD, Kathmandu Nepal. Tel: 01-4238045

HERD International District Office, Taulihawa, Kapilbastu. Tel: number: 076-590090

Thank you for listening to / reading this information sheet and for considering whether to take part in this research study.

Patient Identification Number for this trial:

CONSENT FORM

Title of Study: Virtual Antenatal Intervention for improved Diet and Iron intake (VALID) Trial

Please complete this form after you have read the Information Sheet and/or listened to an explanation about the research.

Name and Contact Details of the Researcher(s):

DR SUSHIL BARAL 9851068940 and DR NAOMI SAVILLE 9851017232

This study has been approved by the Nepal Health Research Council Approval ID number 570/2021.

Thank you for considering taking part in this research. The researcher who has come to your home or who you meet with over the telephone must explain the project to you before you agree to take part. If you have any questions arising from the Information Sheet or explanation already given to you, please ask the researcher before you decide whether to join the study. You will be given a copy of this Consent Form to keep and refer to at any time.

I confirm that I understand that by ticking/initialling each box below I am consenting to this element of the study. I understand that it will be assumed that unticked boxes means that I DO NOT consent to that part of the study. I understand that by not giving consent for any one element that I may be deemed ineligible for the study.

In case of the married girl of age 14 to 19 running years (13 to 18 completed years), take the consent from at least one of her guardians as well. This means filling the tick box columns with the participant **and** ***her guardian and getting the signature or thumb print of both of them.***

	Pregnant woman's Tick Box	Guardian's Tick box
I confirm that I have read the Information Sheet for the above study (or it has been read to me in an appropriate language) and I understood its content.		

I have had an opportunity to consider the information and what will be expected of me and to ask questions which have been answered to my satisfaction.		
I understand that my participation in this research is completely voluntary. I understand that I am free to withdraw from the research at any time without any repercussions. I understand that I can halt an interview at any point if I feel uncomfortable.		
I understand the potential risks of participating and the support that will be available to me should I become distressed during the course of the research.		
I understand that I will not benefit financially from this study or from any possible outcome it may result in in the future.		
I understand that my personal information *(including ethnicity, age, socio-economic and educational status, past medical and obstetric history)* will be used when analysing the data, but not my name.		
I understand that all personal information will remain confidential and that all efforts will be made to ensure I cannot be identified by anyone, except those who are involved in meeting me to ask questions.		
I understand that my data gathered in this study will be securely stored anonymously (without my name and address) and that it will not be possible to identify me in any publications.		
I understand that the data will not be made available to any commercial organisations but are solely the responsibility of the researcher(s) undertaking this study.		
I agree that my anonymised research data (that has my name and address removed) may be used by others for future research. I understand that no one will be able to identify you me if/when this these data are shared.		
I would be happy for the data I provide, including my name and address, to be archived in the UCL Data Safe Haven to be kept there, and other secure locations in Nepal and UK, in case researchers need to find me again in the future. I understand that these personally identifiable data will not be available to anyone except authorised researchers who need my name and address to find me again in the future.		

I understand that when I consent to have my photos taken while engaging in the research activities during the research period, that these could be published on HERD international's or UCL's website or other publications.		
I would be happy to be contacted in future by HERD International, UCL and/or other researchers who would like to invite me to participate in follow-up studies to this project (for example if someone came to measure me or my child after this study ended) **.**		
I have informed the researcher of any other research in which I am currently involved or have been involved in during the past 12 months.		
**I consent to participate in this study**		

Phone no for future contact:

Signing this document means that you voluntarily agree to participate in this research after understanding the information provided to you.

(In case the married participant's age is between 14 and 19 running years (13 -18 completed years)

______________________	______________	_________________
Name of guardian	Date	Signature

OR Guardian's Finger Prints

Left	Right

Name of participant	Date	Signature
______________________	______________	_________________

OR Participant's

Finger Prints

Left	Right<br><br>Left

Researcher (Data Assistant)	Date	Signature
____________________	______________	_________________

Nepali version of the Participnt information sheet

परियोजना परिचय: गर्भावस्थामा आईरन-फोलिक एसिड को सेवनमा सुधारका लागि भर्चुअल परामर्श कार्यको अध्ययन (VALID)

काठमाण्डौ स्थित हर्ड ईन्टरनेशनल एक राष्ट्रिय स्तरको अनुसन्धान गर्ने संस्था हो। यस संस्थाले स्वास्थ्य, वातावरण, र सामाजिक क्षेत्रमा उल्लेख्य मात्रामा कार्यक्रम तथा अनुसन्धानहरु गर्दै आईरहेको छ। हर्ड ईन्टरनेशनलद्वारा गर्भवती महिलाहरुको आईरन- फोलिक एसिड सेवनको अवस्थामा सुधार गर्नका लागि लुम्बिनी प्रदेशको कपिलवस्तु जिल्लामा “गर्भावस्थामा भर्चुअल परामर्श कार्यको अध्ययन” नामक अनुसन्धान गर्न लागेको ब्यहोरा अवगत गराउन चाहन्छौ। यस अनुसन्धान सञ्चालनका लागि यस संस्थाले नेपाल स्वास्थ्य अनुसन्धान परिषद् बाट स्विकृति प्राप्त गरिसकेको छ र स्वास्थ्य सेवा विभाग अन्तर्गत परिवार कल्याण महाशाखालाई अवगत गराएको छ। यस अध्ययन शुरू गर्नु अघि हामी प्रदेश तथा स्थानिय सरकारसंग समन्वय गरी आवश्यक सहयोग पनि लिनेछौं ।

पृष्ठभूमि

रक्तअल्पता भनेको रगतमा रातो कोशिकाको मात्रामा सामान्यतयाभन्दा कम हुनु वा प्रत्येक रातो कोशिकामा सामान्यतयाभन्दा कम हेमोग्लोबिन हुनु हो। आईरनको कमी नै रक्तअल्पता हुनुको सामान्य कारण हो। गर्भावस्थामा २० प्रतिशत मातृ मृत्यु सिधै रक्तअल्पतासँग सम्बन्धित छ । गम्भिर रक्तअल्पताका कारण विश्वमा हुने आधाभन्दा बढी मातृ मृत्यु दक्षिण एसियामा हुने गर्दछ। आईरनको कमीले गर्भावस्थामा जटिलता र अस्वस्थता हुने भएकाले गर्भवती अवस्थामा हुने रक्तअल्पतालाई घटाउन निकै जरुरी छ। बच्चा जन्माउने क्रममा रक्तअल्पता नभएका गर्भवती महिला भन्दा रक्तअल्पता भएका गर्भवती महिलाको सम्भवत मृत्यु दर बढी देखिन्छ र उनीहरुबाट कम तौल भएका शिशु जन्मने गर्दछन्।

नेपालको सन्दर्भमा अन्य क्षेत्रमा भन्दा गर्भवती महिलामा हुने रक्तअल्पता तराईमा (३६.४%) बढी देखिन्छ। सबै प्रदेशहरु मध्ये, उच्च रक्तअल्पता हुनेमा लुम्बिनी प्रदेश (४३.५%) दोश्रो स्थानमा पर्दछ। नेपालमा नियमित गर्भ जाँच गरिने (न्युनतम ४ पटक) र गर्भ रहेको २० हप्ता देखि नियमित आइरन तथा फोलिक एसिड (१८० चक्की) वितरण गरिने सरकारी प्रोटोकोल भएता पनि सरकारी प्रोटोकोल अनुसार नियमित गर्भ जाँच गर्ने महिलाहरुको संख्या न्युन रहेको र वितरण हुने आइरन फोलिक एसिड मा पहुच कम रहेको छ। जसले गर्दा गर्भावस्थामा हुने रक्तअल्पता अझै उच्च देखिन्छ। वर्तमान कोभिड-१९ को महामारी, यस समयमा स्वास्थ्य संस्था जानका लागि मानिसमा रहेको त्रास, महामारी नियत्रणका लागि पटक-पटक गरिएका विभिन्न स्वरुपका निषेधाज्ञाले गर्दा स्वास्थ्य सेवामा मानिसहरुको पहुच सिमित भएको छ र मातृ तथा शिशु पोषण स्थितीमा ह्रास आएको छ।

उद्देश्य

यस अध्ययनको उद्देश्य यस गर्भावस्थामा आइरन-फोलिक एसिडको सेवन नियमितता गराई आइरनको कमीले हुने रक्तअल्पता घटाई मातृ पोषणमा सुधार ल्याउनुको साथै उपलब्ध स्वास्थ्य सेवाको उपयोग र पहूँच बढाउनु हो।

विधिहरु

यो अध्ययन कपिलबस्तु जिल्लाको ९ वटा पालिकाहरुमा बस्ने १३ देखी ४९ वर्षका विवाहित गर्भवती महिलाहरुमा सञ्चालन गरिनेछ। हामी यस अध्ययनमा ३०० जना गर्भवती महिलालाई समावेश गराउने योजनामा छौँ, जसलाई दुईवटा समुहमा बाडिनेछ:

१. पहिलो १५० जना महिलाले यस अध्ययनको अवधिका लागी सिमकार्ड सहितको ट्याबलेट प्राप्त गर्नुहुनेछ र हाम्रो पोषण सहायकले ट्याबलेटको माध्यमबाट उँहाहरुलाई रक्तअल्पता, राम्रो खानपानको व्यवहार र गर्भ जाँच सम्बन्धि शिक्षा,परामर्श र सल्लाह प्रदान गर्नुका साथै उँहालाई खानपान सम्बन्धि सल्लाह दिनुहुनेछ। साथै गर्भवती महिलालाई नजिकको स्वास्थ्य संस्थामा गई गर्भवती जाँच गराउन प्रोत्साहन गर्नुहुनेछ। उँहाहरुले गर्भावस्थामा हुने रक्तअल्पताको समस्याका साथै खानपानमा सुधार ल्याएर, आइरन चक्कि सेवन गरेर र गर्भ जाँचका लागि गएर कसरी रक्तअल्पता घटाउन सकिन्छ भन्ने बारे छलफल गर्नुहुनेछ।

२. दोश्रो समूहको १५० जना महिलाले नेपाल सरकारको स्वास्थ्य कार्यक्रमद्वारा प्रदान भईरहेको स्वास्थ्य सेवा सहित नियमित गर्भ जाँच सेवा र आइरन चक्की प्राप्त गर्नेछन्।

हामी कसलाई सहभागी हुनका लागि आमन्त्रण गर्दैछौ?

हामी 12 देखि 24 हप्ता बीचको गर्भवतीहरु लाई मात्र यस अध्ययनमा सहभागी गराउने छौ । यस अध्ययनको लागि आवश्यक पर्ने गर्भवती महिलाहरु हामी स्वास्थ्य संस्था तथा स्वयमसेबीकाको सहायता मार्फत समाबेस गराउने छौ । तपाई यस अध्ययनमा सहभागी हुन योग्य भए नभएको एकीन तपाईको स्थानिय महिला स्वयं सेविका स्वास्थ्यकर्मीहरुले गर्नु हुनेछ । योग्य भएमा, उहाहरुले योग्य गर्भवती महिलाको सहमतिमा सम्पर्क फोन नम्बर

हर्डको तथ्याङ्क सहायकलाई दिनु हुनेछ। यदि तपाई यस ट्रायलमा सहभागी हुन इच्छुक हुनु हुन्छ भने, हामी तपाईलाई अध्ययन बारे जानकारी पत्र पढ्न दिनेछौ साथै यसमा दिएको जानकारी सबै बुझे पछि तपाईले मंजूरीनामा मा हस्ताक्षर गर्नु पर्ने हुन्छ। यस अध्ययनमा सहभागी हुदा हुन सक्ने जोखिम वा खतरा एकदम कम छ। यो अध्ययनमा तपाईको सहभागीता पूर्ण रुपमा स्वैच्छिक हो र यदि केही कारणबस बीचमा छोड्न चाहानुहुन्छ भने पनि तपाईले छोड्न पाउनु हुनेछ ।

यदि तपाई यस अध्ययनमा सहभागी हुनका लागि सहमत हुनुभएमा के हुन्छ ?

यदि तपाई यस ट्रायलमा सहभागी हुन मञ्जुरीनामा दिनुभएमा, हामी तपाईलाई केही व्यक्तिगत जस्तै परिवार, आम्दानी, प्रजनन स्वास्थ्य आदि र आइरन र् फोलिक ऐसिड सेवन बारे प्रश्नहरु सोध्ने छौ । यो प्रश्नहरु सोधीसके पछि हरेक सहभागी महिलाहरुलाई गोला प्रथा द्वारा (Random) अध्ययनको दुई समूह मध्य कुनै एक समूहमा समाबेश गर्नेछौ। यो सबै प्रक्रिया, प्रश्नहरु सोध्ने र समूहहरु बिभाजन गर्नको लागि लगभग ९० मिनेट समय लाग्ने छ ।

तपाई जुन समुहमा परेपनी, तपाई गर्भवती भएको ३२ देखि ३६ हप्ताको बीचमा हामी पुन: एकपटक प्रश्नहरु सोध्नको लागि भेट्नेछौ (फोन वा प्रत्यक्ष)। त्यो बेलामा हामी तपाईलाई गर्भवती जाँच, खानपान सम्बन्धी, आइरन र फोलिक ऐसिड सेवन, परिवारको सहयोग र अध्ययनमा सहभागी हुँदाको अनुभब बारे प्रश्नहरु सोध्ने छौ । यसको लागि तपाइले लगभग ९० मिनेट समयदिनु पर्ने हुन्छ ।

तर यदि तपाई भर्चुअल परामर्श (virtual counselling) समूहमा पर्नु भयो भने हामी तपाईलाई यस अध्ययन अवधिभरको लागि सिमकार्ड सहितको ट्याबलेट दिनेछौ। त्यसपछि हामी तपाईलाई २ पटक भर्चुअल परामर्शको लागि त्यस ट्याबलेटमा फोन गर्नेछौ र हरेक पटक लगभक ९० देखि १२० मिनेट समय दिनु पर्ने हुन्छ। भर्चुअल परामर्श सत्रमा तपाई र तपाईको परिवारको अन्य सदस्य संग गर्भावास्थामा आइरन युक्त खाने कुराको महत्व र आइरन युक्त खाना खानमा भएका समस्या सम्बन्धि छलफल गर्नेका साथै आवश्यकता अनुसारको परामर्श दिनेछौं र छलफलको आधारमा समस्या समाधान गर्नको लागि कार्ययोजना बनाउने छौ ।

यो अध्ययनमा सहभागी हुदा यदि भर्चुअल परामर्श समूहमा पर्नु भयो भने २४० मिनेट समय दिनु पर्छ भने यदि नियमीत सेवा समूहमा पर्नु भयो भने १८० मिनेट समय दिनु पर्ने हुन्छ ।

हामीले हरेक पटक तपाईलाई प्रश्नहरु सोध्दा र भर्चुअल परामर्श दिन फोन गर्दा तपाईको मोबाईलमा १०० रुपैया बराबरको पैसा पठाउने छौ ।

तपाई सहभागी भएमा कुनै जोखिमहरु छन्?

यस अध्यनमा भाग लिदा तपाईलाई कुनैपनि हानि पुग्नेछैन तर तपाईले आफ्नो महिनावारी वा गर्भवती सम्बन्धि जानकारी हामीलाई दिन अप्ठ्यारो वा असहज महसुस गर्न सक्नुहुन्छ। यदि तपाईलाई सहभागी हुन मन नलागेमा बिचमा छाड्न पनि सक्नुहुनेछ। यदि तपाईलाई हामीले सोधेका प्रश्नहरुबाट कुनै भावनाहरु उत्पन्न भई कसैसँग कुरा गर्न मन लागेमा कृपया हर्ड ईन्टरनेशनलको कर्मचारीलाई सम्पर्क गर्नुहोस्।

फाईदाहरु

यस अध्ययनमा सहभागि भएमा तपाईलाई प्रत्यक्ष केही फाइदा हुने छैन तर तपाईकै समुदाय तथा नेपालको अन्य भागमा रहनु हुने स्वास्थ्य सेवा बाट बन्चित भएका गर्भवती महिलहरुलाई यो अध्ययनको तथ्यांकले भर्चुअल बिधीबाट सेवा पुर्याउन सकीने रैछ भन्ने आधारहरु हुने छन् र उचित स्वास्थ्य सेवा, स्याहार, पोषणयुक्त खाना सम्बन्धि जानकारी हरु पाउने सक्ने छन ।

तपाईको जानकारी गोप्य राखिनेछ

हर्डको अन्तर्वार्ता लिने व्यक्ति वा स्थानिय महिला स्वास्थ्य स्वयंसेविकालाई दिनुभएका सम्पूर्ण जानकारी गोप्य राखिनेछन्, अर्थात तपाईले उहाँहरुलाई दिनुभएको जानकारी उहाँहरुले अरुलाई भन्ने अनुमति पाउनु भएको छैन।

हाम्रो रेकर्डमा तपाईलाई पहिचान गर्नका लागि युनिक नम्बरको (मेल नखाने परिचयात्मक संख्या) प्रयोग गरिनेछ। फलो अप गर्नुपर्ने सहभागी सूची तयार गर्ने व्यक्ति बाहेक सबैका लागि तपाईको पहिचान गोप्य राख्नका लागि नामको सट्टामा यो नम्बर प्रयोग गरिन्छ। तपाईले दिनुभएका जानकारीहरु पासवर्डले सुरक्षित कम्प्यूटरमा सुरक्षित राखिन्छन् र आधिकार प्राप्त सीमित व्यक्तिलाई मात्र त्यो हेर्न अनुमति दिईनेछ। तपाईले यस मञ्जुरिनामा मा हस्ताक्षर गरेपश्चात, तपाईले मञ्जुरीनामा रद्द नगरेसम्म तपाईको जानकारीहरु यस अध्ययनमा समावेश हुनेछ।

नैतिक स्विकृती

यस अध्ययनलाई नेपाल स्वास्थ्य अनुसन्धान परिषद् (परियोजना आई.डि.नम्बर:............) र यू.सी.एल अनुसन्धान तथा नैतिक समिति (परियोजना आई.डि नम्बर: ....) र लण्डन स्कुल अफ हाईजिन तथा ट्रपिकल मेडिसिन नैतिक समिती (आई.डी. नम्बर ....) द्वारा स्विकृत गरिएको छ ।

सहभागीताका लागि सहमती

तपाईको सहभागीता नितान्त स्वेच्छिक हुन्छ। यदि तपाईलाई सहभागी हुन मन छैन भने तपाईले कुनै पनि समस्या नमानि अस्वीकार गर्न सक्नुहुन्छ। यदि तपाई सहभागी हुनुभयो र कुनै पनि समयमा तपाई यस अध्ययनबाट हट्न चहानुभएमा कृपया हामीलाई भनिदिनुहोला। हामी तपाईकोमा आउन छाड्नेछौ। यदि तपाईले सहभागी हुने निर्णय लिएमा तपाईलाई यो जानकारी पत्र राख्न दिई लिखित मञ्जुरीनामा दिन अनुरोध गरिनेछ।

यस अध्ययनमा भाग लिने वा नलिने भन्ने कुराको निर्णय तपाई आफैले गर्ने हो। तपाई भाग लिन पनि सक्नु हुन्छ, नलिन पनि सक्नु हुन्छ र लिएर बिचमा छोड्न पनि सक्नु हुन्छ। यदि बिचमा छोड्न चाहनु हुन्छ भने तपाईले छोडे पछि तपाईको नाम अध्ययन बाट हटाईनेछ। तपाईले भाग नलिदा वा बिचमा छोड्दा यस अध्ययनका कर्मचारीहरु संग तपाईको सम्बन्धमा केहि फरक पर्ने छैन, तपाईलाई कुनै जरिवाना लाग्दैन, र तपाईले स्वास्थ्य संस्था बाट पाउने अन्य सुविधाहरुमा कुनै कमि आउने छैन।

तथ्याङ्क संकलन गर्ने तरिका

अध्ययनको सबै सहभागीहरुसंग दुई पटक तथ्याङ्क संकलन गरिनेछ - पहिलो पल्ट गर्भवती भएको १४ – २५ हप्तामा र दोश्रो पटक ३२- ३६ हप्ताको गर्भावस्थामा । तथ्याङ्क संकलन कोभीड – १९ को संक्रमणको अबस्थालाई हेरेर अवस्था अनुसार फोनबाट वा प्रत्यक्ष भेटेर गरिनेछ। गर्भवती महिलालाई गर्भको उमेर यकीन भएपछि, अध्ययनमा तथ्याङ्क सहायकले गर्भवती महिलासंग अध्ययनमा समावेश हुनका लागि फोनबाट मन्जुरिनामा लिनुहुनेछ। यस अध्ययनका सबै तथ्यांकहरु गोप्य राखिनुका साथै हर्डको कार्यालयमा सुरक्षित ढङ्गले सर्भर र कम्पयुटरहरुमा स्टोर गरिनेछ जसमा आधिकारिक व्यक्तिको मात्र पहुँच हुनेछ। सहभागीहरुलाई यस अध्ययनमा समावेश गर्नु अगावै लिखित मञ्जुरिनामा लिईनेछ।

अनुसन्धानको नतिजाहरुको अपेक्षित परिणाम

यस अध्ययन अन्तर्गत गर्भवती महिलालाई ट्याबलेट मार्फत भर्चुअल तरिका प्रयोग गरेर गरिएको पोषण परामर्शले गर्भवती महिलालले गर्भावस्थामा चाहिने आइरन र् फोलिक ऐसिडको नियमित सेवन, आइरन युक्त खाने कुराहरु खान र गर्भ जाँचमा के कस्तो सुधार ल्याउन सकिन्छ भन्ने कुरा बुझ्न मद्दत गर्नेछ। पोषण सहायकले भर्चुअल परामर्शका समयमा गर्भ जाँच र संस्थागत सुत्केरी गराउनमा प्रोत्साहन गर्ने भएकाले गर्भ जाँच र संस्थागत सुत्केरी बढ्छ भन्ने आशा राखेका छौँ। यस अध्ययनको परिणामहरुमा आएको संयुक्त सुधारको प्रभावले रक्तअल्पता घटाउनुका साथै गर्भवती महिलाको स्वास्थ्यमा सुधार ल्याउनेछ।

अनुसन्धानको नतिजाहरुको उपयोगको योजना

यस अनुसन्धानका नतिजाहरु मातृ पोषण र गर्भावस्थामा हुने रक्तअल्पता सम्बन्धि कार्यक्रमहरुको भावी योजना बनाउदा प्रमाणमा आधारित निर्णय लिन सम्बन्धित सरोकारवालाहरुलाई सूचित गराउनका लागि प्रयोग हुनेछन्।

अन्य जानकारी

यस अध्ययनबारे अन्य व्यक्तिसँग छलफल गर्नुपरेमा वा हामीलाई कुनै प्रश्नहरु सोध्नुपरेमा निर्धक्कसाथ सोध्न सक्नुहुन्छ।

यदि तपाईका अन्य प्रश्नहरु वा जिज्ञासा भएमा, निम्न व्यक्ति वा संस्थालाई सम्पर्क गर्नुहोला: ट्रायल म्यानेजर, हर्ड इन्टरनेशनल, प्रसुतिगृह मार्ग, थापाथली, काठमाण्डौ. सम्पर्क नं ०१-४२३८०४५

वा

डा. नावमी सेभिल, सिनियर रिसर्च असोसिएट, युनिभर्सिटी कलेज लन्डन ईन्स्टिच्यूट फर ग्लोबल हेल्थ र प्राविधिक सल्लाहाकार; हर्ड, काठमाण्डौ, नेपाल। सम्पर्क नं ०१-४२३८०४५

वा

हर्ड ईन्टरनेशनल जिल्ला कार्यालय, तौलीहवा, कपिलबस्तु,सम्पर्क नं ०७६-५९००९०

Awadhi version of the Participnt information sheet

परियोजना परिचय: गर्भावस्थामे आईरन-फोलिक एसिड कै सेवनमे सुधार कै खातिर भर्चुअल परामर्श कार्य कै अध्ययन (VALID)

काडमाण्डौमे रहेवाला हर्ड ईन्टरनेशनल राष्ट्रीय स्तर कै अनुसन्धान करेवाला संस्था होय। यी संस्था स्वास्थ्य, वातावरण अउर सामाजिक क्षेत्रमे उल्लेख्य कार्यक्रम औ अनुसन्धान करत आवत है। हर्ड ईन्टरनेशनल गर्भवती महतारीमे आईरन-फोलिक एसिड सेवन कै अवस्थामे सुधार कै खातिर लुम्बिनी प्रदेश कै कपिलवस्तु जिलामे "गर्भावस्थामा भर्चुअल परामर्श कार्यको अध्ययन" नाव कै अनुसन्धान करेवाला जानकारी अनुरोध काराजात है। यी अनुसन्धान सञ्चालन कै खातिर यी संस्था नेपाल स्वास्थ्य अनुसन्धान परिषद्से अनुमति पायी चुकाहै अउर स्वास्थ्य सेवा विभाग अन्तर्गत कै परिवार कल्याण महाशाखाके अवगत कराय चुकागयहै। यी अध्ययन शुरु करे से पहिले हमरे प्रदेश अउर स्थानिय सरकारसे समन्वय कईकय आवश्यक सहयोग भी लेवाजाइ।

पृष्ठभूमि

रक्तअल्पता वा खून कै कमीके मतलब खून मै लाल कोशिका कै मात्रा सामान्य अवस्थासे कम होयक वा हरेक लाल कोशिकामे सामान्यसे कम हेमोग्लोबिन होयक अवस्था होय। आईरन कै कमी ही रक्तअल्पता होयक सामान्य अउर प्रमुख कारण होय। गर्भवती अवस्था मै महतारी कय मृत्यु कय कारण मध्ये २० प्रतिशत कारण रक्तअल्पतासे सिधै सम्बन्धित होतहय। गम्भिर रक्तअल्पता कै कारणसे विश्वमे होयवाला आधासे ज्यादा महतारी कै मृत्यु दक्षिण एसिया मै होतहय। आईरन कै वजहसे गर्भावस्थामे जटिलता अउर अस्वस्थता होयक वजहसे गर्भवती अवस्थामे होयवाला रक्तअल्पता घटायक बहुत ही जरुरी है। महतारी सौरी होत कै समयमे रक्तअल्पता नहोअल गर्भवती महतारी से ज्यादा रक्तअल्पता होअल गर्भवती महतारी कै मृत्यु होयक सम्भावना ज्यादा देखात है अउर वनसे कम तौल कै शिशु कै जन्म होला।

नेपाल कै सन्दर्भमे गर्भवती महतारीमे होयवाला रक्तअल्पता अन्य जगहसे तराईमे ज्यादा (३६.४%) देखातहय। सब प्रदेशमे से उच्च रक्तअल्पता होयवालामे लुम्बिनी प्रदेश दुसरा (४३.५%) स्थानमे परत है। नेपालमे नियमित गर्भजाच (कमसे कम ४ दाँयी) अउर पाउभारी होअल १३ हप्तासे नियमित आईरन अउर फोलिका एसिड (१८० गोली) वितरण करेवाला सरकारी नियम है लेकिन सरकारी नियम अनुसार नियमित गर्भजाच करेवाला संख्या बहुत कम है अउर वितरण होयवाला आईरन फोलिक एसिड गोलीमे पहुच भी बहुत कम है। जेकरे वजहसे गर्भावस्था मै होयवाला रक्तअल्पता बहुत ज्यादा देखातहय। वर्तमान कोभिड-१९ कै महामारी कै समय मै स्वास्थ्य संस्था जायक लिए मनईनमे रहल डर, महामारी नियन्त्रण कै खातिर बार बार किया गवल बिभिन्न स्वरुप कै निषेधाज्ञा कै वजहसे स्वास्थ्य सेवामे मनईन कै पहुच सिमित भयक वजहसे भी महतारी अउर शिशु कै पोषण स्थिति मै ह्रास आवल है।

उद्देश्य

यी अध्ययन कै उद्देश्य गर्भावस्था मै आईरन-फोलिक एसिड कै सेवन नियमित करवाइ कै आईरन कै कमी से होयवाला रक्तअल्पता घटाई कै गर्भवती महतारी कै पोषणमे सुधार करेक साथै उपलब्ध स्वास्थ्य सेवा कै उपयोग औ पहुच बढायक होय।

विधि

यी अध्ययन कपिलवस्तु जिला कै ९ ठु पालिकामे रहेवाला १३ से लईकय ४९ वरष तक कै शादिशुदा गर्भवती महतारीमे संचालन कराजाई। हमरे यी अध्ययन मै ३०० जने गर्भवती महतारी कै समावेश करेक योजना है, जे का दुई ठु समुह मै बाँटा जाइ:

३. पहिला समुहमे १५० जने महिला कै यी अध्ययन कै अवधिके लिए सिमकार्ड सहित कै ट्याबलेट (बडका स्क्रिनवाला मोबाईल) देवा जाइ अउर हम्मन कै पोषण सहायक उ ट्याबलेट कै माध्यमसे वन्नहनकै रक्तअल्पता, बढिया खानपान कै व्यवहार अउर गर्भ जाँचसे सम्बन्धित शिक्षा, परामर्श औ सल्लाह देवेकै साथै खानपान सम्बन्धि सल्लाह दिहै। साथै गर्भवती महतारी कै नजिक कै स्वास्थ्य संस्थामे जाई कै गर्भवती जाँच करेक लिय प्रोत्साहित करिहै। वन्हरे गर्भावस्थामे होयवाला रक्तअल्पताके समस्या कै साथै खानपानमे सुधार कईकै, आईरन गोली सेवन कईकै अउर गर्भ जाँच कईकै कैसे रक्तअल्पता घटाई सकाजातहै वकरे बारेमे छलफल करीहै।
४. दुसर समूह कै १५० जने महिला कै नेपाल सरकार कै स्वास्थ्य कार्यक्रमद्वारा प्रदान होअल स्वास्थ्य सेवा सहित नियमित गर्भ जाँच अउर आईरन गोली प्राप्त करिहै।

हमरे के का सहभागी होयक लिय आमन्त्रण कराजातहै?

हमरे १२ से २८ हफ्ता बीच कै गर्भवती महतारी लोगन कै मात्र यी अध्ययनमे सहभागी करावाजाइ । यी अध्ययन कै लिए आवश्यक परेवाला गर्भवती महतारी हमरे स्वास्थ्य संस्था अउर स्वयंसेविक कै सहयोगसे समावेस करावाजाइ । आप यी अध्ययन मै सहभागी होयक योग्य होअल नहोअल एकिन आप कै स्थानिय महिला स्वयंसेविका, स्वास्थ्यकर्मीके साथ मै आयवाला हर्ड कै तथ्याङ्क सहायक करिहै । आप योग्य होअल नहोअल एकिन करेक अउर अध्ययनमे सहभागी होयक नहोयक निर्णय लेक लिए हमरे आप कै यी ट्रायल कै बारेमे पत्र पढे कै देवाजाइ साथमे यह मै दिहल जानकारी सब समझेक बुझेक बादमे आप कै मंजुरीनामामे हस्ताक्षर करे कै पडी । यी अध्ययन मै सहभागी होयसे होयवाला जोखिम वा खतरा एकदम कम है । यी अध्ययनमे आप कै सहभागीता पूर्ण रुपसे आपके विचार उपर है अउर अगर कौनो कारणसे बीचमे छोडेक चाहाजात है तो छोडक पावाजाइ ।

अगर आप यी अध्ययनमे सहभागी होयक लिए सहमत होवाजाइ तो का होइ ?

अगर आप यी ट्रायलमे सहभागी होयक मञ्जुरीनामा दियाजाइ तो हमरे आप कै कुछ व्यक्तिगत जईसे परिवार, कमाई, प्रजनन स्वास्थ्य आदि अउर आईरन-फोलिक एसिड सेवन कै बारेमे प्रश्न पुछाजाइ । यी कुल प्रश्न पुछे कै बादमे हरेक सहभागी महिला लोगन कै गोला प्रथासे अध्ययन कै दुई समूह मे से कौनो एक समूह मै समावेश कराजाइ । यी कूल प्रक्रिया, प्रश्न पुछेवाला काम अउर समुह बिभाजन करेक लिए लगभग ९० मिनेट समय लागी । आप जौन कौनो समूहमे परेपर भी हमरे फिर एक पटक प्रश्न पुछेक लिए लगभग एक महिनामे भेट करेक लिए आवाजाई (फोन वा प्रत्यक्ष)। उ समयमे हमरे आप कै गर्भवती जाँच, खानपान कै बारेमे, आईरन अउर फोलिक एसिड, परिवार कै सहयोग अउर अध्ययनमे सहभागी होत कै अनुभवके बारेमे प्रश्न पुछाजाइ । यकरे लिए आप लगभग ९० मिनेट जतना समय दियक पडी ।

लेकिन अगर आप भर्चुअल परामर्श समूहमे पराजाइ तो हमरे आप कै यी अध्ययन अवधिभर कै लिए सिमकार्ड सहित कै ट्याबलेट देवाजाइ । वकरे बादमे हमरे आप कै दुई दाँयी भर्चुअल परामर्शके लिए उ ट्याबलेटमे फोन कराजाइ अउर हरेक दाँयी लगभग ९० से १२० मिनेट समय देकय पडी । भर्चुअल परामर्श सत्र मै आप अउर आप कै परिवार कै अन्य सदस्य संघरी गर्भावस्थामे आईरन युक्त खान कै महत्व अउर आईरन युक्त खाना खायम होअल समस्या सम्बन्धि छलफल करेक साथै आवश्यक्ता अनुसार कै परामर्श देवाजाइ अउर छलफलके आधार मै समस्या समाधान करेक खातिर कार्ययोजना बनावाजाइ ।

यी अध्ययनमे सहभागी होय पर अगर भर्चुअल परामर्श समूहमे परागय तो लगभग ४२० मिनेट समय देयक पडी अउर अगर नियमित सेवा समुहमे परा गय तो १८० मिनेट जतना समय देयक पडी ।

हमरे हरेक बार आपसे प्रश्न पुछतके अउर भर्चुअल परामर्श देत कै फोन कराजाइ तो आप कै मोबाईल मै १०० रुपैया बराबर पैसा पठावा जाइ ।

आपके सहभागी होये पर कौनो जोखिम है ?

यी अध्ययन मै भाग लिहेसे आप कै कौनो भी हानी पहुचेवाला नाहि है लेकिन आपन गर्भावस्था कै जानकारी हम्मन कै देयक परेशानी औ असहज महसुस होय सकत है । अगर आप कै सहभागी होयक मन नाहि रहि तो बिचमे छोड भी सकाजात है । अगर आप कै हम्मनके पुछल कौनो प्रश्नसे कौनो भावना पैदा होय कै केहुसे बात करेक मन लागेपर कृपया हर्ड कै कर्मचारीसे सम्पर्क कराजाइ ।

फाईदा

यी अध्ययन मै सहभागी होय पर आप कै प्रत्यक्ष कुछ फाइदा नाहि होयी लेकिन आप कै समुदाय अउर नेपाल कै अउर जगहीमे रहल स्वास्थ्य सेवासे बन्चित भवल गर्भवती महतारीयन कै यी अध्ययन कै तथ्याङ्कसे भर्चुअल तरिकासे सेवा पहुचायक सकाजात है कहेवाला आधार होयी अउर उचित स्वास्थ्य सेवा, स्याहार, पोषण रहल खाना कै बारेमे जानकारी पायसकत है ।

आप कै जानकारी गोप्य रख्खाजाइ

हर्डसे अन्तरवार्ता लेवाला ब्यक्ति औ स्थानिय महिला सामुदायिक स्वास्थ्य स्वयंसेविका कै देवल सब जानकारी गोप्य रख्खाजाइ, मतलब आप वन कै देवल जानकारी वन्नहरे औरो केहुसे कहेक अनुमति नाहि मिला है । हम्मन कै रेकर्डमे आप कै चिन्हेक लिये युनिक नम्बर (दुसरे से नमिलेवाला परिचयात्मक संख्या) कै प्रयोग कराजाइ । बादमे भेट (फलो अप) करेवाला सहभागी सूची तयार करेवाला व्यक्तिके सिवाय सबके लिये आप कै पहिचान गोप्य रख्खे कै लिये नाव कै औजि पर यी नम्बर प्रयोग कियाजाइ । आप कै देवल जानकारी पासवर्डसे सुरक्षित करल कम्प्यूटर मै सहेज कै रखिजाइ अउर अधिकार पावल कुछ सीमित व्यक्ति कै भर यी देखे कै अनुमति देवाजाइ । आप यी मञ्जुरीनामामे हस्ताक्षर करेक बादमे, आप यइ मञ्जुरीनामा रद्द नकरे तक आप कै जानकारी यी अध्ययन मै समावेश होयी ।

नैतिक स्विकृती

यी अध्ययन कै नेपाल स्वास्थ्य अनुसन्धान परिषद् (परियोजना आई.डि.नम्बर: ५७०।२०२१) से स्विकृत भवा है।

सहभागीता कै लिये सहमती

आप कै सहभागीता पुरापुर आप कै बिचार उपर रहि । अगर आप कै सहभागी होयक मन नाहि है तो कौनो भी दिक्कत परेसानी नमान कै अस्वीकार कै सका जात है। अगर आप यी अध्ययनमे सहभागी होवागै अउर कौनो भी समयमे आप यी अध्ययनसे हटेक चाहाजात है तो हम्मन से कहाँ जाइ । हमरे आपके यहा नाहि आवाजाइ । अगर आप सहभागी होयेक निर्णय लेवाजाइ तो यी जानकारी पत्र आप कै रख्खे देवाजाइ अउर लिखित मञ्जुरीनामा देयकै अनुरोध कराजाइ । यी अध्ययन मै भाग लियेक औ नलियेक निर्णय आप कै अपने करेक है । आप सहभागी होय सकाजात है, न भी होयसकाजात है अउर बिचमे भी छोड सकाजात है । अगर बिचमे छोडे कै चाँहा जात है तो आप कै छोडे कै बादमे आप कै नाव अध्ययनसे हटाइजाइ । आप कै सहभागी नहोयेसे अउर बिच मै छोडेसे यी अध्ययन कै कर्मचारीयनसे आप कै सम्बन्ध मै कुछ फरक परेवाला नाहि है, आप कै कौनो जरिवाना भी नाहि लागी अउर आप कै स्वास्थ्य संस्थासे पायेवाला और कौनो सुविधा मै कुछ भी कमी नाहि आयेवाला है ।

तथ्याङ्क संकलन करेवाला तरिका

अध्ययन कै सब सहभागीयन से दुई दाँयी तथ्याङ्क संकलन कराजाइ – पहिला दाँयी गर्भवती होअल १२ – २८ हफ्तामे अउर दुसरा दाँयी १६ – ३२ हफ्ताके गर्भावस्थामे । तथ्याङ्क संकलन कोभीड – १९ कै संक्रमण कै अवस्था देख कै फोनसे वा प्रत्यक्ष भेटसे कराजाइ । गर्भवती महतारीके गर्भ कै महिना पता चलेक बादमे, अध्ययनमे तथ्याङ्क सहायक गर्भवती महतारीसे अध्ययनमे सहभागी होयेक लिये फोनसे मञ्जुरीनामा लिहैं । यी अध्ययन

कै सब तथ्याङ्क गोप्य रख्खे कै साथै हर्ड कै कार्यालयमे सुरक्षित रुपसे सर्भर अउर कम्प्युटरमे रख्खाजाइ जेहमा आधिकारिक व्यक्ति कै भर पहुच रही। सहभागीयन कै यी अध्ययनमे सहभागी होयेसे पहिले ही लिखित मञ्जुरिनामा लिहाजाइ।

अनुसन्धान नतिजा कै अपेक्षित परिणाम

यी अध्ययनमे गर्भवती महतारी कै ट्याबलेटसे भर्चुअल तरिका प्रयोग कइ कै करल पोषण परामर्शसे गर्भवती महतारी कै गर्भावस्थामे चाहेवाला आईरन औ फोलिक एसिडके नियमित सेवन, आईरन होअल खाना खायेक अउर गर्भ जाँचमे कैसन सुधार कै सकाजात है कहेवाला बतिया बुझेक मद्त होइ। पोषण सहायकसे भर्चुअल परामर्शके समयमे गर्भ जाँच अउर संस्थागत सौरी करवायेक प्रोत्साहन करेक वजहसे गर्भ जाँच अउर संस्थागत सौरी बढी कहि कै आशा रख्खा गै है। यी अध्ययन कै परिणाम मै आवल संयुक्त सुधार कै प्रभावसे रक्तअल्पता घटावै कै साथै गर्भवती महतारी कै स्वास्थ्य मै सुधार लायी।

अनुसन्धान कै नतिजा कै उपयोग कै योजना

यी अनुसन्धानसे आवल नतिजा मातृ पोषण अउर गर्भावस्थामे होयेवाला रक्तअल्पता जैसन कार्यक्रम कै भावी योजना बनावत कै प्रमाणके आधारमे निर्णय लेकलिये सम्बन्धित सरोकारवालन कै सूचित करायेक खातिर प्रयोग कयिजाइ।

और जानकारी

यी अध्ययन बारेमे और व्यक्ति संघरी छलफल करेक वा हम्मन कै कौनो प्रश्न पुछेक है तो बिना हिचकिचाहट पुछसकाजात है।

अगर आप कै अउर प्रश्न वा जिज्ञासा है तौ, निचे दिहल व्यक्ति वा संस्था कै सम्पर्क कियाजाइ:

ट्रायल म्यानेजर, हर्ड इन्टरनेशनल, प्रसुतिगृह मार्ग, थापाथली, काठमाण्डौ. सम्पर्क नं ०१-४२३८०४५

वा

डा. नावमी सेभिल, सिनियर रिसर्च असोसिएट, युनिभर्सिटी कलेज लन्डन ईन्स्टिच्यूट फर ग्लोबल हेल्थ औ प्राविधिक सल्लाहाकार; हर्ड, काठमाण्डौ, नेपाल। सम्पर्क नं ०१-४२३८०४५

वा

हर्ड ईन्टरनेशनल जिल्ला कार्यालय, तौलीहवा, कपिलबस्तु, सम्पर्क नं ०७६-५९००९०
