# Supplementary material for: Study protocol for a randomised controlled trial of a virtual antenatal intervention for improved diet and iron intake in Kapilvastu district, Nepal: VALID
Source: BMJ Open. 2023 Feb 16;13(2):e064709. doi: 10.1136/bmjopen-2022-064709 (PMC9936277; doi:10.1136/bmjopen-2022-064709)
Supplement: Author's manuscript [file bmjopen-2022-064709.draft_revisions.pdf]

BMJ Open is committed to open peer review. As part of this commitment we make the peer review history of every article we publish publicly available.

When an article is published we post the peer reviewers' comments and the authors' responses online. We also post the versions of the paper that were used during peer review. These are the versions that the peer review comments apply to.

The versions of the paper that follow are the versions that were submitted during the peer review process. They are not the versions of record or the final published versions. They should not be cited or distributed as the published version of this manuscript.

BMJ Open is an open access journal and the full, final, typeset and author-corrected version of record of the manuscript is available on our site with no access controls, subscription charges or pay-per-view fees (http://bmjopen.bmj.com).

If you have any questions on BMJ Open's open peer review process please email info.bmjopen@bmj.com

BMJ Open

A study protocol for a randomised controlled trial of a Virtual Antenatal Intervention for improved Diet and Iron intake in Kapilbastu district, Nepal: VALID

Journal:	*BMJ Open*
Manuscript ID	bmjopen-2022-064709
Article Type:	Protocol
Date Submitted by the Author:	10-May-2022
Complete List of Authors:	Saville, Naomi; University College London, Institute for Global Health<br>Bhattarai, Sanju; HERD International<br>Harris-Fry, Helen; LSHTM<br>Giri, Santosh; HERD International<br>Manandhar, Shraddha; HERD International<br>Morrison, Joanna; University College London, Institute for Global Health<br>Copas, Andrew; University College London, Institute for Global Health<br>Thapaliya, Bibhu; HERD International<br>Arjyal, Abriti; HERD International<br>Haghparast-Bidgoli, Hassan; University College London, Institute for Global Health<br>Baral, Sushil; HERD International; HERD<br>Hillman, Sara; University College London, Institute for Women's Health
Keywords:	NUTRITION & DIETETICS, PUBLIC HEALTH, EPIDEMIOLOGY

SCHOLARONE™
Manuscripts

A study protocol for a randomised controlled trial of a Virtual Antenatal Intervention for improved Diet and Iron intake in Kapilbastu district, Nepal: VALID

Naomi M. Saville[1]*, Sanju Bhattarai[2], Helen Harris-Fry[3], Santosh Giri[2], Shraddha Manandhar[2], Joanna Morrison[1], Andrew Copas[1], Bibhu Thapaliya[2], Abriti Arjyal[2], Hassan Haghparast-Bidgoli[1], Sushil Baral[2,4], Sara Hillman[5]

Author affiliations:

1. Institute for Global Health, University College London (UCL), London, UK
2. HERD International, Thapathali, Kathmandu, Nepal
3. London School of Hygiene & Tropical Medicine (LSHTM), London, UK
4. Health Research and Social Development Forum (HERD), Kathmandu, Nepal
5. Institute for Women's Health, University College London (UCL), London, UK

* Corresponding author: Naomi Saville, email: n.saville@ucl.ac.uk

Trial registration

Trial Registry name: ISRCTN; Registration number: ISRCTN 17842200; Date of registration: 13 Jan 2022. URL: https://doi.org/10.1186/ISRCTN17842200

World Health Organization Trial Registration Data Set

ISRCTN collects all items from the World Health Organization Trial Registration Data Set.

Protocol version

Version number 1.0, 1 May 2022

Funding

This work was supported by UK Medical Research Council (MRC)/ Newton Fund, grant number MR/R020485/1. The funding body has no role in the study design, collection, analysis, or interpretation of data, in the writing of the manuscript, or the decision to submit the report for publication.

Trial sponsor

For peer review only

Trial sponsor is Health Research and Social Development Forum (HERD) Nepal, PO Box 24133, Prasuti Griha Marg, Thapathali, Kathmandu, Nepal.

The trial sponsor, HERD Nepal under the leadership of Sushil C. Baral, working with the trial coordinating centre, University College London Institute for Women's Health, takes responsibility for study design; collection, management, analysis, and interpretation of data; writing of the report; and the decision to submit the report for publication. Authority over these activities is shared between HERD and UCL.

Composition of the coordinating centre, Trial Steering Committee and Data Monitoring Committee

The Trial Management Group (TMG) comprises representatives from HERD International (SCB, SB, SM and SG), the PI / local PI, process evaluation adviser and trial statistician from UCL (SH, NS, JM, AC) and dietary specialist (HHF). The TMG meet weekly.

The Trial Steering Committee (TSC) comprises independent specialists on maternal nutrition, anaemia, haematology, conduct of trials, and/or maternal nutrition in Nepal from the UK, India, Nepal, and Canada. The TSC provides oversight and advice through its independent Chair in alignment with the TSC charter. The TSC met by teleconference prior to the start of enrolment to approve the protocol and will meet after data collection has completed. Between meetings any trial issues arising are dealt with by email.

The Data Monitoring Committee (DMC) comprises experts on global health trials, statistics, obstetric haematology, maternal nutrition, micronutrient status and research ethics in Nepal, with two members from Nepal and the remaining three from the US, UK, and India. While interim safety data cannot be reviewed by the DMC due to the short trial duration, the DMC will review and approve the Trial Analysis Plan.

Declaration of interests

The authors declare that they have no competing interests.

For peer review only

Abstract

Introduction: Despite evidence that iron and folic acid (IFA) supplements can improve anaemia in pregnant women, uptake in Nepal is suboptimal. We hypothesised that providing virtual antenatal counselling twice in mid-pregnancy, would increase compliance to IFA tablets during the COVID-19 pandemic compared with ANC alone.

Methods and Analysis: This non-blinded individually randomised controlled trial in the plains of Nepal has two study arms: 1) Control: routine antenatal care (ANC); 2) 'Virtual' antenatal counselling plus routine ANC. Pregnant women are eligible to enrol if they are married, aged 13-49 years, able to respond to questions, 12-28 weeks' gestation, and plan to reside in Nepal for the next 5 weeks. The intervention comprises two virtual antenatal counselling sessions facilitated by auxiliary nurse midwives at least 2 weeks apart in mid-pregnancy. Virtual antenatal counselling uses a dialogical problem-solving approach with pregnant women and their families. We randomised 150 pregnant women to each arm, stratifying by primi-/multi-gravida and IFA consumption at baseline, providing 80% power to detect a 15% absolute difference in primary outcome assuming 67% prevalence in control arm and 10% loss-to-follow-up. Outcomes are measured 49-70 days after enrolment, or up to delivery otherwise. Primary outcome: consumption of IFA on at least 80% of the previous 14 days. Secondary outcomes: dietary diversity, consumption of intervention-promoted foods, practicing ways to enhance bioavailability, and knowledge of iron-rich foods. Our mixed methods process evaluation explores acceptability, fidelity, feasibility, coverage (equity and reach), sustainability, and pathways to impact. We estimate costs and cost-effectiveness of the intervention from a provider perspective. Primary analysis is by intention-to-treat, using logistic regression.

Ethics and Dissemination: We obtained ethical approval from Nepal Health Research Council (**570/2021**) and UCL ethics committee (**14301/001**). We will disseminate findings in peer reviewed journal articles and by engaging policymakers in Nepal.

Trial registration number: ISRCTN 17842200

For peer review only

Strengths and limitations of this study

1. The virtual counselling intervention under test with pregnant women is novel and tailored to a pandemic situation where opportunities for face-to-face antenatal counselling may be limited.
2. The intervention design to engage pregnant women and their family members in a problem-solving interactive discussion is based upon formative research on the barriers to uptake of iron and folic acid supplements and antenatal care.
3. Carefully designed electronic data collection forms enable thorough follow-up of participants, drawing on their information at recruitment where needed to validate outcome data.
4. Necessary adaptation to the COVID-19 context led to delays in starting the trial, constraining the time frame within the funding period. This means that the time frame to enrol each woman, provide her with two sessions of virtual counselling, and capture her outcomes is shorter than may have been ideal.

For peer review only

For peer review only - http://bmjopen.bmj.com/site/about/guidelines.xhtml

INTRODUCTION

Anaemia in pregnancy is a complex public health problem that leads to severe adverse maternal and child health outcomes, including maternal morbidity and mortality [1-4] and impaired infant growth [5]. No country in South Asia is on course to meet the World Health Assembly target of a 50% reduction in anaemia by 2025 [6] and estimates from Nepal's 2016 Demographic and Health Survey indicate that 46% of pregnant women were anaemic (haemoglobin concentration < 11 g/dL) [7] – almost no change from the 2011 estimate of 48% [8].

Despite the lack of progress in reducing anaemia in pregnancy, and its multifactorial aetiology [9-11], there are several effective interventions that could help. Systematic reviews of randomised controlled trials show that consumption of IFA supplements and deworming with anti-helminthics in pregnancy can both lower the risk of maternal anaemia – by 70% and 15% respectively [12 13]. There are additional health benefits of IFA supplementation beyond anaemia, including increased birth weight [14-16], child survival [17] and development [18].

Provision of free-of-cost IFA and anti-helminthics have been scaled up in Nepal from 20 weeks' gestation as part of routine care in Nepal. The challenge lies in supporting women to access and use these evidence-based interventions [19]. Current access to recommended antenatal interventions is improving but remains sub-optimal. In 2016, 41% of women took iron tablets on at least 180 days of pregnancy, 69% took anti-helminthics, and the same proportion had four antenatal clinic (ANC) visits [8].

Although these aspects of ANC have improved markedly between 2011 and 2016 [7 8], the COVID-19 pandemic threatens to compromise this progress [20]. The pandemic has seen a sharp rise in neonatal and maternal mortality, along with declines in institutional deliveries and quality of care [21 22]. In Kapilbastu district of Nepal during the early phase of the pandemic in 2019/20, Maternal Mortality Ratio increased from 0.0 to 14.4 per 100,000 live births. Between 2018 and 2020, having at least 1 ANC visit decreased from 84% to 74% and having four ANC visits decreased from 63% to 52%, which would likely decrease uptake and adherence to IFA [23-26]. Repeated lockdowns, concerns about going to a health facility, discrimination against frontline workers (Female Community Health Volunteers, FCHVs) and loss of livelihoods could all pose barriers to women's access to ANC and IFA [22].

Nutrition education and counselling interventions have had variable effects on maternal anaemia [27]. In Nepal, the complexity of factors limiting access to adequate nutrition and IFA require gender-sensitive, tailored, participatory counselling methods, rather than a 'one-size-fits-all' didactic educational approach [28-30]. A recent trial of a social norms intervention in India found large effects

For peer review only

on consumption of iron-folic acid consumption, suggesting that such an approach holds promise [31].

A question remains as to whether and how these types of interventions can be adapted to reach the most geographically and socially isolated, who have least access to antenatal and social support. Virtual interventions may offer a solution, with almost all (93%) of Nepalese households now owning a mobile phone [7]. Systematic reviews have shown that 'eHealth' and 'mHealth' can improve a range of nutrition outcomes, including physical activity and healthy eating behaviour [32], but research has been concentrated in high-income contexts. Use of telemedicine for maternal and newborn care during the pandemic has been beneficial, but difficulties in reaching high-risk groups and maintaining quality of maternity services remain in low-income countries [33]. In rural Nepal, virtual antenatal counselling has the potential to address the shortage of healthcare professionals, and transportation and travel time barriers to accessing health care [34].

STUDY AIMS AND HYPOTHESES

In our study, designed in accordance with the SPIRIT guidelines [35], we test whether a virtual counselling intervention offered to pregnant women living in Kapilbastu could offer a feasible, affordable, scalable, and equitable solution to increase consumption of iron-folic acid supplements.

The primary objective of the Virtual Antenatal Intervention for Improved Diet and iron intake (VALID) trial is to assess whether providing pregnant women with an antenatal virtual counselling intervention, in addition to usual government services, increases women's compliance to IFA supplementation, compared with pregnant women who only had access to usual government services. Compliance is defined as: IFA consumed on 12 out of the last 14 days before the endline interview (which is 49-70 days after baseline). We hypothesised that the antenatal virtual counselling intervention would increase pregnant women's compliance to IFA tablets.

Secondary objectives are as follows:

1. To assess whether providing an antenatal virtual counselling intervention to pregnant women improves

- Dietary diversity,
- Timing and number of antenatal visits (ANC),
- Consumption of recommended iron-rich foods
- Practice of iron absorption-enhancing behaviours
- Knowledge of iron-rich foods and anaemia risks in pregnancy.

For peer review only - http://bmjopen.bmj.com/site/about/guidelines.xhtml

- Knowledge of COVID symptoms, prevention, and vulnerable population groups

2. To evaluate the feasibility, acceptability, equity, cost, cost-effectiveness and reach of the antenatal virtual counselling intervention (using an Implementation Science approach)

METHODS AND ANALYSIS

VALID trial design and setting

This is a non-blinded two-arm individually randomised controlled trial, with an allocation ratio of 1:1, conducted in Kapilbastu district, Lumbini Province of Nepal.

Trial arms are:

1. Control: access to routine antenatal care (ANC).
2. 'Virtual Antenatal Counselling': comprising of two interactive virtual counselling sessions provided to pregnant women in mid pregnancy, using 'zoom' on a tablet, in addition to routine ANC.

The trial is being implemented in 54 clusters in Kapilbastu district, Lumbini Province of Nepal, in the Western Terai (plains) of Nepal, bordering Uttar Pradesh state of India. The district population is 569,844 and literacy rates of women and men are 45% and 65% respectively [36]. Hinduism is the main religion, followed by Islam and the predominant ethnicity is Madhesi, many of whom speak Awadhi language. Anaemia prevalence is high (45%) and IFA in pregnancy is sub-optimal: 43% took at least the recommended dose of 180 IFA tablets, 33% took 60-179 tablets, and 24% took <60 tablets [7].

Eligibility

Study clusters (in Figure 1) which rural areas of southern Kapilbastu district that do not adjoin the main East-West highway that traverses Nepal; with no major market; 1100 -3199 projected population; surrounded with a buffer zone of non-study clusters; and >50% Madhesi (plains ethnicity) as per the pre-trial census.

Figure 1. Map of study clusters

Pregnant women are eligible for a baseline survey if they are aged 13-49 years, able to respond to questions, reside in a study cluster, and consent to participate. Additional inclusion criteria for enrolment in the trial include: 12 to 28 weeks' gestation (estimated from recall of last menstrual period or expected date of delivery given by a health worker), does not plan to leave the country

For peer review only

For peer review only - http://bmjopen.bmj.com/site/about/guidelines.xhtml

during the 5 weeks since enrolment, and no other pregnant woman in her household already enrolled in the trial.

To enrol participants, interviewers identify pregnant women in their study areas with help from FCHVs. They confirm eligibility, take written consent, and then phone the office to receive the random allocation.

Randomisation

A stratified block randomisation process is used to allocate pregnant women enrolled into the trial intervention or control arm. To balance the trial arms by two strong pre-identified determinants of IFA consumption, parity and baseline IFA consumption status, randomisation is done within each of four strata: (IFA yes/1st pregnancy); (IFA Yes/ not 1st pregnancy); (IFA no/1st pregnancy) and (IFA no/ not 1st pregnancy). For each stratum a separate allocation sequence is prepared. To make the sequence unpredictable, random permutation of the allocations within blocks is conducted and block sizes vary randomly between sizes 8, 6 and 4 using “blockrand” package in R programming software. Then, sequential allocations are sealed into sequentially numbered opaque envelopes by the project coordinator in Kathmandu, transported to the field office, stored in a locked cabinet, and opened by the monitoring and evaluation manager when the enumerators call to find out a pregnant woman’s allocation at the end of the baseline interview. After assignment of pregnant women to study arms, blinding of trial staff and participants is impossible, since interviewers distribute of tablet devices to pregnant women in the intervention arm.

Intervention

After enrolment, interviewers call pregnant women in the intervention arm to schedule appointments to deliver the tablets and orientate women on how to use the tablets. Tracking of tablet delivery is made by entering this information onto CommCare. Each tablet holds a sim card and has a data package which is topped up as necessary.

Enrolled women are assigned to one of ten counsellors, who are recruited and trained auxiliary nurse midwives. Counsellors call pregnant women, using case lists provided in the CommCare app on their tablets, to schedule their tailored virtual antenatal counselling sessions via Zoom. Pregnant women are offered their first counselling session after enrolment, at 12 to 28 weeks’ gestation, and the second counselling session is around two weeks later, at 14 to 31 weeks’ gestation. A diagram summarising the virtual counselling intervention is shown in Figure 2.

The counselling aims to support women and their families to take actions to change dietary practices, take IFA and deworming tablets, and access ANC. Counselling is conducted with pregnant women and their families to support them to think critically about the causes of anaemia

For peer review only - http://bmjopen.bmj.com/site/about/guidelines.xhtml

in pregnancy in their household and community, using stories and inductive questioning to trigger dialogue and reflection [37]. Stories directly address issues identified from formative research [38]. At each session, pregnant women (and their families if they attend) are engaged in a cycle of action and reflection whereby they discuss common issues that affect women in pregnancy and examples of actions for improving intake of IFA and deworming tablets, accessing ANC, and dietary practices. At the end of the first counselling session, the pregnant women and families make specific action plans to address the issues that are relevant for their family. In the second counselling session these action plans are reviewed and further discussed to support women and their families to address the issues and a second action plan is made. The tablets are collected back by interviewers after completing two counselling sessions.

Figure 2: Virtual counselling intervention model

The auxiliary nurse midwives received in-depth training on how to engage families in problem solving, the dialogical approach, and how to use a reference manual with examples of actions that pregnant women and their families could take. The training also provided counsellors with knowledge on anaemia consequences and prevention, and logistical training on how to use the tablets and record their progress.

Adherence to intervention protocols is ensured by monitoring the counselling (by supervisors joining remotely), by video-recording sessions, and by requiring counsellors to provide copies of action plans and to fill paper and electronic forms about each counselling session. Counsellors manage workload by prioritizing women approaching 28 weeks' gestation and filling a "phone call log" in CommCare to record outcome of each call.

Participants are free to seek concomitant care during pregnancy irrespective of their trial allocation.

Outcomes

The primary outcome of the trial is the proportion of pregnant women consuming IFA on at least 80% of the previous 2 weeks (i.e., on >=12 out of 14 days recalled). Other outcomes are given in Table 1.

Table 1. VALID outcome measures

For peer review only - http://bmjopen.bmj.com/site/about/guidelines.xhtml

VALID TRIAL OUTCOMES	Recall period	Definition	Variable type	Effect measure to compare arms / summary statistic
**PRIMARY OUTCOME**				
Proportion of pregnant women complying with recommended iron and folic acid tablet (IFA) intake	14 days	IFA consumed on 12 or more days out of the previous 14 days (i.e. on at least 80% of days)	Binary	Difference in Proportion
**SECONDARY OUTCOMES**				
Dietary diversity	24 hours	Count of the number of food groups consumed in the previous 24 hours preceding the endline interview, assessed using the list-based method, out of 10 food groups, as defined by (ref)	Count (0 to 10)	Difference in Mean
Consumption of intervention-promoted foods	24 hours	Any consumption of: green leafy vegetables, meat or fish	Binary	Difference in Proportion
Practicing one or more to enhance bioavailability	7 days	Recalled one or more of the following: using lemon or other vitamin C-rich foods with meals, eating sprouted grains or pulses, avoiding tea/coffee 1 hr either side of meals, or spreading meat-eating over two eating occasions rather than one.	Binary	Difference in Proportion
Knowledge of iron-rich foods	N/A	Count of iron-rich food groups correctly recalled	Count (0 to 9)	Difference in Mean
ANC visits	Baseline to endline	Count of Antenatal (ANC) visits between enrolment and endline interview	Count (0 to 6)	Difference in Mean
**EXPLORATORY OUCOMES**				
Understanding of why blood tests are taken at antenatal check-ups.	N/A	Proportion of those women who had a blood test at ANC who could correctly explain one or more reason for having a blood test.	Binary	Difference in Proportion
Knowledge of COVID-19 symptoms, precautions and vulnerability	N/A	Proportion of women who could correctly identify at least one vulnerable group, at least 3 COVID	Binary	Difference in Proportion

For peer review only

		control/prevention measures and at least 3 COVID symptoms		
ANC visits at the right time for her gestational age	Pregnancy to date	Whether the woman had her ANC visits at 2, 4, 6, and 8 months or not		

Outcome measurement

Interviewers measure outcomes on trial participants at enrolment (12 to 28 weeks' gestation) and endline (ideally 49 to 70 days after baseline, otherwise up to delivery). All outcomes are recalled by trial participants during interviews and recorded in electronic questionnaires. Additionally, at baseline we collect the woman's age, parity, medical history, date of the last menstrual period, pregnancy symptoms/ problems, and other key socioeconomic and demographic information. The data collection tools are programmed onto mobile devices in Nepali and English on Android operating system tablets or mobile phones using the CommCare electronic data collection platform [39]. These have in-built jump-sequences and value limits to prevent entry of data outside plausible ranges. As interviewers are fluent Awadhi speakers but do not read in Awadhi, they conduct the interviews by reading the question in Nepali and wording it into Awadhi to the respondent. Standard Awadhi wording is agreed upon within the team. Interviewers follow standard procedures for 24-hour dietary recall measurements and IFA recall to ensure that measurements are accurate and inter-observer difference minimised.

The monitoring team meets monthly and Kathmandu-based team members provide support as needed. Interviewers log any problems with electronic forms and the data team make corrections reversibly in the data using Stata data cleaning 'do' files as required.

Sample size

Our target sample size is 300 pregnant women, 150 in each arm. With at least 270 followed up (90% follow-up rate in each arm), we have 80% power to detect a 15 percentage-point increase in IFA compliance (the proportion of women consuming IFA on at least 12 out 14 days preceding the endline survey) if the control arm compliance prevalence is 66-68% (or 16.7 percentage point difference if control arm compliance is only 50%).

To achieve the required sample size, interviewers communicate with the Female Community Health Volunteers (FCHVs) in the study area. In line with government norms, FCHVs are provided with an incentive per day spent identifying pregnant women for our trial. To increase the response rate in later pregnancy, we provide an incentive (200 Nepali rupees mobile phone top-up) to trial

For peer review only - http://bmjopen.bmj.com/site/about/guidelines.xhtml

participants after the endline interview. Target dates for enrolment and endline interviews, based on gestational age and date of enrolment, are displayed on customised lists in CommCare on interviewers' mobile devices.

Statistical analysis

The trial statistician (AC) and Nepal principal investigator (NS) will make a Statistical Analysis Plan, to detail the analysis strategies, covariate adjustment, and the approach to any missing data. We will present this to the Trial Steering Committee (TSC) and Data Monitoring Committee (DMC) for approval before analysis. Primary analysis will be replicated by a second analyst.

Analyses will involve graphically presenting the number of days of IFA consumption in last 14 days by arm (bar chart) at baseline and follow-up by trial arm. Primary Analysis will be by intention-to-treat using two-sided testing at the standard 5% significance level. Analysis of our binary primary outcome, 12+ days consumption of IFA in last 14 days at follow-up, is based on logistic regression adjusting for baseline consumption in the previous 14 days, gestational age at interview and parity (note the randomisation strata are based on parity and baseline consumption). Our 'fully adjusted' results (secondary approach) will include additional adjustment for wealth score constructed out of household assets, education status of pregnant woman and age.

We will follow a similar methodology for the analysis of secondary and exploratory outcomes (given in Table 1), and process indicators to be reported in a separate publication. We will use linear regression for continuous outcomes, logistic regression for binary outcomes, and negative binomial regression for 'count' outcomes such as number of ANC visit between baseline and endline. We will examine the distribution of continuous and count outcomes before analysis of intervention effectiveness.

We plan to also report the intervention effect stratified by consumption of IFA at baseline, and test for an interaction with intervention arm. Within the intervention arm we will report and test the association between the degree of intervention exposure (number of counselling sessions received) and IFA consumption at endline. Other subgroup analyses will compare the intervention effect on the primary outcome by socio-economic status (wealth quintile), education and exposure to ANC.

Whilst our main analyses are under the intention-to-treat principle, for the primary outcome we will also conduct a 'per protocol' analysis in which only women who received virtual counselling are compared to all participants in the control arm.

For peer review only

We expect very low missing data in our baseline indicators, but we anticipate some women will not have a primary outcome. We consider data as missing where the woman is unavailable, has moved away, or withdrew consent but we do not regard data as 'missing' if due to miscarriage. We are not planning imputation of the primary outcome since there is little information on which to base the imputation other than baseline IFA consumption, which we are including as a covariate in our regression models.

Data management

We use CommCare's case management system that enables interviewers and counsellors to follow up pregnant women for data collection and intervention and promotes participant retention. Interviewers and FCHVs hold paper lists of potential pregnant women prior to enrolment. Data collected on electronic forms are locally stored before they are synchronised (encrypted) to the CommCare cloud server.

The data manager in Kathmandu downloads the data daily from the CommCare cloud server onto their server, imports the data into Stata and runs 'do' files for data pseudonymising, labelling and recoding. The data are stored on password-protected, encrypted, secure server computers in lockable rooms at the HERD International Kathmandu office. We back up data from physical servers onto secure cloud servers daily, and onto external hardware-encrypted hard drives each week. Person-identifiable data are stored in separate encrypted files and are only used to generate follow-up lists for authorised field team members. All other data are pseudonymised. Study arm is encoded but not labelled. After data collection is completed, we will delete VALID data from laptops, tablets, and the CommCare server.

For qualitative data collected in Awadhi and Nepali languages, qualitative interviewers transcribe the data in Nepali. They then send audio recordings and transcriptions to the qualitative data officer who checks completeness, anonymises, and stores the data in a lockable cabinet.

Data cleaning is completed by identifying outliers and removing as required. HERD International follows international and NHRC policies for archiving of data in Nepal. We will make the pseudonymised dataset open access by using the UCL data sharing platform as per MRC guidelines. Any request for archived person-identifiable data will go through the trial management team in alignment with provisions in the consent forms.

No storable biological specimens are collected during this study.

Process evaluation

For peer review only

We are conducting a mixed methods process evaluation to describe the feasibility, acceptability, equity, reach, and fidelity to plans, as guided by the theory of change [38 40] in Figure 3.

Figure 3: Theory of Change for the VALID intervention

Quantitative process outcomes are listed in Table 2.

Table 2. Quantitative process evaluation outcomes

Domain	Measure	Data source
Acceptability	Proportion of pregnant women who:<br>1) were satisfied with virtual counselling session<br>2) consented to participate in the trial out of those eligible at baseline<br>3) had family member(s) attending the virtual counselling session<br>4) reported the tablet and video link is an acceptable way to receive counselling and advice during pregnancy	Endline interview
Effectiveness	5) received satisfactory support from family members<br>6) implemented the action plan<br>7) found the intervention useful<br>8) understood what was said during the virtual counselling session<br>9) needed support to use the tablet and video link<br>10) reported constant internet disruption for smooth conversation during virtual counselling session<br>11) felt confident in their nutrition knowledge and/or able to take actions to improve nutrition/health	Endline interview
Feasibility	12) completed 2 virtual counselling sessions<br>13) had direct access to a phone/device<br>14) were easily able to use the tablet and video link<br>Number of attempts to contact participant when intervention not possible	Endline Interview<br><br>Phone call log
Fidelity	Proportion of Counsellors adhering to the virtual counselling manual guidance	Observations of sessions
Coverage and Reach	Proportion of<br>• pregnant women enrolled out of those approached<br>• marginalised Muslim and Dalit castes women enrolled out of those approached<br>• primiparous women enrolled of those approached	Baseline interview

For peer review only

For peer review only - http://bmjopen.bmj.com/site/about/guidelines.xhtml

	• young (<18 years of age) enrolled of those approached	
Sustainability	Proportion of organizations (government and non-governmental) attending the dissemination workshop willing to implement the intervention	Dissemination workshop
Cost	Total intervention cost from program provider perspective<br>Total intervention costs by line items/inputs<br>Cost of Intervention per PW	Costing tools and time allocation sheets

We will use qualitative data (semi-structured interviews) to explore how the context may influence intervention effect [38]. We will analyse post-counselling forms and observe virtual counselling sessions to describe intervention implementation and fidelity to plans. Further, we will conduct focus group discussions with supervisors and counsellors to explore how context affects the intervention and analyse factors affecting the families' interaction during the counselling sessions. We will interview 20 pregnant women who received the intervention, purposively sampled based on the number of children they have, and their socio-economic status. We will also interview 15 family members of different pregnant women (husbands and mothers-in-law) who did and did not participate in the intervention to explore the factors affecting their participation and their perceptions of the intervention. To consider the sustainability of the intervention we will show a piloted virtual counselling session to health workers in two health facilities and discuss the feasibility and acceptability of using virtual counselling within the health system and community. In addition, we will interview 2 FCHVs to explore the factors affecting recruitment and enrolment of women and their observation on others' (especially participants') perceptions of the intervention.

Cost effectiveness

We will estimate cost and cost-effectiveness of the intervention from a provider perspective by estimating the costs of designing and implementing the virtual counselling (program costs) and costs to public health care providers. We collect program cost data from HERD international project accounts, staff time use surveys, and interviews with project staff. We estimate costs to public health care providers resulting from any increased demand for health services caused by the intervention, using ANC and IFA data from the endline interview and any available secondary data on unit costs for these services. We will calculate incremental cost effectiveness of virtual

For peer review only - http://bmjopen.bmj.com/site/about/guidelines.xhtml

counselling compared to routine care and estimate incremental cost-effectiveness ratios (ICERs) for the primary outcome.

Trial monitoring and safety

We do not expect virtual counselling to have negative effects, although we have an established complaints procedure. Asking women to discuss their pregnancy may be difficult or embarrassing; participants can stop an interview or counselling at any time. Counsellors are trained to advise on how to mitigate minor IFA side-effects, such as constipation and indigestion, or conflict in the household from provision of tablets [2 41]. If the counsellor or interviewer observes any pregnancy danger signs or the woman is feeling unwell at the time of interaction, they advise the woman to seek care from the nearest health facility, as per the government's standard treatment protocol [42]. The virtual counselling intervention is designed to limit the spread of COVID-19, but interviewers need to visit participants' home to provide the tablets. We removed measurements requiring physical contact (anthropometry and haemoglobin) from our protocol to enable interviewers to keep 2 metres from household members, except while demonstrating use of the tablet. Field staff are required to adhere to COVID-19 Infection Prevention and Control (IPC) protocols. Counsellors and interviewers record adverse events (including maternal deaths or COVID-19 cases) and inform field managers who submit an adverse events form to the PIs. As the trial follow-up period is short, we will not have interim audits. We do not expect to end the counselling early (unless the COVID-19 pandemic renders activities impossible), and we do not envisage ancillary or post-trial care will be needed beyond routine ANC.

ETHICS AND DISSEMINATION

We have obtained ethical approval from Nepal Health Research Council (NHRC approval number: **570/2021**) with amendment approval on 9 March 2022, UCL ethics committee (Project ID number: **14301/001** with amendment approval on 6 May 2022). We will seek approval for any major changes to the protocol from the Trial Steering Committee and ethics boards and amend the registry.

General consent was sought from municipality leaders, health system managers, and ward representatives. Interviewers seek household heads' oral consent to speak with pregnant women, then take written consent/assent from pregnant women. Additional consent is obtained from guardians if the participant is aged <18 years. After assessing eligibility, the interviewers take consent to enrol eligible pregnant women into the trial by signature or thumbprint. In subsequent

For peer review only - http://bmjopen.bmj.com/site/about/guidelines.xhtml

visits to pregnant women, interviewers and counsellors take oral consent. Information sheets and consent forms in English are available in Supplementary Annex 1 (copies in Nepali and Awadhi are available on request).

Our consent forms ask permission to share the anonymised data with other researchers for secondary analyses and to revisit the participant for follow-up. The final data will be held by UCL and HERD International on secure servers. The analysis dataset and statistical code will be shared in a web annex to the main trial publication.

Patient and Public Involvement

We conducted qualitative formative research with reproductive age women, mothers-in-law, fathers and FCHVs to inform the design of the study, using findings to refine our research questions/priorities and shape the intervention [29]. We did not involve patients/public in the choice of outcomes but involved FCHVs and health workers in participant recruitment. When disseminating study results, we will engage trial participants and municipal leaders from the study clusters as well as national and provincial policy makers, government officials, academics, and other relevant stakeholders. We will prepare a policy brief in English and Nepali and distribute it at dissemination workshops.

We will report results to NHRC within one year of the end of the study, publish our results in peer-reviewed journals and present findings at national, regional, and international conferences where possible.

CURRENT TRIAL STATUS

The trial timeline is provided in Figure 4.

Figure 4. Schedule of enrolment, interventions, and assessments

Interviewers enrolled women from 14 January until 23 February 2022. Endline interviews began on 9 March 2022 and are due to be completed by 19 June 2022, depending on the availability of participants.

Supplemental Annex 1. Consent form and participant information sheet

Abbreviations

Antenatal care (ANC)

For peer review only

Demographic and Health Survey (DHS)

Data Monitoring Committee (DMC)

Female Community Health Volunteer (FCHV)

Health Research and Social Development Forum, Nepal (HERD)

International Standard Randomised Controlled Trial Number (ISRCTN)

Iron and Folic Acid supplement (IFA)

Medical Research Council (MRC)

Nepal Health Research Council (NHRC)

Novel SARS-CoV-2 Corona Virus (COVID-19)

Trial Management Group (TMG)

Trial Steering Committee (TSC)

University College London (UCL)

Acknowledgements

We thank Suprich Sapkota, Basudev Bhattarai, Avinash Bhurtel, Anjali Basnet, and Samata Yadav for their contributions in development of this protocol.

Contributors

NS led the development of the protocol and the writing of the manuscript draft. SB gave oversight to field management all trial processes and drafted the process evaluation and randomisation processes. SG oversaw data collection tools with NS. JM, SM, AA designed the intervention and process evaluation. AC, NS, HHF, SG designed the trial methodology, sample size calculation, and data analysis plan, and outcome measurement. HHB designed the economic evaluation. SB, SCB, SH, NS provided overall leadership. All authors provided inputs and read and approved the final paper.

Provenance and peer review

Not commissioned, externally peer reviewed.

References

1. Daru J, Zamora J, Fernandez-Felix BM, et al. Risk of maternal mortality in women with severe anaemia during pregnancy and post partum: a multilevel analysis. *The Lancet Global health*

For peer review only

2018;6(5):e548-e54. doi: 10.1016/S2214-109X(18)30078-0 [published Online First: 20180320]

2. Haider BA, Olofin I, Wang M, et al. Anaemia, prenatal iron use, and risk of adverse pregnancy outcomes: systematic review and meta-analysis. *BMJ* 2013;346:f3443. doi: 10.1136/bmj.f3443 [published Online First: 2013/06/26]

3. Black RE. Global Distribution and Disease Burden Related to Micronutrient Deficiencies. *Nestle Nutrition Institute workshop series* 2014;78:21-28. doi: 10.1159/000354932

4. Khan KS, Wojdyla D, Say L, et al. WHO analysis of causes of maternal death: a systematic review. *The Lancet* 2006;367(9516):1066-74. doi: https://doi.org/10.1016/S0140-6736(06)68397-9

5. Kozuki N, Lee AC, Katz J, et al. Moderate to severe, but not mild, maternal anemia is associated with increased risk of small-for-gestational-age outcomes. *J Nutr* 2012;142(2):358-62. doi: 10.3945/jn.111.149237 [published Online First: 2011/12/23]

6. Global Nutrition Report. The state of global nutrition. Bristol, UK.: Development Initiatives, 2021.

7. Ministry of Health Nepal, New ERA, ICF. Nepal Demographic and Health Survey 2016. : Ministry of Health, Nepal, 2017.

8. Ministry of Health and Population (MOHP) [Nepal] NE, and ICF International Inc. . Nepal Demographic and Health Survey 2011: Ministry of Health and Population, New ERA, and ICF International, Calverton, Maryland., 2012.

9. Pasricha S-R, Armitage AE, Prentice AM, et al. Reducing anaemia in low income countries: control of infection is essential. *BMJ* 2018;362:k3165.

10. Camaschella C. Iron deficiency: new insights into diagnosis and treatment. *Hematology Am Soc Hematol Educ Program* 2015;2015:8-13. doi: 10.1182/asheducation-2015.1.8 [published Online First: 2015/12/08]

11. Petry N, Olofin I, Hurrell RF, et al. The Proportion of Anemia Associated with Iron Deficiency in Low, Medium, and High Human Development Index Countries: A Systematic Analysis of National Surveys. *Nutrients* 2016;8(11):693. doi: 10.3390/nu8110693 [published Online First: 2016/11/10]

12. Pena-Rosas JP, De-Regil LM, Garcia-Casal MN, et al. Daily oral iron supplementation during pregnancy. *Cochrane Database Syst Rev* 2015(7):CD004736. doi: 10.1002/14651858.CD004736.pub5 [published Online First: 20150722]

For peer review only

13. Salam RA, Das JK, Bhutta ZA. Effect of mass deworming with antihelminthics for soil-transmitted helminths during pregnancy. *Cochrane Database Syst Rev* 2021;5:CD005547. doi: 10.1002/14651858.CD005547.pub4 [published Online First: 20210517]

14. Malhotra N, Upadhyay RP, Bhilwar M, et al. The role of maternal diet and iron-folic acid supplements in influencing birth weight: evidence from India's National Family Health Survey. *J Trop Pediatr* 2014;60(6):454-60. doi: 10.1093/tropej/fmu051 [published Online First: 2014/10/01]

15. Imdad A, Bhutta ZA. Routine iron/folate supplementation during pregnancy: effect on maternal anaemia and birth outcomes. *Paediatr Perinat Epidemiol* 2012;26 Suppl 1:168-77. doi: 10.1111/j.1365-3016.2012.01312.x

16. Cogswell ME, Parvanta I, Ickes L, et al. Iron supplementation during pregnancy, anemia, and birth weight: a randomized controlled trial. *Am J Clin Nutr* 2003;78(4):773-81. doi: 10.1093/ajcn/78.4.773 [published Online First: 2003/10/03]

17. Christian P, Stewart CP, LeClerq SC, et al. Antenatal and postnatal iron supplementation and childhood mortality in rural Nepal: a prospective follow-up in a randomized, controlled community trial. *Am J Epidemiol* 2009;170(9):1127-36. doi: 10.1093/aje/kwp253 [published Online First: 2009/09/26]

18. Perez EM, Hendricks MK, Beard JL, et al. Mother-Infant Interactions and Infant Development Are Altered by Maternal Iron Deficiency Anemia. *The Journal of Nutrition* 2005;135(4):850-55. doi: 10.1093/jn/135.4.850

19. Yadav KD, Yadav UN, Wagle RR, et al. Compliance of iron and folic acid supplementation and status of anaemia during pregnancy in the Eastern Terai of Nepal: findings from hospital based cross sectional study. *BMC Research Notes* 2019;12(1):127. doi: 10.1186/s13104-019-4167-6 [published Online First: 20190312]

20. Singh DR, Sunuwar DR, Adhikari B, et al. The perils of COVID-19 in Nepal: Implications for population health and nutritional status. *J Glob Health* 2020;10(1):010378. doi: 10.7189/jogh.10.010378

21. KC A, Gurung R, Kinney MV, et al. Effect of the COVID-19 pandemic response on intrapartum care, stillbirth, and neonatal mortality outcomes in Nepal: a prospective observational study. *The Lancet Global Health* 2020 doi: 10.1016/s2214-109x(20)30345-4

22. Singh R, Subedi M. COVID-19 and stigma: Social discrimination towards frontline healthcare providers and COVID-19 recovered patients in Nepal. *Asian J Psychiatr* 2020;53:102222. doi: 10.1016/j.ajp.2020.102222 [published Online First: 20200613]

For peer review only - http://bmjopen.bmj.com/site/about/guidelines.xhtml

23. Ministry of Health and Population. Department of Health Services Annual Report 2076/77 (2019/20), 2020.

24. Ministry of Health and Population. Department of Health Services Annual Survey 2075/6 (2018/19), 2019.

25. Ministry of Health and Population. Nepal HMIS Local Level Raw Data 2076-77 (2019-20). Kathmandu, Nepal: Department of Health Services, 2020.

26. Ministry of Health and Population. Nepal HMIS Database 2075-76 (2018-2019) by Local Government. Kathmandu, Nepal: Department of Health Services 2019.

27. Girard AW, Olude O. Nutrition education and counselling provided during pregnancy: effects on maternal, neonatal and child health outcomes. *Paediatric and Perinatal Epidemiology* 2012;26 Suppl 1:191-204. doi: 10.1111/j.1365-3016.2012.01278.x [published Online First: 2012/07/07]

28. Morrison J, Dulal S, Harris-Fry H, et al. Formative qualitative research to develop community-based interventions addressing low birth weight in the plains of Nepal. *Public Health Nutr* 2018;21(2):377-84. doi: 10.1017/S1368980017002646 [published Online First: 2017/10/17]

29. Morrison J, Giri R, Arjyal A, et al. Addressing anaemia in pregnancy in rural plains Nepal: A qualitative, formative study. *Matern Child Nutr* 2021;17 Suppl 1:e13170. doi: 10.1111/mcn.13170 [published Online First: 2021/07/10]

30. Harris-Fry H, Saville NM, Paudel P, et al. Relative power: Explaining the effects of food and cash transfers on allocative behaviour in rural Nepalese households. *Journal of Development Economics* 2022;154:102784. doi: https://doi.org/10.1016/j.jdeveco.2021.102784

31. Rimal RN, Yilma H, Sedlander E, et al. Iron and folic acid consumption and changing social norms: cluster randomized field trial, Odisha, India. *Bull World Health Organ* 2021;99(11):773-82. doi: 10.2471/BLT.20.278820 [published Online First: 20210831]

32. Fiedler J, Eckert T, Wunsch K, et al. Key facets to build up eHealth and mHealth interventions to enhance physical activity, sedentary behavior and nutrition in healthy subjects–an umbrella review. *BMC Public Health* 2020;20(1):1-21.

33. Galle A, Semaan A, Huysmans E, et al. A double-edged sword-telemedicine for maternal care during COVID-19: findings from a global mixed-methods study of healthcare providers. *BMJ Glob Health* 2021;6(2) doi: 10.1136/bmjgh-2020-004575

34. Bhatta R, Aryal K, Ellingsen G. Opportunities and Challenges of a Rural-telemedicine Program in Nepal. *Journal of Nepal Health Research Council* 2015;13(30):149-53.

For peer review only - http://bmjopen.bmj.com/site/about/guidelines.xhtml

593 35. Chan A-W, Tetzlaff JM, Altman DG, et al. SPIRIT 2013 Statement: Defining Standard Protocol Items for Clinical Trials. *Ann Intern Med* 2013;158(3):200-07. doi: 10.7326/0003-4819-158-3-201302050-00583

596 36. Central Bureau of Statistics N. National Population and Housing Census 2011 (National Report). Kathmandu, Nepal: Government of Nepal, 2012.

598 37. Wallerstein N, Bernstein E. Empowerment education: Freire's ideas adapted to health education. *Health education quarterly* 1988;15(4):379-94. doi: 10.1177/109019818801500402

601 38. Moore GF, Audrey S, Barker M, et al. Process evaluation of complex interventions: Medical Research Council guidance. *BMJ* 2015;350:h1258.

603 39. CommCare [program], 2020.

604 40. Saville NM, Kharel C, Morrison J, et al. Comprehensive Anaemia Programme and Personalized Therapies (CAPPT): protocol for a cluster-randomised controlled trial testing the effect women's groups, home counselling and iron supplementation on haemoglobin in pregnancy in southern Nepal. *Trials* 2022;23(1):183. doi: 10.1186/s13063-022-06043-z [published Online First: 20220301]

609 41. Gautam S, Min H, Kim H, et al. Determining factors for the prevalence of anemia in women of reproductive age in Nepal: Evidence from recent national survey data. *PLoS One* 2019;14(6):e0218288. doi: 10.1371/journal.pone.0218288 [published Online First: 20190612]

613 42. Department of Health Services. Standard Treatment Protocols for Health Posts and Sub-Health posts. Kathmandu, Nepal: Ministry of Health and Population, 2012.

615

For peer review only

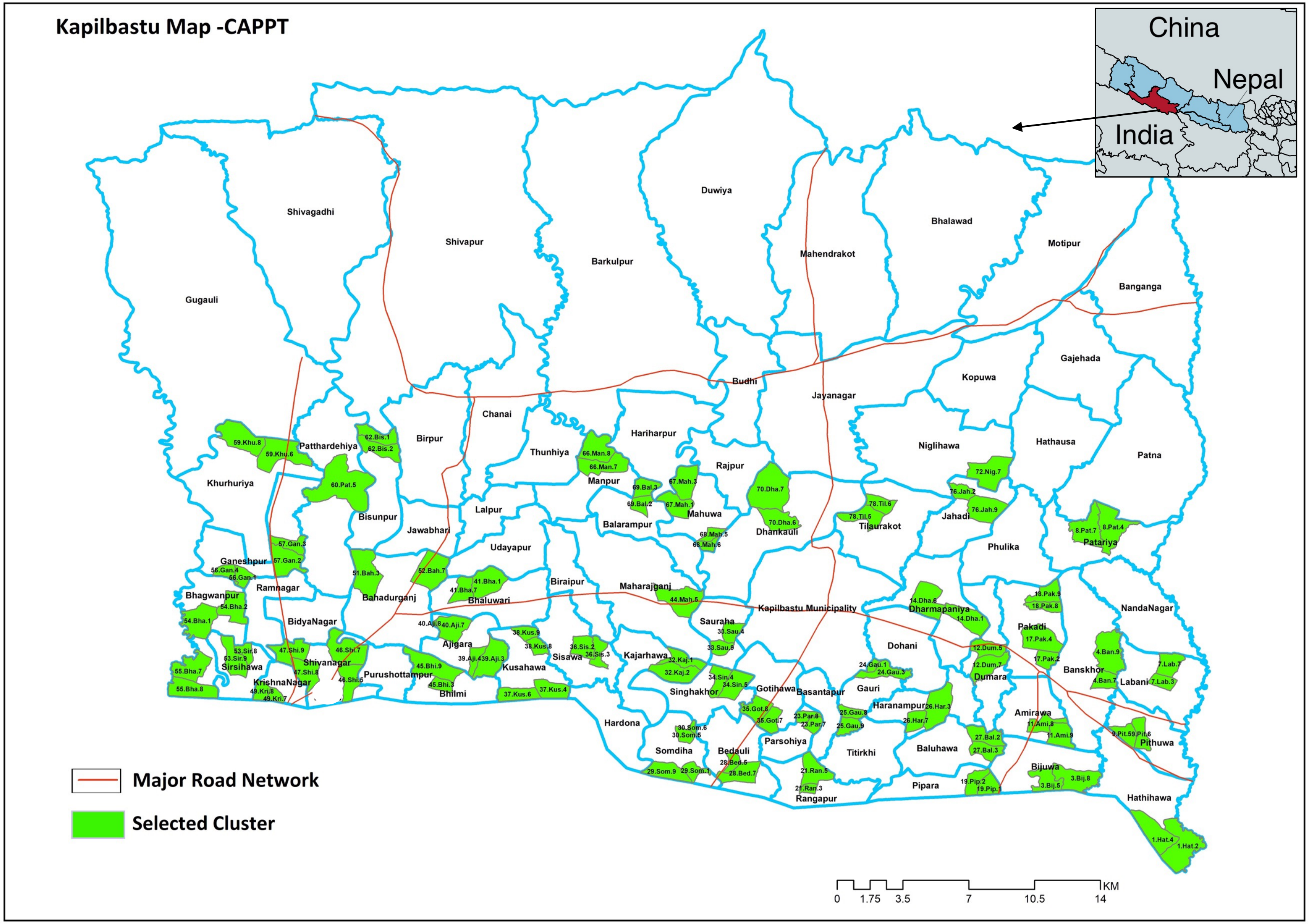
Kapilbastu Map -CAPPT
China
Nepal
India
Shivagadhi
Shivapur
Barkulpur
Duwiya
Bhalawad
Mahendrakot
Motipur
Banganga
Gugauli
Gajehada
Kopuwa
Budhi
Jayanagar
Chanai
Birpur
Hariharpur
Thunhiya
Niglihawa
Hathausa
Patna
Patthardehiya
Khurhuriya
Rajpur
Manpur
Mahuwa
Bisunpur
Lalpur
Jawabhari
Balarampur
Dhankauli
Tilaurakot
Jahadi
Phulika
Patariya
Udayapur
Ganeshpur
Ramnagar
Bahadurganj
Bhaluwari
Biraipur
Maharajganj
Kapilbastu Municipality
Dharmapaniya
NandaNagar
Bhagwanpur
BidyaNagar
Sauraha
Pakadi
Dohani
Ajigara
Shivanagar
Sirsihawa
Sisawa
Kajarhawa
Kusahawa
Purushottampur
KrishnaNagar
Bhilmi
Singhakhor
Gotihawa
Basantapur
Gauri
Dumara
Banskhor
Labani
Haranampur
Amirawa
Hardona
Somdiha
Bedauli
Parsohiya
Titirkhi
Baluhawa
Pithuwa
Rangapur
Pipara
Bijuwa
Hathihawa
Major Road Network
Selected Cluster
0 1.75 3.5 7 10.5 14 KM

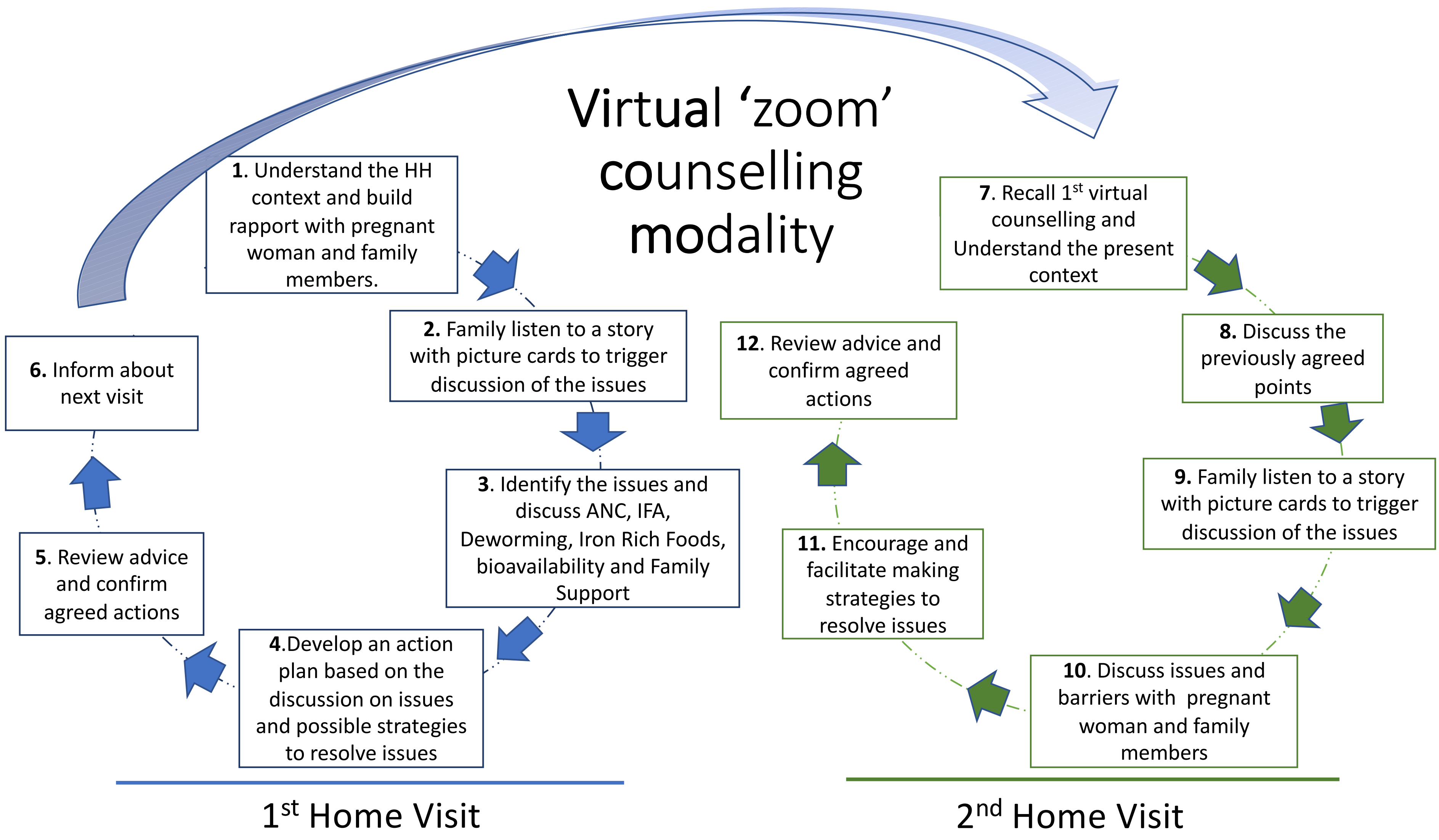
Virtual 'zoom' counselling modality
1. Understand the HH context and build rapport with pregnant woman and family members.
2. Family listen to a story with picture cards to trigger discussion of the issues
3. Identify the issues and discuss ANC, IFA, Deworming, Iron Rich Foods, bioavailability and Family Support
4.Develop an action plan based on the discussion on issues and possible strategies to resolve issues
5. Review advice and confirm agreed actions
6. Inform about next visit
7. Recall 1st virtual counselling and Understand the present context
8. Discuss the previously agreed points
9. Family listen to a story with picture cards to trigger discussion of the issues
10. Discuss issues and barriers with pregnant woman and family members
11. Encourage and facilitate making strategies to resolve issues
12. Review advice and confirm agreed actions
1st Home Visit
2nd Home Visit

**Intervention**	**Pregnant woman and her household members** Dialogue and problem solving in two virtual 'zoom' counselling session on a tablet					
Awareness and health literacy (household members and pregnant woman)	Awareness of iron-rich food sources	Awareness of ways to improve bioavailability	Awareness of the need for iron-rich diet & iron and folic acid tablets (IFA)	Awareness of ways to reduce side-effects of iron and folic acid (IFA)	Awareness of role of HH in improving diets, increasing access to antenatal care and supporting IFA consumption	
Household actions to support Pregnant Woman	Encourage healthy snacking	Cooking and serving practices to increase Pregnant Women's access to iron-rich diet	Encourage / enable dietary practices to increase bioavailability of iron	Encourage / enable practices to minimise side-effects of IFA	Encourage / enable consumption of IFA	Accompany to ANC & retrieve re-supply of IFA
Enabling environment	Increased household support to Pregnant Woman		Men take a more active role in supporting Pregnant Woman		IFA and 'eating well' not linked with having a large baby	
Intermediate outcomes	Improved intake of iron-rich foods	Improved dietary diversity	Improved actions to increase iron absorption	Improved uptake of Antenatal Care	Improved access and uptake of IFA	Improved access to and uptake of de-worming
**Primary / secondary outcomes**	**Improved compliance in taking Iron and Folic Acid tablets** **Dietary modifications to increase iron consumption / absorption in Pregnant Woman**					
'Higher level' outcomes not measured	Improved dietary iron adequacy	Improved absorption of iron from diet	Improved overall dietary adequacy	Improved micronutrient status	Higher haemoglobin / Less anaemia	Improved physical health of mother and baby

Legend: IFA Iron Folic Acid

	STUDY PERIOD					
	Enrol-ment	Alloc-ation	Post-allocation			Close-out
**TIMEPOINT****	t_0 Baseline interview		t_1 (t_0 + 1 to 7 days or more[#])	t_2 (t_1 + 1 to 7 days or more[#])	t_3 (t_2 + 14 days or more[#])	t_x *Endline interview* (t_0 + 49 to 70 days ideally but permitted up to delivery[#])
**ENROLMENT at baseline interview:**						
Eligibility screen for baseline	X					
Informed consent for baseline	X					
Eligibility screen for trial	X					
Informed consent for trial	X					
**Allocation**		X				
**INTERVENTIONS:**						
Tablet handover and orientation			X			
Virtual counselling session 1				X		
Virtual counselling session 2					X	
**ASSESSMENTS:**						
Baseline survey variables:						
Socioeconomic indicators / food security						
Marriage / Pregnancy history						
Uptake / quality of antenatal care (ANC)						
Iron and Folic Acid (IFA) access/consumption	X					
Deworming access/consumption						
Dietary Diversity Score						
Knowledge and intake of key iron-rich foods						
Practice of iron-absorption enhancement						
Process variables						
**Post-counselling form**						
Ease of conducting counselling						
Who attended counselling?						
Pregnant woman's level of comfort				X	X	
Pregnant woman's attentiveness						
Engagement of family members						
Actions agreed upon with family						
**plus in Post-2nd counselling session only**						
Extent actions were implemented					X	
Factors preventing / facilitating taking actions						
Endline interview variables:						
Uptake / quality of antenatal care (ANC)						
Iron and Folic Acid (IFA) access/consumption						
Deworming access/consumption						
Dietary Diversity Score						
Knowledge and intake of key iron-rich foods						X
Practice of iron-absorption enhancement						
Confidence in health knowledge						
Exposure and response to intervention						
Awareness of COVID-19 prevention, vulnerability and symptoms						

[#] Note that although target follow-up periods were defined at outset, lack availability of respondents to attended counselling and/or interviews means that gaps between time points are varied.

For peer review only

Information sheet for VALID Baseline survey with Pregnant Women

Introduction

Namaste! My name is _______________________. I have come from HERD International located in Thapathali, Kathmandu. HERD International is a national level research organization. This organization has been conducting various programmes and research upon health, environment and social development. Currently, HERD International, in partnership with University College London, is conducting a study that aims to reduce anaemia in pregnant women in Kapilbastu. I would like to invite you to be a part of this study.

Before you decide whether to participate, it is important for you to understand why this research is being done and what participation will involve. I will read what is written in this information sheet aloud to you. You can ask me if there is anything that you do not understand or if you want more information. You will be given a copy of this information sheet. Take your time to decide whether or not you want to take part in the study or not. Thank you for reading this/listening to me.

Details of the Study

Anaemia is a condition when there is decreased haemoglobin in blood, it is associated with illness and complications during pregnancy and childbirth. HERD International, in partnership with University College London, is conducting a study with an aim to reduce anaemia in pregnant women. This study is aiming to improve anemia status among pregnant women in the community through virtual counselling session on nutrition, diet and antenatal care. Therefore, we would like to invite you to participate in this baseline survey we are conducting to understand your eligibility for the follow up study, socio demographics, dietary habits and antenatal care seeking behavior among pregnant women in the study area.

Why this survey?

We are conducting this study in 54 clusters or study areas in Kapilvastu district. Iron deficiency anemia is the main reason for anemia among pregnant women. Pregnant women who are anaemic are much more likely to die during childbirth than those women who are not and their infants are more likely to be born small for gestational age. During the baseline survey we will visit homes of all pregnant women in the study area to collect data, we expect to interview at least 1000 pregnant women, out of these women we hope to enroll at least 300 who are eligible (≤28 weeks' gestation) into the follow up study.

Why am I being invited to participate in this survey?

You can take part in this study if you are a pregnant woman or girl aged 13 to 49 years, live in the study area and are able to respond to the survey questions. If you are interested to take part in the study, we will give you this information sheet to read or have others read to you. Once you have listened-to (or read) and understood the information sheet we will ask you to give us sign the consent form agreeing to participate in the survey. If you decide to participate, we will ask personal questions about you and your family (for eg: household head name, family size, ethnicity, religion, income and education) as well as your dietary habits, health problems and utilization of health care.

Will my taking part in this project be kept confidential?

All the information that we collect about you during the course of the research will be kept strictly confidential. Your information will be recorded using tablets by the researchers are involved in finding you to interview you. Only they will have access to your name and address, all other researchers who look at the information you share with us will not be able to identify you as your name and address will be removed.

For peer review only

Ethical approval

This study has been approved by the Nepal Health Research Council Approval ID number 570/2021.

Agreeing to take part

Your participation is voluntary. If you don't want to take part, you can refuse without giving a reason. If you decide to take part in the study, you will be given this information sheet to keep and be asked to sign or thumb print the consent. If you agree to participate and then change your mind at any time, please tell us and we will stop the interview at any point.

Are there any risks if you participate?

We do not think that any harm will come to you, but it is possible that you might find sharing information about your pregnancy uncomfortable or upsetting. You don't have to continue to take part if you don't feel like it.

Are there any benefits if you participate?

There is no direct benefit of participating in this study. However, the findings from this study will generate evidence to help develop health and nutrition programs and policies for pregnant women in future.

Contact for further information

You are encouraged to ask any questions you wish, before, during or after getting involved. If you require any further information or have any concerns while taking part in the study you can contact:

HERD International, Prasuti Griha Marg, Thapathali, Kathmandu. Tel 01-4238045

HERD International District Office, Taulihawa, Kapilbastu. Tel: number: 076-590090

Thank you for listening to / reading this information sheet and for considering whether to take part in this research study.

For peer review only

Virtual Antenatal Intervention for improved Diet and Iron intake (VALID) Trial

Participant Information Sheet for Pregnant Women

Introduction

Namaste! My name is ________________________. I have come from HERD International located in Thapathali, Kathmandu. HERD International is a national level research organization. This organization has been conducting various programmes and research upon health, environment and social development. Currently, HERD International, in partnership with University College London, is conducting a study that aims to reduce anaemia in pregnant women in Kapilbastu. I would like to invite you to be a part of this study.

Before you decide whether to participate, it is important for you to understand why this research is being done and what participation will involve. I will read what is written in this information sheet aloud to you. You can ask me if there is anything that you do not understand or if you want more information. You will be given a copy of this information sheet. Take your time to decide whether or not you want to take part in the study or not. Thank you for reading this/listening to me.

Details of the study

HERD International, in partnership with University College London, is conducting a study with an aim to reduce anaemia in pregnant women. The Medical Research Council (UK) is funding this research.

Anaemia is a condition when there is decreased haemoglobin in blood, and this is caused by various factors. In Nepal, lack of iron is the most common cause of anaemia in pregnancy. It is important to reduce anaemia in pregnancy because low iron levels are associated with illness and complications during pregnancy and childbirth. Pregnant women who are anaemic are much more likely to die during childbirth than those women who are not and their infants are more likely to be born small for gestational age.

In Nepal, Kapilbastu is one of the districts where anaemia is highly prevalent. Hence, we have chosen 54 clusters (103 old-wards) within 9 pallikas of Kapilbastu for this study. The government of Nepal recommends minimum 4 ANC visits during pregnancy and consumption of 180 tablets of iron and folic acid starting from 20 week's gestation. However, compliance to these recommendations is low, increasing the risk of anemia during pregnancy. The situation has become worse since March 2020 with ongoing COVID-19 imposed travel restrictions and lockdowns, which have made health care less accessible. The health and nutrition status of pregnant women has deteriorated as women are afraid to visit health facilities to seek care.

Objective:

The VALID trial is designed to assess if providing antenatal virtual counselling on a tablet increases compliance to intake of the required dosage of IFA tablets, and improves dietary diversity and dietary practices, compared with women who have access to routine antenatal care (ANC) only (control).

This trial is being implemented in 9 pallikas of Kapilvastu district among 13 to 49 years old married pregnant women. Around 300 pregnant women will be enrolled and allocated to either of the 2 trial arms.

In the intervention arm, in addition to routine pregnancy care, 150 pregnant women will be provided with electronic tablets (like large Smart Phones) which are fitted with sim cards to receive virtual counselling sessions. A female member of HERD staff who is qualified as an ANM (a "nutrition assistant") will visit you

For peer review only - http://bmjopen.bmj.com/site/about/guidelines.xhtml

For peer review only

in your home to lend you a tablet to provide virtual counselling session. After this she will provide you with two virtual counselling sessions: one between 12 to 28 week's gestation and another two weeks after the first session. At each session she will discuss with you and your family members about your diet and health in pregnancy and give you advice based on what you are already eating / doing. She will encourage you to discuss and develop an action plan to improve your diet, consume iron tablets and antenatal care to minimize the risk of anaemia during pregnancy.

In the control arm, 150 pregnant women will be encouraged to go for routine pregnancy related services provided by the government of Nepal that includes antenatal care and iron and folic acid tablets.

Who are we inviting to participate?

You can take part in this research if you are a pregnant woman or girl aged 13 to 49 years, less than 28 weeks of gestation, planning to live in ,and/or seek services from the health facilities in, the study area and are able to respond to the survey questions. Pregnant women will be enrolled with the help of the health workers or female community health volunteers in these communities. If you are interested to take part in the trial, we will give you this trial information sheet to read or have others read to you. Once you have listened-to (or read) and understood the information sheet we will ask you to give us sign the consent form agreeing to participating in the trial. The risk or danger of participating in the trial is minimal. Participation in this trial is voluntary. If for any reason you want to withdraw your consent you can do so at any time.

What will happen if you agree to take part in this study?

If you decide to take part and give your consent (by signing or thumb-printing a consent form), first I will give you a unique identification number. On a tablet, I will record some personal details about you such as your age, education, household details, obstetric history, eating habits, iron supplements, physical activity and antenatal care. After responding to these questions, you will be assigned randomly to one of the two arms of the trial (intervention or control). It will take around 90 minutes to answer the questions and to randomly assign you to the trial arm.

Regardless of which arm you are assigned to, I will visit you again in about one month to enquire about your eating habits, iron and folic acid consumptions, antenatal care during pregnancy. This will take additional 90 minutes or so.

But if you are assigned to the intervention arm (virtual counselling) we will provide you a tablet with sim card to use during the intervention. You will receive two virtual counselling sessions 2 to 3 weeks apart and each session will last around 90 to 120 minutes. During these sessions we will discuss with you and your family members the importance of eating iron rich food during pregnancy, identify problems or barriers to eating iron rich food, identify solutions and develop action plans to implement them.

Therefore, if you are assigned to the intervention group, overall, you will need to provide around 5 hours' time, whereas if you fall in the control group (routine care) group you will need to provide 3 hours' minutes to the trial.

At the end of data collection, we will transfer 200 rupees' worth of mobile top up to your cell phone number or to a number of a person of your choice if you do not have your own cell phone.

Are there any risks if you participate?

We do not think that any harm will come to you, but it is possible that you might find sharing information about your pregnancy uncomfortable or upsetting. You don't have to continue to take part if you don't feel like it. If you would like to talk to someone about the feelings generated by the questions, please contact a member of HERD staff.

For peer review only - http://bmjopen.bmj.com/site/about/guidelines.xhtml

Are there any benefits if you participate?

There is no direct benefit of participating in this study, however you might enjoy interacting with the counsellor on the tablet. If found effective and feasible, the results of this trial will provide evidence that counselling can be provided virtually to improve health and nutrition of pregnant women from excluded communities in Kapilbastu and rest of Nepal.

Will my taking part in this project be kept confidential?

All the information that we collect about you during the course of the research will be kept strictly confidential. Only researchers directly associated with this project, who are involved in finding you to interview you, will have access to your name and address. All other researchers who look at the information you share with us will not be able to identify you as your name and address will be removed. You will be allocated a unique number, which will be used as a code to identify you instead of your name. You will not be able to be identified in any ensuing reports or publications.

If you consent to take part in this study, the records obtained while you are in this study (age, ethnicity, religion, education and so on) will remain strictly confidential at all times. The information will be held securely on either paper or electronically at HERD International and in University College London in the UK under the provisions the local Data Protection laws. Your name will not be passed to anyone else outside the research team who is not involved in the trial. Your records will be available to people authorized to work on the trial and those responsible for ensuring that the study is carried out correctly. By signing the consent form you agree to this access for the current study. Further research might involve other researchers using the information you give us, but without your name attached to it. Alternatively, we or other researchers might seek to find you in the future to undertake further research with you.

If you withdraw consent from further study, unless you object, your data and samples will remain on file and will be included in the final study analysis.

Ethical approval

This study has been approved by the Nepal Health Research Council Approval ID number 570/2021.

Agreeing to take part

Your participation is voluntary. If you don't want to take part, you can refuse without giving a reason. If you decide to take part in the study, you will be given this information sheet to keep and be asked to sign or thumb print the consent. If you agree to participate and then change your mind at any time, please tell us and we will stop visiting you or telephoning you. We will take a photo of the consent form with your signature which will be filed in your records. You can have more time to think this over if you are at all unsure.

Data safety procedure

All participants will need to provide information two times during the trial, once at enrolment ≤ 28 weeks' gestation and another at 16 to 33 weeks' gestation. Depending on the COVID-19 situation, the information will be collected either over the phone or in person. Data assistants will assess eligibility to confirm pregnancy and gestational age, and take written consent before collecting data. The collected data will be kept confidential, stored in protected servers that can be assessed by authorised personnel only.

Expected outcome of the Trial"

This study is expected to help understand how the virtual nutrition counselling intervention can improve consumption of iron and-folic acid, intake and absorption of iron rich food and antenatal visits among

For peer review only

pregnant women. As a result of this trial, pregnant women will be encouraged to go for antenatal check-up, deliver babies in health facilities and contribute in reducing the burden of anaemia during pregnancy.

Use of the trial results:

The evidence generated by the trial can guide government and other stakeholders in development of plans and programs for maternal nutrition and to reduce the burden of anaemia during pregnancy.

Contact for further information

You are encouraged to ask any questions you wish, before, during or after getting involved. If you have any questions about the study, please speak to the HERD International researchers who visit you, who will be able to provide you with up to date information about the trial. If you require any further information or have any concerns while taking part in the study you can contact:

Trial Manager, HERD International, Prasuti Griha Marg, Thapathali, Kathmandu. Tel 01-4238045

Or

Dr Naomi Saville, Senior Research Associate, University College London Institute for Global Health and Technical advisor to HERD, Kathmandu Nepal. Tel: 01-4238045

HERD International District Office, Taulihawa, Kapilbastu. Tel: number: 076-590090

Thank you for listening to / reading this information sheet and for considering whether to take part in this research study.

For peer review only

Patient Identification Number for this trial:

CONSENT FORM

Title of Study: Virtual Antenatal Intervention for improved Diet and Iron intake (VALID) Trial

Please complete this form after you have read the Information Sheet and/or listened to an explanation about the research.

Name and Contact Details of the Researcher(s):

DR SUSHIL BARAL 9851068940 and DR NAOMI SAVILLE 9851017232

For peer review only

This study has been approved by the Nepal Health Research Council Approval ID number 570/2021.

Thank you for considering taking part in this research. The researcher who has come to your home or who you meet with over the telephone must explain the project to you before you agree to take part. If you have any questions arising from the Information Sheet or explanation already given to you, please ask the researcher before you decide whether to join the study. You will be given a copy of this Consent Form to keep and refer to at any time.

I confirm that I understand that by ticking/initialling each box below I am consenting to this element of the study. I understand that it will be assumed that unticked boxes means that I DO NOT consent to that part of the study. I understand that by not giving consent for any one element that I may be deemed ineligible for the study.

In case of the married girl of age 14 to 19 running years (13 to 18 completed years), take the consent from at least one of her guardians as well. This means filling the tick box columns with the participant **and** ***her guardian and getting the signature or thumb print of both of them.***

	Pregnant woman's Tick Box	Guardian's Tick box
I confirm that I have read the Information Sheet for the above study (or it has been read to me in an appropriate language) and I understood its content.		
I have had an opportunity to consider the information and what will be expected of me and to ask questions which have been answered to my satisfaction.		
I understand that my participation in this research is completely voluntary. I understand that I am free to withdraw from the research at any time without any repercussions. **I understand that I can halt an interview at any point if I feel uncomfortable.**		
I understand the potential risks of participating and the support that will be available to me should I become distressed during the course of the research.		

I understand that I will not benefit financially from this study or from any possible outcome it may result in in the future.		
I understand that my personal information *(including ethnicity, age, socio-economic and educational status, past medical and obstetric history)* will be used when analysing the data, but not my name.		
I understand that all personal information will remain confidential and that all efforts will be made to ensure I cannot be identified by anyone, except those who are involved in meeting me to ask questions.		
I understand that my data gathered in this study will be securely stored anonymously (without my name and address) and that it will not be possible to identify me in any publications.		
I understand that the data will not be made available to any commercial organisations but are solely the responsibility of the researcher(s) undertaking this study.		
I agree that my anonymised research data (that has my name and address removed) may be used by others for future research. I understand that no one will be able to identify you me if/when this these data are shared.		
I would be happy for the data I provide, including my name and address, to be archived in the UCL Data Safe Haven to be kept there, and other secure locations in Nepal and UK, in case researchers need to find me again in the future. I understand that these personally identifiable data will not be available to anyone except authorised researchers who need my name and address to find me again in the future.		
I understand that when I consent to have my photos taken while engaging in the research activities during the research period, that these could be published on HERD international's or UCL's website or other publications.		
I would be happy to be contacted in future by HERD International, UCL and/or other researchers who would like to invite me to participate in follow-up studies to this project (for example if someone came to measure me or my child after this study ended) **.**		
I have informed the researcher of any other research in which I am currently involved or have been involved in during the past 12 months.		
**I consent to participate in this study**		

Phone no for future contact:

For peer review only

Signing this document means that you voluntarily agree to participate in this research after understanding the information provided to you.

(In case the married participant's age is between 14 and 19 running years (13 -18 completed years)

____________________	____________________	____________________
Name of guardian	Date	Signature

OR Guardian's Finger Prints

Left	Right

____________________	____________________	____________________
Name of participant	Date	Signature

OR Participant's Finger Prints

Left	Right<br>Left

____________________	____________________	____________________
Researcher (Data Assistant)	Date	Signature

For peer review only

STANDARD PROTOCOL ITEMS: RECOMMENDATIONS FOR INTERVENTIONAL TRIALS

SPIRIT 2013 Checklist: Recommended items to address in a clinical trial protocol and related documents*

Section/item	Item No	Description	Addressed on page number
**Administrative information**			
Title	1	Descriptive title identifying the study design, population, interventions, and, if applicable, trial acronym	1
Trial registration	2a	Trial identifier and registry name. If not yet registered, name of intended registry	1
	2b	All items from the World Health Organization Trial Registration Data Set	1
Protocol version	3	Date and version identifier	1
Funding	4	Sources and types of financial, material, and other support	1
Roles and responsibilities	5a	Names, affiliations, and roles of protocol contributors	1
	5b	Name and contact information for the trial sponsor	2
	5c	Role of study sponsor and funders, if any, in study design; collection, management, analysis, and interpretation of data; writing of the report; and the decision to submit the report for publication, including whether they will have ultimate authority over any of these activities	2
	5d	Composition, roles, and responsibilities of the coordinating centre, steering committee, endpoint adjudication committee, data management team, and other individuals or groups overseeing the trial, if applicable (see Item 21a for data monitoring committee)	2

For peer review only

Introduction

Background and rationale	6a	Description of research question and justification for undertaking the trial, including summary of relevant studies (published and unpublished) examining benefits and harms for each intervention	5
	6b	Explanation for choice of comparators	7
Objectives	7	Specific objectives or hypotheses	6
Trial design	8	Description of trial design including type of trial (eg, parallel group, crossover, factorial, single group), allocation ratio, and framework (eg, superiority, equivalence, noninferiority, exploratory)	7

Methods: Participants, interventions, and outcomes

Study setting	9	Description of study settings (eg, community clinic, academic hospital) and list of countries where data will be collected. Reference to where list of study sites can be obtained	7
Eligibility criteria	10	Inclusion and exclusion criteria for participants. If applicable, eligibility criteria for study centres and individuals who will perform the interventions (eg, surgeons, psychotherapists)	7
Interventions	11a	Interventions for each group with sufficient detail to allow replication, including how and when they will be administered	8
	11b	Criteria for discontinuing or modifying allocated interventions for a given trial participant (eg, drug dose change in response to harms, participant request, or improving/worsening disease)	14
	11c	Strategies to improve adherence to intervention protocols, and any procedures for monitoring adherence (eg, drug tablet return, laboratory tests)	9
	11d	Relevant concomitant care and interventions that are permitted or prohibited during the trial	9
Outcomes	12	Primary, secondary, and other outcomes, including the specific measurement variable (eg, systolic blood pressure), analysis metric (eg, change from baseline, final value, time to event), method of aggregation (eg, median, proportion), and time point for each outcome. Explanation of the clinical relevance of chosen efficacy and harm outcomes is strongly recommended	9 Tables 1 & 2
Participant timeline	13	Time schedule of enrolment, interventions (including any run-ins and washouts), assessments, and visits for participants. A schematic diagram is highly recommended (see Figure)	17

Sample size	14	Estimated number of participants needed to achieve study objectives and how it was determined, including clinical and statistical assumptions supporting any sample size calculations	11
Recruitment	15	Strategies for achieving adequate participant enrolment to reach target sample size	11-12
**Methods: Assignment of interventions (for controlled trials)**			
Allocation:			
Sequence generation	16a	Method of generating the allocation sequence (eg, computer-generated random numbers), and list of any factors for stratification. To reduce predictability of a random sequence, details of any planned restriction (eg, blocking) should be provided in a separate document that is unavailable to those who enrol participants or assign interventions	8
Allocation concealment mechanism	16b	Mechanism of implementing the allocation sequence (eg, central telephone; sequentially numbered, opaque, sealed envelopes), describing any steps to conceal the sequence until interventions are assigned	8
Implementation	16c	Who will generate the allocation sequence, who will enrol participants, and who will assign participants to interventions	8
Blinding (masking)	17a	Who will be blinded after assignment to interventions (eg, trial participants, care providers, outcome assessors, data analysts), and how	8
	17b	If blinded, circumstances under which unblinding is permissible, and procedure for revealing a participant's allocated intervention during the trial	8
**Methods: Data collection, management, and analysis**			
Data collection methods	18a	Plans for assessment and collection of outcome, baseline, and other trial data, including any related processes to promote data quality (eg, duplicate measurements, training of assessors) and a description of study instruments (eg, questionnaires, laboratory tests) along with their reliability and validity, if known. Reference to where data collection forms can be found, if not in the protocol	11
	18b	Plans to promote participant retention and complete follow-up, including list of any outcome data to be collected for participants who discontinue or deviate from intervention protocols	13

Data management	19	Plans for data entry, coding, security, and storage, including any related processes to promote data quality (eg, double data entry; range checks for data values). Reference to where details of data management procedures can be found, if not in the protocol	13
Statistical methods	20a	Statistical methods for analysing primary and secondary outcomes. Reference to where other details of the statistical analysis plan can be found, if not in the protocol	12
	20b	Methods for any additional analyses (eg, subgroup and adjusted analyses)	12
	20c	Definition of analysis population relating to protocol non-adherence (eg, as randomised analysis), and any statistical methods to handle missing data (eg, multiple imputation)	13
**Methods: Monitoring**			
Data monitoring	21a	Composition of data monitoring committee (DMC); summary of its role and reporting structure; statement of whether it is independent from the sponsor and competing interests; and reference to where further details about its charter can be found, if not in the protocol. Alternatively, an explanation of why a DMC is not needed	2, 12
	21b	Description of any interim analyses and stopping guidelines, including who will have access to these interim results and make the final decision to terminate the trial	16
Harms	22	Plans for collecting, assessing, reporting, and managing solicited and spontaneously reported adverse events and other unintended effects of trial interventions or trial conduct	16
Auditing	23	Frequency and procedures for auditing trial conduct, if any, and whether the process will be independent from investigators and the sponsor	16
**Ethics and dissemination**			
Research ethics approval	24	Plans for seeking research ethics committee/institutional review board (REC/IRB) approval	16
Protocol amendments	25	Plans for communicating important protocol modifications (eg, changes to eligibility criteria, outcomes, analyses) to relevant parties (eg, investigators, REC/IRBs, trial participants, trial registries, journals, regulators)	17

Consent or assent	26a	Who will obtain informed consent or assent from potential trial participants or authorised surrogates, and how (see Item 32)	8, 16-17
	26b	Additional consent provisions for collection and use of participant data and biological specimens in ancillary studies, if applicable	Not applicable
Confidentiality	27	How personal information about potential and enrolled participants will be collected, shared, and maintained in order to protect confidentiality before, during, and after the trial	13, 17
Declaration of interests	28	Financial and other competing interests for principal investigators for the overall trial and each study site	2
Access to data	29	Statement of who will have access to the final trial dataset, and disclosure of contractual agreements that limit such access for investigators	13
Ancillary and post-trial care	30	Provisions, if any, for ancillary and post-trial care, and for compensation to those who suffer harm from trial participation	Not applicable
Dissemination policy	31a	Plans for investigators and sponsor to communicate trial results to participants, healthcare professionals, the public, and other relevant groups (eg, via publication, reporting in results databases, or other data sharing arrangements), including any publication restrictions	17
	31b	Authorship eligibility guidelines and any intended use of professional writers	18
	31c	Plans, if any, for granting public access to the full protocol, participant-level dataset, and statistical code	17
**Appendices**			
Informed consent materials	32	Model consent form and other related documentation given to participants and authorised surrogates	Supplemental Annex 1
Biological specimens	33	Plans for collection, laboratory evaluation, and storage of biological specimens for genetic or molecular analysis in the current trial and for future use in ancillary studies, if applicable	Not Applicable

*It is strongly recommended that this checklist be read in conjunction with the SPIRIT 2013 Explanation & Elaboration for important clarification on the items. Amendments to the protocol should be tracked and dated. The SPIRIT checklist is copyrighted by the SPIRIT Group under the Creative Commons "Attribution-NonCommercial-NoDerivs 3.0 Unported" license.

BMJ Open

A study protocol for a randomised controlled trial of a Virtual Antenatal Intervention for improved Diet and Iron intake in Kapilvastu district, Nepal: VALID

Journal:	*BMJ Open*
Manuscript ID	bmjopen-2022-064709.R1
Article Type:	Protocol
Date Submitted by the Author:	27-Jan-2023
Complete List of Authors:	Saville, Naomi; University College London, Institute for Global Health<br>Bhattarai, Sanju; HERD International<br>Harris-Fry, Helen; LSHTM<br>Giri, Santosh; HERD International<br>Manandhar, Shraddha; HERD International<br>Morrison, Joanna; University College London, Institute for Global Health<br>Copas, Andrew; University College London, Institute for Global Health<br>Thapaliya, Bibhu; HERD International<br>Arjyal, Abriti; HERD International<br>Haghparast-Bidgoli, Hassan; University College London, Institute for Global Health<br>Baral, Sushil; HERD International; HERD<br>Hillman, Sara; University College London, Institute for Women's Health
**Primary Subject Heading**:	Global health
Secondary Subject Heading:	Nutrition and metabolism
Keywords:	NUTRITION & DIETETICS, PUBLIC HEALTH, EPIDEMIOLOGY

SCHOLARONE™
Manuscripts

A study protocol for a randomised controlled trial of a Virtual Antenatal Intervention for improved Diet and Iron intake in Kapilvastu district, Nepal: VALID

Naomi M. Saville[1]*, Sanju Bhattarai[2], Helen Harris-Fry[3], Santosh Giri[2], Shraddha Manandhar[2], Joanna Morrison[1], Andrew Copas[1], Bibhu Thapaliya[2], Abriti Arjyal[2], Hassan Haghparast-Bidgoli[1], Sushil Baral[2,4], Sara Hillman[5]

Author affiliations:

1. Institute for Global Health, University College London (UCL), London, UK
2. HERD International, Kathmandu, Nepal
3. London School of Hygiene & Tropical Medicine (LSHTM), London, UK
4. Health Research and Social Development Forum (HERD), Kathmandu, Nepal
5. Institute for Women's Health, University College London (UCL), London, UK

* Corresponding author: Naomi Saville, email: n.saville@ucl.ac.uk

Trial registration

Trial Registry name: ISRCTN; Registration number: ISRCTN 17842200; Date of registration: 13 Jan 2022. URL: https://doi.org/10.1186/ISRCTN17842200

World Health Organization Trial Registration Data Set

ISRCTN collects all items from the World Health Organization Trial Registration Data Set.

Protocol version

Version number 1.0, 1 May 2022

Funding

This work was supported by UK Medical Research Council (MRC)/ Newton Fund, grant number MR/R020485/1. The funding body has no role in the study design, collection, analysis, or interpretation of data, in the writing of the manuscript, or the decision to submit the report for publication.

Trial sponsor

For peer review only

Trial sponsor is Health Research and Social Development Forum (HERD) Nepal, PO Box 24133, Prasuti Griha Marg, Thapathali, Kathmandu, Nepal.

The trial sponsor, HERD Nepal under the leadership of Sushil C. Baral, working with the trial coordinating centre, University College London Institute for Women's Health, takes responsibility for study design; collection, management, analysis, and interpretation of data; writing of the report; and the decision to submit the report for publication. Authority over these activities is shared between HERD and UCL.

Composition of the coordinating centre, Trial Steering Committee and Data Monitoring Committee

The Trial Management Group (TMG) comprises representatives from HERD International (SCB, SB, SM and SG), the PI / local PI, process evaluation adviser and trial statistician from UCL (SH, NS, JM, AC) and dietary specialist (HHF). The TMG meet weekly.

The Trial Steering Committee (TSC) comprises independent specialists on maternal nutrition, anaemia, haematology, conduct of trials, and/or maternal nutrition in Nepal from the UK, India, Nepal, and Canada. The TSC provides oversight and advice through its independent Chair in alignment with the TSC charter. The TSC met by teleconference prior to the start of enrolment to approve the protocol and will meet after data collection has completed. Between meetings any trial issues arising are dealt with by email.

The Data Monitoring Committee (DMC) comprises experts on global health trials, statistics, obstetric haematology, maternal nutrition, micronutrient status and research ethics in Nepal, with two members from Nepal and the remaining three from the US, UK, and India. While interim safety data cannot be reviewed by the DMC due to the short trial duration, the DMC will review and approve the Trial Analysis Plan.

Declaration of interests

The authors declare that they have no competing interests.

For peer review only

Abstract

Introduction: Despite evidence that iron and folic acid (IFA) supplements can improve anaemia in pregnant women, uptake in Nepal is suboptimal. We hypothesised that providing virtual counselling twice in mid-pregnancy, would increase compliance to IFA tablets during the COVID-19 pandemic compared with ANC alone.

Methods and Analysis: This non-blinded individually randomised controlled trial in the plains of Nepal has two study arms: 1) Control: routine antenatal care (ANC); 2) 'Virtual' antenatal counselling plus routine ANC. Pregnant women are eligible to enrol if they are married, aged 13-49 years, able to respond to questions, 12-28 weeks' gestation, and plan to reside in Nepal for the next 5 weeks. The intervention comprises two virtual counselling sessions facilitated by auxiliary nurse midwives at least 2 weeks apart in mid-pregnancy. Virtual counselling uses a dialogical problem-solving approach with pregnant women and their families. We randomised 150 pregnant women to each arm, stratifying by primi-/multi-gravida and IFA consumption at baseline, providing 80% power to detect a 15% absolute difference in primary outcome assuming 67% prevalence in control arm and 10% loss-to-follow-up. Outcomes are measured 49-70 days after enrolment, or up to delivery otherwise. Primary outcome: consumption of IFA on at least 80% of the previous 14 days. Secondary outcomes: dietary diversity, consumption of intervention-promoted foods, practicing ways to enhance bioavailability, and knowledge of iron-rich foods. Our mixed methods process evaluation explores acceptability, fidelity, feasibility, coverage (equity and reach), sustainability, and pathways to impact. We estimate costs and cost-effectiveness of the intervention from a provider perspective. Primary analysis is by intention-to-treat, using logistic regression.

Ethics and Dissemination: We obtained ethical approval from Nepal Health Research Council (**570/2021**) and UCL ethics committee (**14301/001**). We will disseminate findings in peer reviewed journal articles and by engaging policymakers in Nepal.

Trial registration number: ISRCTN 17842200

For peer review only

Strengths and limitations of this study

1. The virtual counselling intervention under test with pregnant women is novel and tailored to a pandemic situation where opportunities for face-to-face antenatal counselling may be limited.
2. The intervention design to engage pregnant women and their family members in a problem-solving interactive discussion is based upon formative research on the barriers to uptake of iron and folic acid supplements and antenatal care and our process evaluation analysis of context will help us to understand the external validity of the results.
3. Carefully designed electronic data collection forms enable thorough follow-up of participants, drawing on their information at recruitment where needed to validate outcome data.
4. Necessary adaptation to the COVID-19 context led to delays in starting the trial, constraining the time frame within the funding period. This means that the time frame to enrol each woman, provide her with two sessions of virtual counselling, and capture her outcomes is shorter than may have been ideal.
5. Our study carries some risk of bias. As with all non-blinded trials we could not eliminate the potential for selection bias. Our self-reported outcomes also carry risk of social desirability and recall bias. The electronic questionnaires included internal consistency and range checks to validate responses for each outcome, and to minimize recall bias on the primary outcome (IFA consumption) the interviewers triangulated responses using a series of probes which asked how many days the participant consumed IFA, and how many days were missed, before confirming the final number with the respondent.

For peer review only

INTRODUCTION

Anaemia in pregnancy is a complex public health problem that leads to severe adverse maternal and child health outcomes, including maternal morbidity and mortality [1-4] and impaired infant growth [5]. No country in South Asia is on course to meet the World Health Assembly target of a 50% reduction in anaemia by 2025 [6] and estimates from Nepal's 2016 Demographic and Health Survey indicate that 46% of pregnant women were anaemic (haemoglobin concentration < 11 g/dL) [7] – almost no change from the 2011 estimate of 48% [8].

Despite the lack of progress in reducing anaemia in pregnancy, and its multifactorial aetiology [9-11], there are several effective interventions that could help. Systematic reviews of randomised controlled trials show that consumption of IFA supplements and deworming with anti-helminthics in pregnancy can both lower the risk of maternal anaemia – by 70% and 15% respectively [12, 13]. There are additional health benefits of IFA supplementation beyond anaemia, including increased birth weight [14-16], child survival [17] and development [18].

Provision of free-of-cost IFA and anti-helminthics have been scaled up in Nepal from 20 weeks' gestation as part of routine care in Nepal. The challenge lies in supporting women to access and use these evidence-based interventions [19]. Current access to recommended antenatal interventions is improving but remains sub-optimal. In 2016, 41% of women took iron tablets on at least 180 days of pregnancy, 69% took anti-helminthics, and the same proportion had four antenatal clinic (ANC) visits [8].

Although these aspects of ANC have improved markedly between 2011 and 2016 [7] [8], the COVID-19 pandemic threatens to compromise this progress [20]. The pandemic has seen a sharp rise in neonatal and maternal mortality, along with declines in institutional deliveries and quality of care [21, 22]. In Kapilvastu district of Nepal during the early phase of the pandemic in 2019/20, Maternal Mortality Ratio increased from 0.0 to 14.4 per 100,000 live births. Between 2018 and 2020, having at least 1 ANC visit decreased from 84% to 74% and having four ANC visits decreased from 63% to 52%, which would likely decrease uptake and adherence to IFA [23-26]. Repeated lockdowns, concerns about going to a health facility, discrimination against frontline workers (Female Community Health Volunteers, FCHVs) and loss of livelihoods could all pose barriers to women's access to ANC and IFA [22].

Nutrition education and counselling interventions have had variable effects on maternal anaemia [27]. In Nepal, the complexity of factors limiting access to adequate nutrition and IFA require gender-sensitive, tailored, participatory counselling methods, rather than a 'one-size-fits-all' didactic educational approach [28-30]. A recent trial of a social norms intervention in India

For peer review only - http://bmjopen.bmj.com/site/about/guidelines.xhtml

found large effects on consumption of iron-folic acid consumption, suggesting that such an approach holds promise [31].

A question remains as to whether and how these types of interventions can be adapted to reach the most geographically and socially isolated, who have least access to antenatal and social support. Virtual interventions may offer a solution, with almost all (93%) of Nepalese households now owning a mobile phone [7]. Systematic reviews have shown that 'eHealth' and 'mHealth' can improve a range of nutrition outcomes, including physical activity and healthy eating behaviour [32], but research has been concentrated in high-income contexts. Use of telemedicine for maternal and newborn care during the pandemic has been beneficial, but difficulties in reaching high-risk groups and maintaining quality of maternity services remain in low-income countries [33]. In rural Nepal, virtual counselling has the potential to address the shortage of healthcare professionals, and transportation and travel time barriers to accessing health care [34].

STUDY AIMS AND HYPOTHESES

In our study, designed in accordance with the SPIRIT guidelines [35], we test whether a virtual counselling intervention offered to pregnant women living in Kapilvastu could offer a feasible, affordable, scalable, and equitable solution to increase consumption of iron-folic acid supplements.

The primary objective of the Virtual Antenatal Intervention for Improved Diet and iron intake (VALID) trial is to assess whether providing pregnant women with an antenatal virtual counselling intervention, in addition to usual government services, increases women's compliance to IFA supplementation, compared with pregnant women who only had access to usual government services. Compliance is defined as: IFA consumed on 12 out of the last 14 days before the endline interview (which is 49-70 days after baseline). We hypothesised that the antenatal virtual counselling intervention would increase pregnant women's compliance to IFA tablets.

Secondary objectives are as follows:

1. To assess whether providing an antenatal virtual counselling intervention to pregnant women improves

- Dietary diversity,
- Timing and number of antenatal visits (ANC),
- Consumption of recommended iron-rich foods
- Practice of iron absorption-enhancing behaviours
- Knowledge of iron-rich foods and anaemia risks in pregnancy.

For peer review only - http://bmjopen.bmj.com/site/about/guidelines.xhtml

- Knowledge of COVID symptoms, prevention, and vulnerable population groups

2. To evaluate the feasibility, acceptability, equity, cost, cost-effectiveness and reach of the antenatal virtual counselling intervention (using an Implementation Science approach)

METHODS AND ANALYSIS

VALID trial design and setting

This is a non-blinded two-arm individually randomised controlled trial, with an allocation ratio of 1:1, conducted in Kapilvastu district, Lumbini Province of Nepal.

Trial arms are:

1. Control: access to routine antenatal care (ANC).
2. 'Virtual counselling': comprising of two interactive virtual counselling sessions provided to pregnant women in mid pregnancy, using 'zoom' on a tablet, in addition to routine ANC.

The trial is being implemented in 54 clusters in Kapilvastu district, Lumbini Province of Nepal, in the Western Terai (plains) of Nepal, bordering Uttar Pradesh state of India. The district population is 569,844 and literacy rates of women and men are 45% and 65% respectively [36]. Hinduism is the main religion, followed by Islam and the predominant ethnicity is Madhesi, many of whom speak Awadhi language. Anaemia prevalence is high (45%) and IFA in pregnancy is sub-optimal: 43% took at least the recommended dose of 180 IFA tablets, 33% took 60-179 tablets, and 24% took <60 tablets [7].

Eligibility

Study clusters (in Figure 1) which rural areas of southern Kapilvastu district that do not adjoin the main East-West highway that traverses Nepal; with no major market; 1100 -3199 projected population; surrounded with a buffer zone of non-study clusters; and >50% Madhesi (plains ethnicity) as per the pre-trial census.

Figure 1. Map of study clusters

Pregnant women are eligible for a baseline survey if they are aged 13-49 years, able to respond to questions, reside in a study cluster, and consent to participate. Additional inclusion criteria for enrolment in the trial include: 12 to 28 weeks' gestation (estimated from recall of last menstrual period or expected date of delivery given by a health worker), does not plan to leave the country

For peer review only

during the 5 weeks since enrolment, and no other pregnant woman in her household already enrolled in the trial.

To enrol participants, interviewers identify pregnant women in their study areas with help from FCHVs. They confirm eligibility, take written consent, and then phone the office to receive the random allocation.

Randomisation

A stratified block randomisation process is used to allocate pregnant women enrolled into the trial intervention or control arm. To balance the trial arms by two strong pre-identified determinants of IFA consumption, parity and baseline IFA consumption status, randomisation is done within each of four strata: (IFA yes/1st pregnancy); (IFA Yes/ not 1st pregnancy); (IFA no/1st pregnancy) and (IFA no/ not 1st pregnancy). For each stratum a separate allocation sequence is prepared. To make the sequence unpredictable, random permutation of the allocations within blocks is conducted and block sizes vary randomly between sizes 8, 6 and 4 using “blockrand” package in R programming software. Then, sequential allocations are sealed into sequentially numbered opaque envelopes by the project coordinator in Kathmandu, transported to the field office, stored in a locked cabinet, and opened by the monitoring and evaluation manager when the enumerators call to find out a pregnant woman’s allocation at the end of the baseline interview. After assignment of pregnant women to study arms, blinding of trial staff and participants is impossible, since interviewers distribute of tablet devices to pregnant women in the intervention arm.

For peer review only

Intervention

After enrolment, interviewers call pregnant women in the intervention arm to schedule appointments to deliver the tablets and orientate women on how to use the tablets. Tracking of tablet delivery is made by entering this information onto CommCare. Each tablet holds a sim card and has a data package which is topped up as necessary.

Enrolled women are assigned to one of ten counsellors, who are recruited and trained auxiliary nurse midwives. Counsellors call pregnant women, using case lists provided in the CommCare app on their tablets, to schedule their tailored virtual counselling sessions via Zoom. Pregnant women are offered their first counselling session after enrolment, at 12 to 28 weeks’ gestation, and the second counselling session is around two weeks later, at 14 to 31 weeks’ gestation. A diagram summarising the virtual counselling intervention is shown in Figure 2.

The counselling aims to support women and their families to take actions to change dietary practices, take IFA and deworming tablets, and access ANC. Counselling is conducted with pregnant women and their families to support them to think critically about the causes of anaemia

For peer review only - http://bmjopen.bmj.com/site/about/guidelines.xhtml

in pregnancy in their household and community, using stories and inductive questioning to trigger dialogue and reflection [37]. Stories directly address issues identified from formative research [38]. At each session, pregnant women (and their families if they attend) are engaged in a cycle of action and reflection whereby they discuss common issues that affect women in pregnancy and examples of actions for improving intake of IFA and deworming tablets, accessing ANC, and dietary practices. At the end of the first counselling session, the pregnant women and families make specific action plans to address the issues that are relevant for their family. In the second counselling session these action plans are reviewed and further discussed to support women and their families to address the issues and a second action plan is made. The tablets are collected back by interviewers after completing two counselling sessions.

Figure 2: Virtual counselling intervention model

The auxiliary nurse midwives received in-depth training on how to engage families in problem solving, the dialogical approach, and how to use a reference manual with examples of actions that pregnant women and their families could take. The training also provided counsellors with knowledge on anaemia consequences and prevention, and logistical training on how to use the tablets and record their progress.

Adherence to intervention protocols is ensured by monitoring the counselling (by supervisors joining remotely), by video-recording sessions, and by requiring counsellors to provide copies of action plans and to fill paper and electronic forms about each counselling session. Counsellors manage workload by prioritizing women approaching 28 weeks' gestation and filling a "phone call log" in CommCare to record outcome of each call.

Participants are free to seek concomitant care during pregnancy irrespective of their trial allocation.

Outcomes

The primary outcome of the trial is the proportion of pregnant women consuming IFA on at least 80% of the previous 2 weeks (i.e., on >=12 out of 14 days recalled). Other outcomes are given in Table 1.

Table 1. VALID outcome measures

For peer review only

**VALID TRIAL OUTCOMES**	**Recall period**	**Definition**	**Variable type**	**Effect measure to compare arms / summary statistic**
**PRIMARY OUTCOME**				
Proportion of pregnant women complying with recommended iron and folic acid tablet (IFA) intake	14 days	IFA consumed on 12 or more days out of the previous 14 days (i.e. on at least 80% of days)	Binary	Difference in Proportion
**SECONDARY OUTCOMES**				
Dietary diversity	24 hours	Count of the number of food groups consumed in the previous 24 hours preceding the endline interview, assessed using the list-based method, out of 10 food groups, as defined by (ref)	Count (0 to 10)	Difference in Mean
Consumption of intervention-promoted foods	24 hours	Any consumption of: green leafy vegetables, meat or fish	Binary	Difference in Proportion
Practicing one or more to enhance bioavailability	7 days	Recalled one or more of the following: using lemon or other vitamin C-rich foods with meals, eating sprouted grains or pulses, avoiding tea/coffee 1 hr either side of meals, or spreading meat-eating over two eating occasions rather than one.	Binary	Difference in Proportion
Knowledge of iron-rich foods	N/A	Count of iron-rich food groups correctly recalled	Count (0 to 9)	Difference in Mean
ANC visits	Baseline to endline	Count of Antenatal (ANC) visits between enrolment and endline interview	Count (0 to 6)	Difference in Mean
**EXPLORATORY OUCOMES**				
Understanding of why blood tests are taken at antenatal check-ups.	N/A	Proportion of those women who had a blood test at ANC who could correctly explain one or more reason for having a blood test.	Binary	Difference in Proportion
Knowledge of COVID-19 symptoms, precautions and vulnerability	N/A	Proportion of women who could correctly identify at least one vulnerable group, at least 3 COVID	Binary	Difference in Proportion

For peer review only

		control/prevention measures and at least 3 COVID symptoms		
ANC visits at the right time for her gestational age	Pregnancy to date	Whether the woman had her ANC visits at 2, 4, 6, and 8 months or not		

Outcome measurement

Interviewers measure outcomes on trial participants at enrolment (12 to 28 weeks' gestation) and endline (ideally 49 to 70 days after baseline, otherwise up to delivery). All outcomes are recalled by trial participants during interviews and recorded in electronic questionnaires. Additionally, at baseline we collect the woman's age, parity, medical history, date of the last menstrual period, pregnancy symptoms/ problems, and other key socioeconomic and demographic information. The data collection tools are programmed onto mobile devices in Nepali and English on Android operating system tablets or mobile phones using the CommCare electronic data collection platform [39]. These have in-built jump-sequences and value limits to prevent entry of data outside plausible ranges. As interviewers are fluent Awadhi speakers but do not read in Awadhi, they conduct the interviews by reading the question in Nepali and wording it into Awadhi to the respondent. Standard Awadhi wording is agreed upon within the team. Interviewers follow standard procedures for 24-hour dietary recall measurements and IFA recall to ensure that measurements are accurate and inter-observer difference minimised.

The monitoring team meets monthly and Kathmandu-based team members provide support as needed. Interviewers log any problems with electronic forms and the data team make corrections reversibly in the data using Stata data cleaning 'do' files as required.

Sample size

Our target sample size is 300 pregnant women, 150 in each arm. With at least 270 followed up (90% follow-up rate in each arm), we have 80% power to detect a 15 percentage-point increase in IFA compliance (the proportion of women consuming IFA on at least 12 out 14 days preceding the endline survey) if the control arm compliance prevalence is 66-68% (or 16.7 percentage point difference if control arm compliance is only 50%).

To achieve the required sample size, interviewers communicate with the Female Community Health Volunteers (FCHVs) in the study area. In line with government norms, FCHVs are provided with an incentive per day spent identifying pregnant women for our trial. To increase the response rate in later pregnancy, we provide an incentive (200 Nepali rupees mobile phone top-up) to trial

For peer review only - http://bmjopen.bmj.com/site/about/guidelines.xhtml

participants after the endline interview. Target dates for enrolment and endline interviews, based on gestational age and date of enrolment, are displayed on customised lists in CommCare on interviewers' mobile devices.

Statistical analysis

The trial statistician (AC) and Nepal principal investigator (NS) will make a Statistical Analysis Plan, to detail the analysis strategies, covariate adjustment, and the approach to any missing data. We will present this to the Trial Steering Committee (TSC) and Data Monitoring Committee (DMC) for approval before analysis. Primary analysis will be replicated by a second analyst.

Analyses will involve graphically presenting the number of days of IFA consumption in last 14 days by arm (bar chart) at baseline and follow-up by trial arm. Primary Analysis will be by intention-to-treat using two-sided testing at the standard 5% significance level. Analysis of our binary primary outcome, 12+ days consumption of IFA in last 14 days at follow-up, is based on logistic regression adjusting for baseline consumption in the previous 14 days, gestational age at interview and parity (note the randomisation strata are based on parity and baseline consumption). Our 'fully adjusted' results (secondary approach) will include additional adjustment for wealth score constructed out of household assets, education status of pregnant woman and age.

We will follow a similar methodology for the analysis of secondary and exploratory outcomes (given in Table 1), and process indicators to be reported in a separate publication. We will use linear regression for continuous outcomes, logistic regression for binary outcomes, and negative binomial regression for 'count' outcomes such as number of ANC visit between baseline and endline. We will examine the distribution of continuous and count outcomes before analysis of intervention effectiveness.

We plan to also report the intervention effect stratified by consumption of IFA at baseline, and test for an interaction with intervention arm. Within the intervention arm we will report and test the association between the degree of intervention exposure (number of counselling sessions received) and IFA consumption at endline. Other subgroup analyses will compare the intervention effect on the primary outcome by socio-economic status (wealth quintile), education and exposure to ANC.

Whilst our main analyses are under the intention-to-treat principle, for the primary outcome we will also conduct a 'per protocol' analysis in which only women who received virtual counselling are compared to all participants in the control arm.

For peer review only

We expect very low missing data in our baseline indicators, but we anticipate some women will not have a primary outcome. We consider data as missing where the woman is unavailable, has moved away, or withdrew consent but we do not regard data as 'missing' if due to miscarriage. We are not planning imputation of the primary outcome since there is little information on which to base the imputation other than baseline IFA consumption, which we are including as a covariate in our regression models.

Data management

We use CommCare's case management system that enables interviewers and counsellors to follow up pregnant women for data collection and intervention and promotes participant retention. Interviewers and FCHVs hold paper lists of potential pregnant women prior to enrolment. Data collected on electronic forms are locally stored before they are synchronised (encrypted) to the CommCare cloud server.

The data manager in Kathmandu downloads the data daily from the CommCare cloud server onto their server, imports the data into Stata and runs 'do' files for data pseudonymising, labelling and recoding. The data are stored on password-protected, encrypted, secure server computers in lockable rooms at the HERD International Kathmandu office. We back up data from physical servers onto secure cloud servers daily, and onto external hardware-encrypted hard drives each week. Person-identifiable data are stored in separate encrypted files and are only used to generate follow-up lists for authorised field team members. All other data are pseudonymised. Study arm is encoded but not labelled. After data collection is completed, we will delete VALID data from laptops, tablets, and the CommCare server.

For qualitative data collected in Awadhi and Nepali languages, qualitative interviewers transcribe the data in Nepali. They then send audio recordings and transcriptions to the qualitative data officer who checks completeness, anonymises, and stores the data in a lockable cabinet.

Data cleaning is completed by identifying outliers and removing as required. HERD International follows international and NHRC policies for archiving of data in Nepal. We will make the pseudonymised dataset open access by using the UCL data sharing platform as per MRC guidelines. Any request for archived person-identifiable data will go through the trial management team in alignment with provisions in the consent forms.

No storable biological specimens are collected during this study.

Process evaluation

For peer review only

372 We are conducting a mixed methods process evaluation to describe the feasibility, acceptability,
373 equity, reach, and fidelity to plans, as guided by the theory of change [38] [40] in Figure 3.

374

375 **Figure 3: Theory of Change for the VALID intervention**

376

377 Quantitative process outcomes are listed in Table 2.

378

379 **Table 2. Quantitative process evaluation outcomes**

Domain	Measure	Data source
Acceptability	Proportion of pregnant women who:<br>1) were satisfied with virtual counselling session<br>2) consented to participate in the trial out of those eligible at baseline<br>3) had family member(s) attending the virtual counselling session<br>4) reported the tablet and video link is an acceptable way to receive counselling and advice during pregnancy	Endline interview
Effectiveness	5) received satisfactory support from family members<br>6) implemented the action plan<br>7) found the intervention useful<br>8) understood what was said during the virtual counselling session<br>9) needed support to use the tablet and video link<br>10) reported constant internet disruption for smooth conversation during virtual counselling session<br>11) felt confident in their nutrition knowledge and/or able to take actions to improve nutrition/health	Endline interview
Feasibility	12) completed 2 virtual counselling sessions<br>13) had direct access to a phone/device<br>14) were easily able to use the tablet and video link<br>Number of attempts to contact participant when intervention not possible	Endline Interview<br>Phone call log
Fidelity	Proportion of Counsellors adhering to the virtual counselling manual guidance	Observations of sessions
Coverage and Reach	Proportion of<br>• pregnant women enrolled out of those approached<br>• marginalised Muslim and Dalit castes women enrolled out of those approached<br>• primiparous women enrolled of those approached	Baseline interview

For peer review only - http://bmjopen.bmj.com/site/about/guidelines.xhtml

	• young (<18 years of age) enrolled of those approached	
Sustainability	Proportion of organizations (government and non-governmental) attending the dissemination workshop willing to implement the intervention	Dissemination workshop
Cost	Total intervention cost from program provider perspective<br>Total intervention costs by line items/inputs<br>Cost of Intervention per PW	Costing tools and time allocation sheets

We will use qualitative data (semi-structured interviews) to explore how the context may influence intervention effect [38]. We will analyse post-counselling forms and observe virtual counselling sessions to describe intervention implementation and fidelity to plans. Further, we will conduct focus group discussions with supervisors and counsellors to explore how context affects the intervention and analyse factors affecting the families' interaction during the counselling sessions. We will interview 20 pregnant women who received the intervention, purposively sampled based on the number of children they have, and their socio-economic status. We will also interview 15 family members of different pregnant women (husbands and mothers-in-law) who did and did not participate in the intervention to explore the factors affecting their participation and their perceptions of the intervention. To consider the sustainability of the intervention we will show a piloted virtual counselling session to health workers in two health facilities and discuss the feasibility and acceptability of using virtual counselling within the health system and community. In addition, we will interview 2 FCHVs to explore the factors affecting recruitment and enrolment of women and their observation on others' (especially participants') perceptions of the intervention.

Cost effectiveness

We will estimate cost and cost-effectiveness of the intervention from a provider perspective by estimating the costs of designing and implementing the virtual counselling (program costs) and costs to public health care providers. We collect program cost data from HERD international project accounts, staff time use surveys, and interviews with project staff. We estimate costs to public health care providers resulting from any increased demand for health services caused by the intervention, using ANC and IFA data from the endline interview and any available secondary data on unit costs for these services. If statistically significant results are found, we will calculate

For peer review only

For peer review only - http://bmjopen.bmj.com/site/about/guidelines.xhtml

incremental cost effectiveness of virtual counselling compared to routine care and estimate incremental cost-effectiveness ratios (ICERs) for the primary outcome (i.e., proportion of pregnant women complying with recommended IFA intake) and the secondary outcome Women's Dietary Diversity Score,. We will estimate ICER in terms of the cost per number of women that complied with recommended IFA intake and the cost per unit change in in dietary diversity. If possible, we will estimate Disability Adjusted Life Years (DALYs) based on the primary outcome and present the results in terms of cost per DALYs averted. A series of one-way sensitivity analyses will be conducted to assess impact of uncertainties on the cost-effectiveness results.

Trial monitoring and safety

We do not expect virtual counselling to have negative effects, although we have an established complaints procedure. Asking women to discuss their pregnancy may be difficult or embarrassing; participants can stop an interview or counselling at any time. Counsellors are trained to advise on how to mitigate minor IFA side-effects, such as constipation and indigestion, or conflict in the household from provision of tablets [2, 41]. If the counsellor or interviewer observes any pregnancy danger signs or the woman is feeling unwell at the time of interaction, they advise the woman to seek care from the nearest health facility, as per the government's standard treatment protocol [42].

The virtual counselling intervention is designed to limit the spread of COVID-19, but interviewers need to visit participants' home to provide the tablets. We removed measurements requiring physical contact (anthropometry and haemoglobin) from our protocol to enable interviewers to keep 2 metres from household members, except while demonstrating use of the tablet. Field staff are required to adhere to COVID-19 Infection Prevention and Control (IPC) protocols.

Counsellors and interviewers record adverse events (including maternal deaths or COVID-19 cases) and inform field managers who submit an adverse events form to the PIs. As the trial follow-up period is short, we will not have interim audits. We do not expect to end the counselling early (unless the COVID-19 pandemic renders activities impossible), and we do not envisage ancillary or post-trial care will be needed beyond routine ANC.

ETHICS AND DISSEMINATION

We have obtained ethical approval from Nepal Health Research Council (NHRC approval number: **570/2021**) with amendment approval on 9 March 2022, UCL ethics committee (Project ID number: **14301/001** with amendment approval on 6 May 2022). We will seek approval for any major

For peer review only - http://bmjopen.bmj.com/site/about/guidelines.xhtml

changes to the protocol from the Trial Steering Committee and ethics boards and amend the registry.

General consent was sought from municipality leaders, health system managers, and ward representatives. Interviewers seek household heads' oral consent to speak with pregnant women, then take written consent/assent from pregnant women. Additional consent is obtained from guardians if the participant is aged <18 years. After assessing eligibility, the interviewers take consent to enrol eligible pregnant women into the trial by signature or thumbprint. In subsequent visits to pregnant women, interviewers and counsellors take oral consent. Information sheets and consent forms in English are available in Supplementary Annex 1 (copies in Nepali and Awadhi are available on request).

Our consent forms ask permission to share the anonymised data with other researchers for secondary analyses and to revisit the participant for follow-up. The final data will be held by UCL and HERD International on secure servers. The analysis dataset and statistical code will be shared in a web annex to the main trial publication.

Patient and Public Involvement

We conducted qualitative formative research with reproductive age women, mothers-in-law, fathers and FCHVs to inform the design of the study, using findings to refine our research questions/priorities and shape the intervention [29]. We did not involve patients/public in the choice of outcomes but involved FCHVs and health workers in participant recruitment. When disseminating study results, we will engage trial participants and municipal leaders from the study clusters as well as national and provincial policy makers, government officials, academics, and other relevant stakeholders. We will prepare a policy brief in English and Nepali and distribute it at dissemination workshops.

We will report results to NHRC within one year of the end of the study, publish our results in peer-reviewed journals and present findings at national, regional, and international conferences where possible.

CURRENT TRIAL STATUS

The trial timeline is provided in Figure 4.

Figure 4. Schedule of enrolment, interventions, and assessments

For peer review only - http://bmjopen.bmj.com/site/about/guidelines.xhtml

471 Interviewers enrolled women from 14 January until 23 February 2022. Endline interviews began on 9 March 2022 and are due to be completed by 19 June 2022, depending on the availability of participants.

Supplemental Annex 1. Consent form and participant information sheet

Abbreviations

Antenatal care (ANC)
Demographic and Health Survey (DHS)
Data Monitoring Committee (DMC)
Female Community Health Volunteer (FCHV)
Health Research and Social Development Forum, Nepal (HERD)
International Standard Randomised Controlled Trial Number (ISRCTN)
Iron and Folic Acid supplement (IFA)
Medical Research Council (MRC)
Nepal Health Research Council (NHRC)
Novel SARS-CoV-2 Corona Virus (COVID-19)
Trial Management Group (TMG)
Trial Steering Committee (TSC)
University College London (UCL)

Acknowledgements

We thank Suprich Sapkota, Basudev Bhattarai, Avinash Bhurtel, Anjali Basnet, and Samata Yadav for their contributions in development of this protocol.

Contributor statement

Naomi Saville (NS) led the development of the protocol and the writing of the manuscript draft. Sanju Bhattarai (SB) gave oversight to field management all trial processes and drafted the process evaluation and randomisation processes. Santosh Giri (SG) oversaw data collection tools with NS. Joanna Morrison, Shraddha Manandhar, Abriti Arjyal and Bibhu Thapaliya designed the intervention and process evaluation. Andrew Copas, NS, Helen Harris-Fry and SG designed the trial methodology, sample size calculation, and data analysis plan, and outcome measurement. Hassan Haghparast-Bidgoli designed the economic evaluation. SB, NS, Sushil Chandra Baral and Sara

For peer review only

Hillman provided overall leadership. All authors provided inputs and read and approved the final paper.

Provenance and peer review

Not commissioned, externally peer reviewed.

Declaration of interests

The authors declare that they have no competing interests.

Funding

This work was supported by UK Medical Research Council (MRC)/ Newton Fund, grant number MR/R020485/1. The funding body has no role in the study design, collection, analysis, or interpretation of data, in the writing of the manuscript, or the decision to submit the report for publication.

References

1. Daru, J., et al., *Risk of maternal mortality in women with severe anaemia during pregnancy and post partum: a multilevel analysis.* Lancet Glob Health, 2018. **6**(5): p. e548-e554.
2. Haider, B.A., et al., *Anaemia, prenatal iron use, and risk of adverse pregnancy outcomes: systematic review and meta-analysis.* BMJ, 2013. **346**: p. f3443.
3. Black, R.E., *Global Distribution and Disease Burden Related to Micronutrient Deficiencies.* Nestlé Nutrition Institute Workshop Series, 2014. **78**: p. 21-28.
4. Khan, K.S., et al., *WHO analysis of causes of maternal death: a systematic review.* The Lancet, 2006. **367**(9516): p. 1066-1074.
5. Kozuki, N., et al., *Moderate to severe, but not mild, maternal anemia is associated with increased risk of small-for-gestational-age outcomes.* J Nutr, 2012. **142**(2): p. 358-62.
6. Global Nutrition Report, *The state of global nutrition.* 2021, Development Initiatives: Bristol, UK.
7. Ministry of Health Nepal, New ERA, and ICF, *Nepal Demographic and Health Survey 2016. .* 2017, Ministry of Health, Nepal.
8. Ministry of Health and Population (MOHP) [Nepal], N.E., and ICF International Inc. , *Nepal Demographic and Health Survey 2011*. 2012, Ministry of Health and Population, New ERA, and ICF International, Calverton, Maryland.

For peer review only

9. Pasricha, S.-R., et al., *Reducing anaemia in low income countries: control of infection is essential.* BMJ, 2018. **362**: p. k3165.

10. Camaschella, C., *Iron deficiency: new insights into diagnosis and treatment.* Hematology Am Soc Hematol Educ Program, 2015. **2015**: p. 8-13.

11. Petry, N., et al., *The Proportion of Anemia Associated with Iron Deficiency in Low, Medium, and High Human Development Index Countries: A Systematic Analysis of National Surveys.* Nutrients, 2016. **8**(11): p. 693.

12. Pena-Rosas, J.P., et al., *Daily oral iron supplementation during pregnancy.* Cochrane Database Syst Rev, 2015(7): p. CD004736.

13. Salam, R.A., J.K. Das, and Z.A. Bhutta, *Effect of mass deworming with antihelminthics for soil-transmitted helminths during pregnancy.* Cochrane Database Syst Rev, 2021. **5**: p. CD005547.

14. Malhotra, N., et al., *The role of maternal diet and iron-folic acid supplements in influencing birth weight: evidence from India's National Family Health Survey.* J Trop Pediatr, 2014. **60**(6): p. 454-60.

15. Imdad, A. and Z.A. Bhutta, *Routine iron/folate supplementation during pregnancy: effect on maternal anaemia and birth outcomes.* Paediatr Perinat Epidemiol, 2012. **26 Suppl 1**: p. 168-77.

16. Cogswell, M.E., et al., *Iron supplementation during pregnancy, anemia, and birth weight: a randomized controlled trial.* Am J Clin Nutr, 2003. **78**(4): p. 773-81.

17. Christian, P., et al., *Antenatal and postnatal iron supplementation and childhood mortality in rural Nepal: a prospective follow-up in a randomized, controlled community trial.* American journal of epidemiology, 2009. **170**(9): p. 1127-36.

18. Perez, E.M., et al., *Mother-Infant Interactions and Infant Development Are Altered by Maternal Iron Deficiency Anemia.* The Journal of Nutrition, 2005. **135**(4): p. 850-855.

19. Yadav, K.D., et al., *Compliance of iron and folic acid supplementation and status of anaemia during pregnancy in the Eastern Terai of Nepal: findings from hospital based cross sectional study.* BMC Research Notes, 2019. **12**(1): p. 127.

20. Singh, D.R., et al., *The perils of COVID-19 in Nepal: Implications for population health and nutritional status.* J Glob Health, 2020. **10**(1): p. 010378.

21. KC, A., et al., *Effect of the COVID-19 pandemic response on intrapartum care, stillbirth, and neonatal mortality outcomes in Nepal: a prospective observational study.* The Lancet Global Health, 2020.

For peer review only

22. Singh, R. and M. Subedi, *COVID-19 and stigma: Social discrimination towards frontline healthcare providers and COVID-19 recovered patients in Nepal.* Asian J Psychiatr, 2020. **53**: p. 102222.

23. Ministry of Health and Population, *Department of Health Services Annual Report 2076/77 (2019/20)*. 2020.

24. Ministry of Health and Population, *Department of Health Services Annual Survey 2075/6 (2018/19)*. 2019.

25. Ministry of Health and Population, *Nepal HMIS Local Level Raw Data 2076-77 (2019-20)*. 2020, Department of Health Services: Kathmandu, Nepal.

26. Ministry of Health and Population, *Nepal HMIS Database 2075-76 (2018-2019) by Local Government*. 2019, Department of Health Services Kathmandu, Nepal.

27. Girard, A.W. and O. Olude, *Nutrition education and counselling provided during pregnancy: effects on maternal, neonatal and child health outcomes.* Paediatric and Perinatal Epidemiology, 2012. **26 Suppl 1**: p. 191-204.

28. Morrison, J., et al., *Formative qualitative research to develop community-based interventions addressing low birth weight in the plains of Nepal.* Public Health Nutr, 2018. **21**(2): p. 377-384.

29. Morrison, J., et al., *Addressing anaemia in pregnancy in rural plains Nepal: A qualitative, formative study.* Matern Child Nutr, 2021. **17 Suppl 1**: p. e13170.

30. Harris-Fry, H., et al., *Relative power: Explaining the effects of food and cash transfers on allocative behaviour in rural Nepalese households.* Journal of Development Economics, 2022. **154**: p. 102784.

31. Rimal, R.N., et al., *Iron and folic acid consumption and changing social norms: cluster randomized field trial, Odisha, India.* Bull World Health Organ, 2021. **99**(11): p. 773-782.

32. Fiedler, J., et al., *Key facets to build up eHealth and mHealth interventions to enhance physical activity, sedentary behavior and nutrition in healthy subjects–an umbrella review.* BMC Public Health, 2020. **20**(1): p. 1-21.

33. Galle, A., et al., *A double-edged sword-telemedicine for maternal care during COVID-19: findings from a global mixed-methods study of healthcare providers.* BMJ Glob Health, 2021. **6**(2).

34. Bhatta, R., K. Aryal, and G. Ellingsen, *Opportunities and Challenges of a Rural-telemedicine Program in Nepal.* J Nepal Health Res Counc, 2015. **13**(30): p. 149-53.

For peer review only - http://bmjopen.bmj.com/site/about/guidelines.xhtml

35. Chan, A.-W., et al., *SPIRIT 2013 Statement: Defining Standard Protocol Items for Clinical Trials.* Annals of Internal Medicine, 2013. **158**(3): p. 200-207.

36. Central Bureau of Statistics, N., *National Population and Housing Census 2011 (National Report)*. 2012, Government of Nepal: Kathmandu, Nepal.

37. Wallerstein, N. and E. Bernstein, *Empowerment education: Freire's ideas adapted to health education.* Health education quarterly, 1988. **15**(4): p. 379-394.

38. Moore, G.F., et al., *Process evaluation of complex interventions: Medical Research Council guidance.* BMJ, 2015. **350**: p. h1258.

39. Dimagi, *CommCare*. 2020.

40. Saville, N.M., et al., *Comprehensive Anaemia Programme and Personalized Therapies (CAPPT): protocol for a cluster-randomised controlled trial testing the effect women's groups, home counselling and iron supplementation on haemoglobin in pregnancy in southern Nepal.* Trials, 2022. **23**(1): p. 183.

41. Gautam, S., et al., *Determining factors for the prevalence of anemia in women of reproductive age in Nepal: Evidence from recent national survey data.* PLoS One, 2019. **14**(6): p. e0218288.

42. Department of Health Services, *Standard Treatment Protocols for Health Posts and Sub-Health posts.* 2012, Ministry of Health and Population: Kathmandu, Nepal.

For peer review only

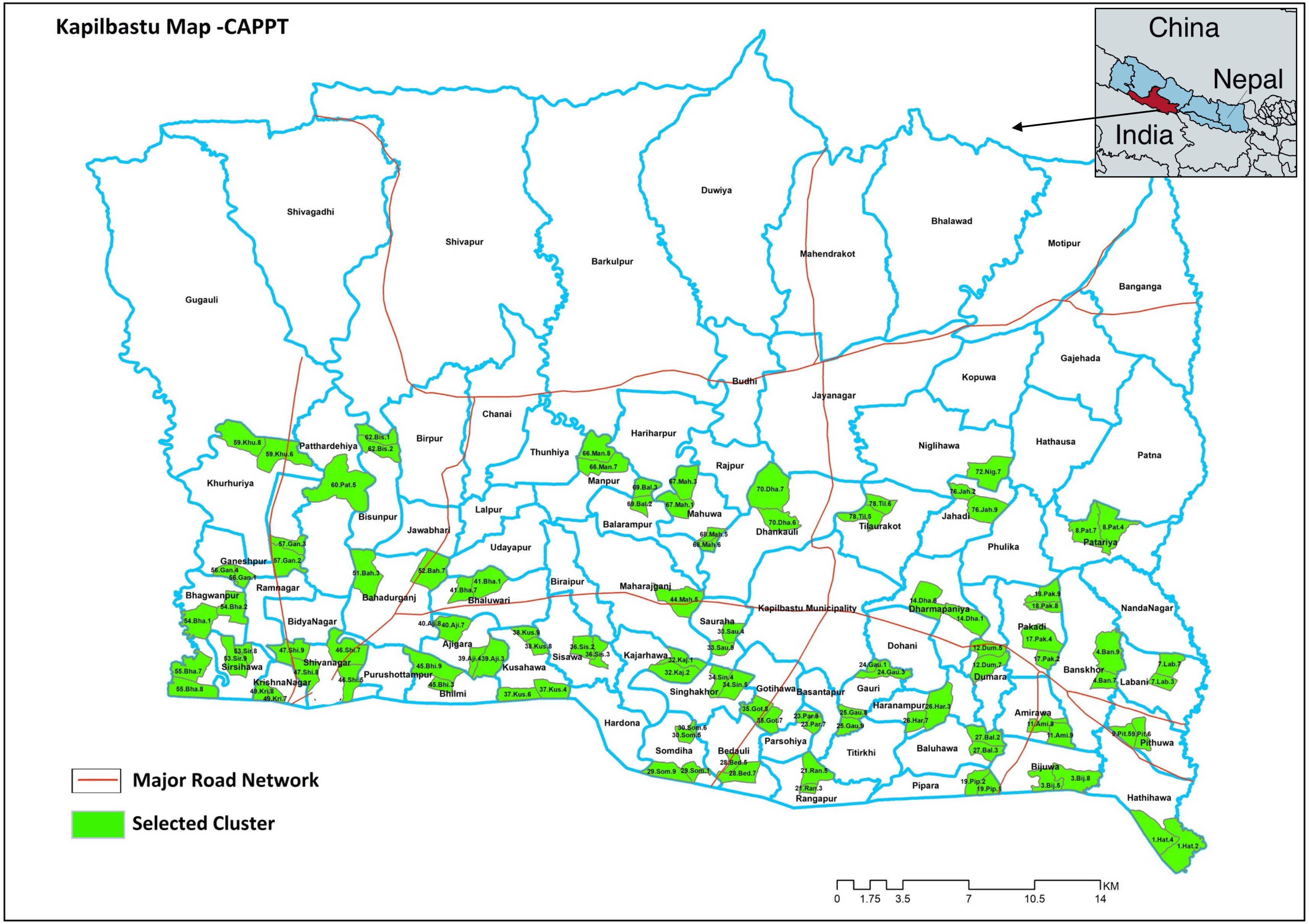
Kapilbastu Map -CAPPT
China
Nepal
India
Shivagadhi
Shivapur
Barkulpur
Duwiya
Mahendrakot
Bhalawad
Motipur
Banganga
Gugauli
Budhi
Jayanagar
Kopuwa
Gajehada
Chanai
Birpur
Patthardehiya
Hariharpur
Thunhiya
Rajpur
Niglihawa
Hathausa
Patna
Khurhuriya
Manpur
Mahuwa
Dhankauli
Tilaurakot
Jahadi
Phulika
Patariya
Bisunpur
Lalpur
Jawabhari
Balarampur
Udayapur
Ganeshpur
Ramnagar
Bahadurganj
Bhaluwari
Biraipur
Maharajganj
Kapilbastu Municipality
Dharmapaniya
NandaNagar
Bhagwanpur
BidyaNagar
Sauraha
Pakadi
Ajigara
Dohani
Sirsihawa
Shivanagar
Purushottampur
Kusahawa
Sisawa
Kajarhawa
Dumara
Banskhor
Labani
KrishnaNagar
Bhilmi
Singhakhor
Gotihawa
Basantapur
Gauri
Haranampur
Amirawa
Hardona
Parsohiya
Titirkhi
Baluhawa
Pithuwa
Somdiha
Bedauli
Bijuwa
Rangapur
Pipara
Hathihawa
Major Road Network
Selected Cluster
0
1.75
3.5
7
10.5
14
KM

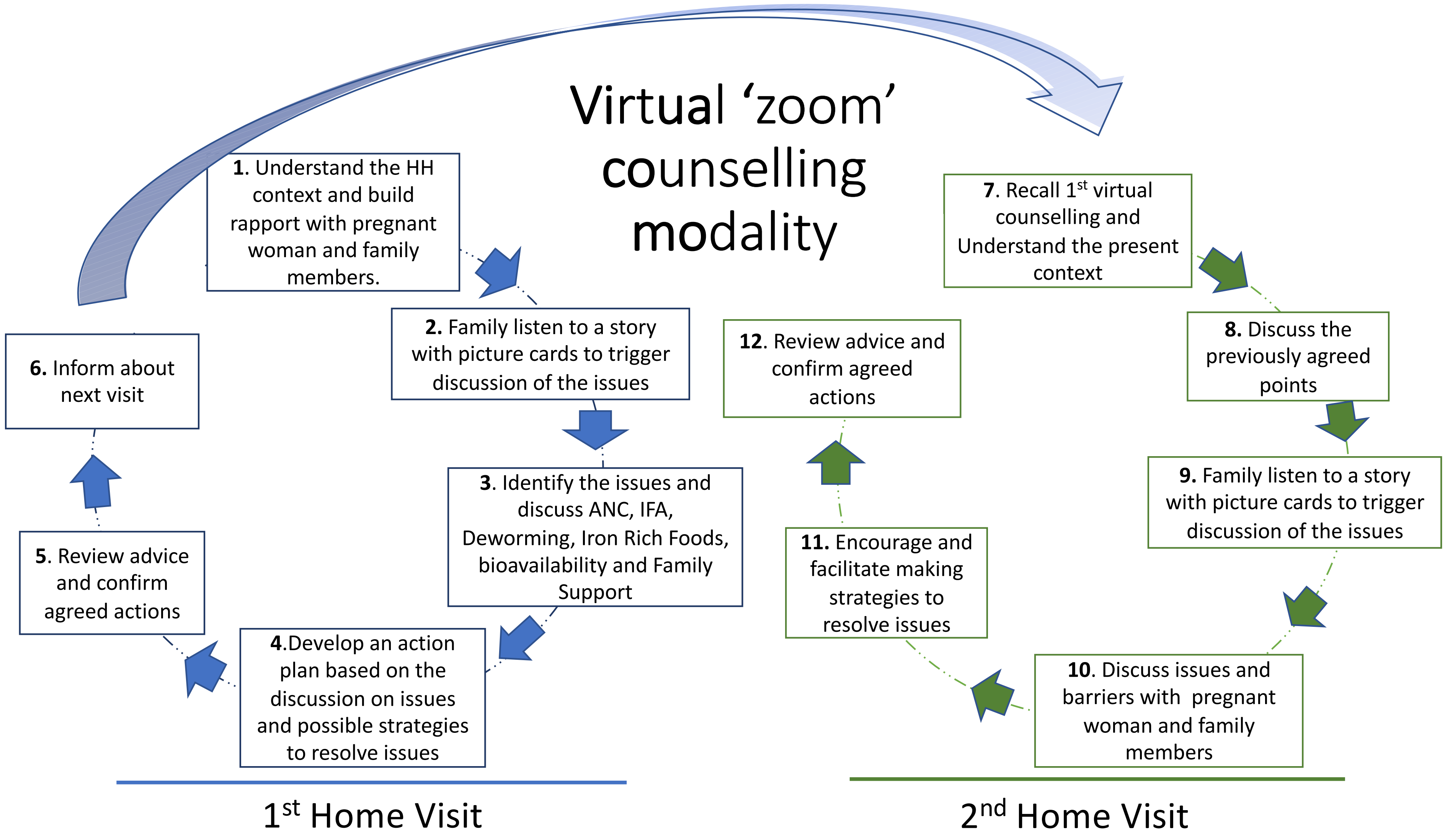
Virtual 'zoom' counselling modality
1. Understand the HH context and build rapport with pregnant woman and family members.
2. Family listen to a story with picture cards to trigger discussion of the issues
3. Identify the issues and discuss ANC, IFA, Deworming, Iron Rich Foods, bioavailability and Family Support
4.Develop an action plan based on the discussion on issues and possible strategies to resolve issues
5. Review advice and confirm agreed actions
6. Inform about next visit
7. Recall 1st virtual counselling and Understand the present context
8. Discuss the previously agreed points
9. Family listen to a story with picture cards to trigger discussion of the issues
10. Discuss issues and barriers with pregnant woman and family members
11. Encourage and facilitate making strategies to resolve issues
12. Review advice and confirm agreed actions
1st Home Visit
2nd Home Visit

**Intervention**	**Pregnant woman and her household members**<br>Dialogue and problem solving in two virtual 'zoom' counselling session on a tablet					
Awareness and health literacy (household members and pregnant woman)	Awareness of iron-rich food sources	Awareness of ways to improve bioavailability	Awareness of the need for iron-rich diet & iron and folic acid tablets (IFA)	Awareness of ways to reduce side-effects of iron and folic acid (IFA)	Awareness of role of HH in improving diets, increasing access to antenatal care and supporting IFA consumption	
Household actions to support Pregnant Woman	Encourage healthy snacking	Cooking and serving practices to increase Pregnant Women's access to iron-rich diet	Encourage / enable dietary practices to increase bioavailability of iron	Encourage / enable practices to minimise side-effects of IFA	Encourage / enable consumption of IFA	Accompany to ANC & retrieve re-supply of IFA
Enabling environment	Increased household support to Pregnant Woman		Men take a more active role in supporting Pregnant Woman		IFA and 'eating well' not linked with having a large baby	
Intermediate outcomes	Improved intake of iron-rich foods	Improved dietary diversity	Improved actions to increase iron absorption	Improved uptake of Antenatal Care	Improved access and uptake of IFA	Improved access to and uptake of de-worming
**Primary / secondary outcomes**	**Improved compliance in taking Iron and Folic Acid tablets**<br>**Dietary modifications to increase iron consumption / absorption in Pregnant Woman**					
'Higher level' outcomes not measured	Improved dietary iron adequacy	Improved absorption of iron from diet	Improved overall dietary adequacy	Improved micronutrient status	Higher haemoglobin / Less anaemia	Improved physical health of mother and baby

Legend: IFA Iron Folic Acid

	STUDY PERIOD					
	**Enrol-ment**	**Alloc-ation**	**Post-allocation**			**Close-out**
**TIMEPOINT****	t_0 Baseline interview		t_1 (t_0 + 1 to 7 days or more#)	t_2 (t_1 + 1 to 7 days or more#)	t_3 (t_2 + 14 days or more#)	t_x *Endline interview* (t_0 + 49 to 70 days ideally but permitted up to delivery#)
**ENROLMENT at baseline interview:**						
Eligibility screen for baseline	X					
Informed consent for baseline	X					
Eligibility screen for trial	X					
Informed consent for trial	X					
**Allocation**		X				
**INTERVENTIONS:**						
Tablet handover and orientation			X			
Virtual counselling session 1				X		
Virtual counselling session 2					X	
**ASSESSMENTS:**						
Baseline survey variables:<br>Socioeconomic indicators / food security<br>Marriage / Pregnancy history<br>Uptake / quality of antenatal care (ANC)<br>Iron and Folic Acid (IFA) access/consumption<br>Deworming access/consumption<br>Dietary Diversity Score<br>Knowledge and intake of key iron-rich foods<br>Practice of iron-absorption enhancement	X					
Process variables<br>**Post-counselling form**<br>Ease of conducting counselling<br>Who attended counselling?<br>Pregnant woman's level of comfort<br>Pregnant woman's attentiveness<br>Engagement of family members<br>Actions agreed upon with family				X	X	
**plus in Post-2nd counselling session only**<br>Extent actions were implemented<br>Factors preventing / facilitating taking actions					X	
Endline interview variables:<br>Uptake / quality of antenatal care (ANC)<br>Iron and Folic Acid (IFA) access/consumption<br>Deworming access/consumption<br>Dietary Diversity Score<br>Knowledge and intake of key iron-rich foods<br>Practice of iron-absorption enhancement<br>Confidence in health knowledge<br>Exposure and response to intervention<br>Awareness of COVID-19 prevention, vulnerability and symptoms						X

Note that although target follow-up periods were defined at outset, lack availability of respondents to attended counselling and/or interviews means that gaps between time points are varied.

For peer review only

Information sheet for VALID Baseline survey with Pregnant Women

Introduction

Namaste! My name is ____________________. I have come from HERD International located in Thapathali, Kathmandu. HERD International is a national level research organization. This organization has been conducting various programmes and research upon health, environment and social development. Currently, HERD International, in partnership with University College London, is conducting a study that aims to reduce anaemia in pregnant women in Kapilbastu. I would like to invite you to be a part of this study.

Before you decide whether to participate, it is important for you to understand why this research is being done and what participation will involve. I will read what is written in this information sheet aloud to you. You can ask me if there is anything that you do not understand or if you want more information. You will be given a copy of this information sheet. Take your time to decide whether or not you want to take part in the study or not. Thank you for reading this/listening to me.

Details of the Study

Anaemia is a condition when there is decreased haemoglobin in blood, it is associated with illness and complications during pregnancy and childbirth. HERD International, in partnership with University College London, is conducting a study with an aim to reduce anaemia in pregnant women. This study is aiming to improve anemia status among pregnant women in the community through virtual counselling session on nutrition, diet and antenatal care. Therefore, we would like to invite you to participate in this baseline survey we are conducting to understand your eligibility for the follow up study, socio demographics, dietary habits and antenatal care seeking behavior among pregnant women in the study area.

Why this survey?

We are conducting this study in 54 clusters or study areas in Kapilvastu district. Iron deficiency anemia is the main reason for anemia among pregnant women. Pregnant women who are anaemic are much more likely to die during childbirth than those women who are not and their infants are more likely to be born small for gestational age. During the baseline survey we will visit homes of all pregnant women in the study area to collect data, we expect to interview at least 1000 pregnant women, out of these women we hope to enroll at least 300 who are eligible (≤28 weeks' gestation) into the follow up study.

Why am I being invited to participate in this survey?

You can take part in this study if you are a pregnant woman or girl aged 13 to 49 years, live in the study area and are able to respond to the survey questions. If you are interested to take part in the study, we will give you this information sheet to read or have others read to you. Once you have listened-to (or read) and understood the information sheet we will ask you to give us sign the consent form agreeing to participate in the survey. If you

For peer review only

decide to participate, we will ask personal questions about you and your family (for eg: household head name, family size, ethnicity, religion, income and education) as well as your dietary habits, health problems and utilization of health care.

Will my taking part in this project be kept confidential?

All the information that we collect about you during the course of the research will be kept strictly confidential. Your information will be recorded using tablets by the researchers are involved in finding you to interview you. Only they will have access to your name and address, all other researchers who look at the information you share with us will not be able to identify you as your name and address will be removed.

Ethical approval

This study has been approved by the Nepal Health Research Council Approval ID number 570/2021.

Agreeing to take part

Your participation is voluntary. If you don't want to take part, you can refuse without giving a reason. If you decide to take part in the study, you will be given this information sheet to keep and be asked to sign or thumb print the consent. If you agree to participate and then change your mind at any time, please tell us and we will stop the interview at any point.

Are there any risks if you participate?

We do not think that any harm will come to you, but it is possible that you might find sharing information about your pregnancy uncomfortable or upsetting. You don't have to continue to take part if you don't feel like it.

Are there any benefits if you participate?

There is no direct benefit of participating in this study. However, the findings from this study will generate evidence to help develop health and nutrition programs and policies for pregnant women in future.

Contact for further information

You are encouraged to ask any questions you wish, before, during or after getting involved. If you require any further information or have any concerns while taking part in the study you can contact:

HERD International, Prasuti Griha Marg, Thapathali, Kathmandu. Tel 01-4238045

HERD International District Office, Taulihawa, Kapilbastu. Tel: number: 076-590090

Thank you for listening to / reading this information sheet and for considering whether to take part in this research study.

For peer review only

Virtual Antenatal Intervention for improved Diet and Iron intake (VALID) Trial

Participant Information Sheet for Pregnant Women

Introduction

Namaste! My name is ____________________. I have come from HERD International located in Thapathali, Kathmandu. HERD International is a national level research organization. This organization has been conducting various programmes and research upon health, environment and social development. Currently, HERD International, in partnership with University College London, is conducting a study that aims to reduce anaemia in pregnant women in Kapilbastu. I would like to invite you to be a part of this study.

Before you decide whether to participate, it is important for you to understand why this research is being done and what participation will involve. I will read what is written in this information sheet aloud to you. You can ask me if there is anything that you do not understand or if you want more information. You will be given a copy of this information sheet. Take your time to decide whether or not you want to take part in the study or not. Thank you for reading this/listening to me.

Details of the study

HERD International, in partnership with University College London, is conducting a study with an aim to reduce anaemia in pregnant women. The Medical Research Council (UK) is funding this research.

Anaemia is a condition when there is decreased haemoglobin in blood, and this is caused by various factors. In Nepal, lack of iron is the most common cause of anaemia in pregnancy. It is important to reduce anaemia in pregnancy because low iron levels are associated with illness and complications during pregnancy and childbirth. Pregnant women who are anaemic are much more likely to die during childbirth than those women who are not and their infants are more likely to be born small for gestational age.

In Nepal, Kapilbastu is one of the districts where anaemia is highly prevalent. Hence, we have chosen 54 clusters (103 old-wards) within 9 pallikas of Kapilbastu for this study. The government of Nepal recommends minimum 4 ANC visits during pregnancy and consumption of 180 tablets of iron and folic acid starting from 20 week's gestation. However, compliance to these recommendations is low, increasing the risk of anemia during pregnancy. The situation has become worse since March 2020 with ongoing COVID-19 imposed travel restrictions and lockdowns, which have made health care less accessible. The health and nutrition status of pregnant women has deteriorated as women are afraid to visit health facilities to seek care.

Objective:

The VALID trial is designed to assess if providing antenatal virtual counselling on a tablet increases compliance to intake of the required dosage of IFA tablets, and improves dietary diversity and dietary practices, compared with women who have access to routine antenatal care (ANC) only (control).

For peer review only

This trial is being implemented in 9 pallikas of Kapilvastu district among 13 to 49 years old married pregnant women. Around 300 pregnant women will be enrolled and allocated to either of the 2 trial arms.

In the intervention arm, in addition to routine pregnancy care, 150 pregnant women will be provided with electronic tablets (like large Smart Phones) which are fitted with sim cards to receive virtual counselling sessions. A female member of HERD staff who is qualified as an ANM (a “nutrition assistant”) will visit you in your home to lend you a tablet to provide virtual counselling session. After this she will provide you with two virtual counselling sessions: one between 12 to 28 week’s gestation and another two weeks after the first session. At each session she will discuss with you and your family members about your diet and health in pregnancy and give you advice based on what you are already eating / doing. She will encourage you to discuss and develop an action plan to improve your diet, consume iron tablets and antenatal care to minimize the risk of anaemia during pregnancy.

In the control arm, 150 pregnant women will be encouraged to go for routine pregnancy related services provided by the government of Nepal that includes antenatal care and iron and folic acid tablets.

Who are we inviting to participate?

You can take part in this research if you are a pregnant woman or girl aged 13 to 49 years, less than 28 weeks of gestation, planning to live in ,and/or seek services from the health facilities in, the study area and are able to respond to the survey questions. Pregnant women will be enrolled with the help of the health workers or female community health volunteers in these communities. If you are interested to take part in the trial, we will give you this trial information sheet to read or have others read to you. Once you have listened-to (or read) and understood the information sheet we will ask you to give us sign the consent form agreeing to participating in the trial. The risk or danger of participating in the trial is minimal. Participation in this trial is voluntary. If for any reason you want to withdraw your consent you can do so at any time.

What will happen if you agree to take part in this study?

If you decide to take part and give your consent (by signing or thumb-printing a consent form), first I will give you a unique identification number. On a tablet, I will record some personal details about you such as your age, education, household details, obstetric history, eating habits, iron supplements, physical activity and antenatal care. After responding to these questions, you will be assigned randomly to one of the two arms of the trial (intervention or control). It will take around 90 minutes to answer the questions and to randomly assign you to the trial arm.

Regardless of which arm you are assigned to, I will visit you again in about one month to enquire about your eating habits, iron and folic acid consumptions, antenatal care during pregnancy. This will take additional 90 minutes or so.

For peer review only - http://bmjopen.bmj.com/site/about/guidelines.xhtml

But if you are assigned to the intervention arm (virtual counselling) we will provide you a tablet with sim card to use during the intervention. You will receive two virtual counselling sessions 2 to 3 weeks apart and each session will last around 90 to 120 minutes. During these sessions we will discuss with you and your family members the importance of eating iron rich food during pregnancy, identify problems or barriers to eating iron rich food, identify solutions and develop action plans to implement them.

Therefore, if you are assigned to the intervention group, overall, you will need to provide around 5 hours' time, whereas if you fall in the control group (routine care) group you will need to provide 3 hours' minutes to the trial.

At the end of data collection, we will transfer 200 rupees' worth of mobile top up to your cell phone number or to a number of a person of your choice if you do not have your own cell phone.

Are there any risks if you participate?

We do not think that any harm will come to you, but it is possible that you might find sharing information about your pregnancy uncomfortable or upsetting. You don't have to continue to take part if you don't feel like it. If you would like to talk to someone about the feelings generated by the questions, please contact a member of HERD staff.

Are there any benefits if you participate?

There is no direct benefit of participating in this study, however you might enjoy interacting with the counsellor on the tablet. If found effective and feasible, the results of this trial will provide evidence that counselling can be provided virtually to improve health and nutrition of pregnant women from excluded communities in Kapilbastu and rest of Nepal.

Will my taking part in this project be kept confidential?

All the information that we collect about you during the course of the research will be kept strictly confidential. Only researchers directly associated with this project, who are involved in finding you to interview you, will have access to your name and address. All other researchers who look at the information you share with us will not be able to identify you as your name and address will be removed. You will be allocated a unique number, which will be used as a code to identify you instead of your name. You will not be able to be identified in any ensuing reports or publications.

If you consent to take part in this study, the records obtained while you are in this study (age, ethnicity, religion, education and so on) will remain strictly confidential at all times. The information will be held securely on either paper or electronically at HERD International and in University College London in the UK under the provisions the local Data Protection laws. Your name will not be passed to anyone else outside the research team who is not involved in the trial. Your records will be available to people authorized to work on the trial and those responsible for ensuring that the study is carried out correctly. By signing the consent form you agree to this access for the

For peer review only

current study. Further research might involve other researchers using the information you give us, but without your name attached to it. Alternatively, we or other researchers might seek to find you in the future to undertake further research with you.

If you withdraw consent from further study, unless you object, your data and samples will remain on file and will be included in the final study analysis.

Ethical approval

This study has been approved by the Nepal Health Research Council Approval ID number 570/2021.

Agreeing to take part

Your participation is voluntary. If you don't want to take part, you can refuse without giving a reason. If you decide to take part in the study, you will be given this information sheet to keep and be asked to sign or thumb print the consent. If you agree to participate and then change your mind at any time, please tell us and we will stop visiting you or telephoning you. We will take a photo of the consent form with your signature which will be filed in your records. You can have more time to think this over if you are at all unsure.

Data safety procedure

All participants will need to provide information two times during the trial, once at enrolment ≤ 28 weeks' gestation and another at 16 to 33 weeks' gestation. Depending on the COVID-19 situation, the information will be collected either over the phone or in person. Data assistants will assess eligibility to confirm pregnancy and gestational age, and take written consent before collecting data. The collected data will be kept confidential, stored in protected servers that can be assessed by authorised personnel only.

Expected outcome of the Trial"

In this study, the virtual nutritional counselling given to pregnant women through a tablet will help us to understand what kinds of improvements can be made in the regular intake of iron and folic acid, the consumption of iron-rich foods, and antenatal check-ups during pregnancy. As nutritional assistants will encourage antenatal check-ups and institutional delivery during the virtual counselling sessions, we hope that antenatal check-ups and institutional delivery will increase.

Use of the trial results:

The evidence generated by the trial can guide government and other stakeholders in development of plans and programs for maternal nutrition and to reduce the burden of anaemia during pregnancy.

Contact for further information

For peer review only

You are encouraged to ask any questions you wish, before, during or after getting involved. If you have any questions about the study, please speak to the HERD International researchers who visit you, who will be able to provide you with up to date information about the trial. If you require any further information or have any concerns while taking part in the study you can contact:

Trial Manager, HERD International, Prasuti Griha Marg, Thapathali, Kathmandu. Tel 01-4238045

Or

Dr Naomi Saville, Senior Research Associate, University College London Institute for Global Health and Technical advisor to HERD, Kathmandu Nepal. Tel: 01-4238045

HERD International District Office, Taulihawa, Kapilbastu. Tel: number: 076-590090

Thank you for listening to / reading this information sheet and for considering whether to take part in this research study.

For peer review only

Patient Identification Number for this trial:

CONSENT FORM

Title of Study: Virtual Antenatal Intervention for improved Diet and Iron intake (VALID) Trial

Please complete this form after you have read the Information Sheet and/or listened to an explanation about the research.

Name and Contact Details of the Researcher(s):

DR SUSHIL BARAL 9851068940 and DR NAOMI SAVILLE 9851017232

This study has been approved by the Nepal Health Research Council Approval ID number 570/2021.

Thank you for considering taking part in this research. The researcher who has come to your home or who you meet with over the telephone must explain the project to you before you agree to take part. If you have any questions arising from the Information Sheet or explanation already given to you, please ask the researcher before you decide whether to join the study. You will be given a copy of this Consent Form to keep and refer to at any time.

I confirm that I understand that by ticking/initialling each box below I am consenting to this element of the study. I understand that it will be assumed that unticked boxes means that I DO NOT consent to that part of the study. I understand that by not giving consent for any one element that I may be deemed ineligible for the study.

In case of the married girl of age 14 to 19 running years (13 to 18 completed years), take the consent from at least one of her guardians as well. This means filling the tick box columns with the participant **and** ***her guardian and getting the signature or thumb print of both of them.***

	Pregnant woman's Tick Box	Guardian's Tick box
I confirm that I have read the Information Sheet for the above study (or it has been read to me in an appropriate language) and I understood its content.		

For peer review only

I have had an opportunity to consider the information and what will be expected of me and to ask questions which have been answered to my satisfaction.		
I understand that my participation in this research is completely voluntary. I understand that I am free to withdraw from the research at any time without any repercussions. I understand that I can halt an interview at any point if I feel uncomfortable.		
I understand the potential risks of participating and the support that will be available to me should I become distressed during the course of the research.		
I understand that I will not benefit financially from this study or from any possible outcome it may result in in the future.		
I understand that my personal information *(including ethnicity, age, socio-economic and educational status, past medical and obstetric history)* will be used when analysing the data, but not my name.		
I understand that all personal information will remain confidential and that all efforts will be made to ensure I cannot be identified by anyone, except those who are involved in meeting me to ask questions.		
I understand that my data gathered in this study will be securely stored anonymously (without my name and address) and that it will not be possible to identify me in any publications.		
I understand that the data will not be made available to any commercial organisations but are solely the responsibility of the researcher(s) undertaking this study.		
I agree that my anonymised research data (that has my name and address removed) may be used by others for future research. I understand that no one will be able to identify you me if/when this these data are shared.		
I would be happy for the data I provide, including my name and address, to be archived in the UCL Data Safe Haven to be kept there, and other secure locations in Nepal and UK, in case researchers need to find me again in the future. I understand that these personally identifiable data will not be available to anyone except authorised researchers who need my name and address to find me again in the future.		

For peer review only

I understand that when I consent to have my photos taken while engaging in the research activities during the research period, that these could be published on HERD international's or UCL's website or other publications.		
I would be happy to be contacted in future by HERD International, UCL and/or other researchers who would like to invite me to participate in follow-up studies to this project (for example if someone came to measure me or my child after this study ended) .		
I have informed the researcher of any other research in which I am currently involved or have been involved in during the past 12 months.		
**I consent to participate in this study**		

Phone no for future contact:

Signing this document means that you voluntarily agree to participate in this research after understanding the information provided to you.

(In case the married participant's age is between 14 and 19 running years (13 -18 completed years)

______________________ ______________ ________________

Name of guardian Date Signature

OR Guardian's Finger Prints

Left	Right

For peer review only

Name of participant	Date	Signature

OR Participant's Finger Prints

Left	Right
	Left

Researcher (Data Assistant)	Date	Signature

For peer review only

Nepali version of the Participnt information sheet

परियोजना परिचय: गर्भावस्थामा आईरन-फोलिक एसिड को सेवनमा सुधारका लागि भर्चुअल परामर्श कार्यको अध्ययन (VALID)

काठमाण्डौ स्थित हर्ड ईन्टरनेशनल एक राष्ट्रिय स्तरको अनुसन्धान गर्ने संस्था हो। यस संस्थाले स्वास्थ्य, वातावरण, र सामाजिक क्षेत्रमा उल्लेख्य मात्रामा कार्यक्रम तथा अनुसन्धानहरु गर्दै आईरहेको छ। हर्ड ईन्टरनेशनलद्धारा गर्भवती महिलाहरुको आईरन- फोलिक एसिड सेवनको अवस्थामा सुधार गर्नका लागि लुम्बिनी प्रदेशको कपिलवस्तु जिल्लामा "गर्भावस्थामा भर्चुअल परामर्श कार्यको अध्ययन" नामक अनुसन्धान गर्न लागेको व्यहोरा अवगत गराउन चाहन्छौ। यस अनुसन्धान सञ्चालनका लागि यस संस्थाले नेपाल स्वास्थ्य अनुसन्धान परिषद् बाट स्विकृति प्राप्त गरिसकेको छ र स्वास्थ्य सेवा विभाग अन्तर्गत परिवार कल्याण महाशाखालाई अवगत गराएको छ। यस अध्ययन शुरू गर्नु अघि हामी प्रदेश तथा स्थानिय सरकारसंग समन्वय गरी आवश्यक सहयोग पनि लिनेछौं ।

पृष्ठभूमि

रक्तअल्पता भनेको रगतमा रातो कोशिकाको मात्रामा सामान्यतयाभन्दा कम हुनु वा प्रत्येक रातो कोशिकामा सामान्यतयाभन्दा कम हेमोग्लोबिन हुनु हो। आईरनको कमी नै रक्तअल्पता हुनुको सामान्य कारण हो। गर्भावस्थामा २० प्रतिशत मातृ मृत्यु सिधै रक्तअल्पतासँग सम्बन्धित छ । गम्भिर रक्तअल्पताका कारण विश्वमा हुने आधाभन्दा बढी मातृ मृत्यु दक्षिण एसियामा हुने गर्दछ। आईरनको कमीले गर्भावस्थामा जटिलता र अस्वस्थता हुने भएकाले गर्भवती अवस्थामा हुने रक्तअल्पतालाई घटाउन निकै जरुरी छ। बच्चा जन्माउने क्रममा रक्तअल्पता नभएका गर्भवती महिला भन्दा रक्तअल्पता भएका गर्भवती महिलाको सम्भवत मृत्यु दर बढी देखिन्छ र उनीहरुबाट कम तौल भएका शिशु जन्मने गर्दछन्।

नेपालको सन्दर्भमा अन्य क्षेत्रमा भन्दा गर्भवती महिलामा हुने रक्तअल्पता तराईमा (३६.४%) बढी देखिन्छ। सबै प्रदेशहरु मध्ये, उच्च रक्तअल्पता हुनेमा लुम्बिनी प्रदेश (४३.५%) दोश्रो स्थानमा पर्दछ। नेपालमा नियमित गर्भ जाँच गरिने (न्युनतम ४ पटक) र गर्भ रहेको २० हप्ता देखि नियमित आइरन तथा फोलिक एसिड (१८० चक्की) वितरण गरिने सरकारी प्रोटोकोल भएता पनि सरकारी प्रोटोकोल अनुसार नियमित गर्भ जाँच गर्ने महिलाहरुको संख्या न्युन रहेको र वितरण हुने आइरन फोलिक एसिड मा पहुच कम रहेको छ। जसले गर्दा गर्भावस्थामा हुने रक्तअल्पता अझै उच्च देखिन्छ। वर्तमान कोभिड-१९ को महामारी, यस समयमा स्वास्थ्य संस्था जानका लागि मानिसमा रहेको त्रास, महामारी नियत्रणका लागि पटक-पटक गरिएका विभिन्न स्वरुपका निषेधाज्ञाले गर्दा स्वास्थ्य सेवामा मानिसहरुको पहुच सिमित भएको छ र मातृ तथा शिशु पोषण स्थितीमा ह्रास आएको छ।

उद्देश्य

यस अध्ययनको उद्देश्य यस गर्भावस्थामा आइरन-फोलिक एसिडको सेवन नियमितता गराई आइरनको कमीले हुने रक्तअल्पता घटाई मातृ पोषणमा सुधार ल्याउनुको साथै उपलब्ध स्वास्थ्य सेवाको उपयोग र पहूँच बढाउनु हो।

विधिहरु

यो अध्ययन कपिलबस्तु जिल्लाको ९ वटा पालिकाहरुमा बस्ने १३ देखी ४९ वर्षका विवाहित गर्भवती महिलाहरुमा सञ्चालन गरिनेछ। हामी यस अध्ययनमा ३०० जना गर्भवती महिलालाई समावेश गराउने योजनामा छौँ, जसलाई दुईवटा समुहमा बाडिनेछ:

१. पहिलो १५० जना महिलाले यस अध्ययनको अवधिका लागी सिमकार्ड सहितको ट्याबलेट प्राप्त गर्नुहुनेछ र हाम्रो पोषण सहायकले ट्याबलेटको माध्यमबाट उँहाहरुलाई रक्तअल्पता, राम्रो खानपानको व्यवहार र गर्भ जाँच सम्बन्धि शिक्षा,परामर्श र सल्लाह प्रदान गर्नुका साथै उँहालाई खानपान सम्बन्धि सल्लाह दिनुहुनेछ। साथै गर्भवती महिलालाई नजिकको स्वास्थ्य संस्थामा गई गर्भवती जाँच गराउन प्रोत्साहन गर्नुहुनेछ। उँहाहरुले गर्भावस्थामा हुने रक्तअल्पताको समस्याका साथै खानपानमा सुधार ल्याएर, आइरन चक्कि सेवन गरेर र गर्भ जाँचका लागि गएर कसरी रक्तअल्पता घटाउन सकिन्छ भन्ने बारे छलफल गर्नुहुनेछ।

२. दोश्रो समूहको १५० जना महिलाले नेपाल सरकारको स्वास्थ्य कार्यक्रमद्वारा प्रदान भईरहेको स्वास्थ्य सेवा सहित नियमित गर्भ जाँच सेवा र आइरन चक्की प्राप्त गर्नेछन्।

हामी कसलाई सहभागी हुनका लागि आमन्त्रण गर्दैछौ?

हामी 12 देखि 24 हप्ता बीचको गर्भवतीहरु लाई मात्र यस अध्ययनमा सहभागी गराउने छौ । यस अध्ययनको लागि आवश्यक पर्ने गर्भवती महिलाहरु हामी स्वास्थ्य संस्था तथा स्वयमसेबीकाको सहायता मार्फत समाबेस गराउने छौ । तपाई यस अध्ययनमा सहभागी हुन योग्य भए नभएको एकीन तपाईको स्थानिय महिला स्वयं सेविका स्वास्थ्यकर्मीहरुले गर्नु हुनेछ । योग्य भएमा, उहाहरुले योग्य गर्भवती महिलाको सहमतिमा सम्पर्क फोन नम्बर

For peer review only

हर्डको तथ्याङ्क सहायकलाई दिनु हुनेछ। यदि तपाई यस ट्रायलमा सहभागी हुन इच्छुक हुनु हुन्छ भने, हामी तपाईलाई अध्ययन बारे जानकारी पत्र पढ्न दिनेछौ साथै यसमा दिएको जानकारी सबै बुझे पछि तपाईले मंजूरीनामा मा हस्ताक्षर गर्नु पर्ने हुन्छ। यस अध्ययनमा सहभागी हुदा हुन सक्ने जोखिम वा खतरा एकदम कम छ। यो अध्ययनमा तपाईको सहभागीता पूर्ण रुपमा स्वैच्छिक हो र यदि केही कारणबस बीचमा छोड्न चाहानुहुन्छ भने पनि तपाईले छोड्न पाउनु हुनेछ ।

यदि तपाई यस अध्ययनमा सहभागी हुनका लागि सहमत हुनुभएमा के हुन्छ ?

यदि तपाई यस ट्रायलमा सहभागी हुन मञ्जुरीनामा दिनुभएमा, हामी तपाईलाई केही ब्यक्तिगत जस्तै परिवार, आम्दानी, प्रजनन स्वास्थ्य आदि र आइरन र् फोलिक ऐसिड सेवन बारे प्रश्नहरु सोध्ने छौ । यो प्रश्नहरु सोधीसके पछि हरेक सहभागी महिलाहरुलाई गोला प्रथा द्वारा (Random) अध्ययनको दुई समूह मध्य कुनै एक समूहमा समाबेश गर्नेछौ। यो सबै प्रक्रिया, प्रश्नहरु सोध्ने र समूहहरु बिभाजन गर्नको लागि लगभग ९० मिनेट समय लाग्ने छ ।

तपाई जुन समुहमा परेपनी, तपाई गर्भवती भएको ३२ देखि ३६ हप्ताको बीचमा हामी पुन: एकपटक प्रश्नहरु सोध्नको लागि भेट्नेछौ (फोन वा प्रत्यक्ष)। त्यो बेलामा हामी तपाईलाई गर्भवती जाँच, खानपान सम्बन्धी, आइरन र फोलिक ऐसिड सेवन, परिवारको सहयोग र अध्ययनमा सहभागी हुँदाको अनुभब बारे प्रश्नहरु सोध्ने छौ । यसको लागि तपाइले लगभग ९० मिनेट समयदिनु पर्ने हुन्छ ।

तर यदि तपाई भर्चुअल परामर्श (virtual counselling) समूहमा पर्नु भयो भने हामी तपाईलाई यस अध्ययन अवधिभरको लागि सिमकार्ड सहितको ट्याबलेट दिनेछौ। त्यसपछि हामी तपाईलाई २ पटक भर्चुअल परामर्शको लागि त्यस ट्याबलेटमा फोन गर्नेछौ र हरेक पटक लगभक ९० देखि १२० मिनेट समय दिनु पर्ने हुन्छ। भर्चुअल परामर्श सत्रमा तपाई र तपाईको परिवारको अन्य सदस्य संग गर्भावास्थामा आइरन युक्त खाने कुराको महत्व र आइरन युक्त खाना खानमा भएका समस्या सम्बन्धि छलफल गर्नेका साथै आवश्यकता अनुसारको परामर्श दिनेछौँ र छलफलको आधारमा समस्या समाधान गर्नको लागि कार्ययोजना बनाउने छौ ।

यो अध्ययनमा सहभागी हुदा यदि भर्चुअल परामर्श समूहमा पर्नु भयो भने २४० मिनेट समय दिनु पर्छ भने यदि नियमीत सेवा समूहमा पर्नु भयो भने १८० मिनेट समय दिनु पर्ने हुन्छ ।

हामीले हरेक पटक तपाईलाई प्रश्नहरु सोध्दा र भर्चुअल परामर्श दिन फोन गर्दा तपाईको मोबाईलमा १०० रुपैया बराबरको पैसा पठाउने छौ ।

तपाई सहभागी भएमा कुनै जोखिमहरु छन्?

यस अध्यनमा भाग लिदा तपाईलाई कुनैपनि हानि पुग्नेछैन तर तपाईले आफ्नो महिनावारी वा गर्भवती सम्बन्धि जानकारी हामीलाई दिन अप्ठ्यारो वा असहज महसुस गर्न सक्नुहुन्छ। यदि तपाईलाई सहभागी हुन मन नलागेमा बिचमा छाड्न पनि सक्नुहुनेछ। यदि तपाईलाई हामीले सोधेका प्रश्नहरुबाट कुनै भावनाहरु उत्पन्न भई कसैसँग कुरा गर्न मन लागेमा कृपया हर्ड ईन्टरनेशनलको कर्मचारीलाई सम्पर्क गर्नुहोस्।

फाईदाहरु

यस अध्ययनमा सहभागि भएमा तपाईलाई प्रत्यक्ष केही फाइदा हुने छैन तर तपाईकै समुदाय तथा नेपालको अन्य भागमा रहनु हुने स्वास्थ्य सेवा बाट बन्चित भएका गर्भवती महिलहरुलाई यो अध्ययनको तथ्यांकले भर्चुअल बिधीबाट सेवा पुर्याउन सकीने रैछ भन्ने आधारहरु हुने छन् र उचित स्वास्थ्य सेवा, स्याहार, पोषणयुक्त खाना सम्बन्धि जानकारी हरु पाउने सक्ने छन ।

तपाईको जानकारी गोप्य राखिनेछ

हर्डको अन्तर्वार्ता लिने व्यक्ति वा स्थानिय महिला स्वास्थ्य स्वयंसेविकालाई दिनुभएका सम्पूर्ण जानकारी गोप्य राखिनेछन्, अर्थात तपाईले उहाँहरुलाई दिनुभएको जानकारी उहाँहरुले अरुलाई भन्ने अनुमति पाउनु भएको छैन।

हाम्रो रेकर्डमा तपाईलाई पहिचान गर्नका लागि युनिक नम्बरको (मेल नखाने परिचयात्मक संख्या) प्रयोग गरिनेछ। फलो अप गर्नुपर्ने सहभागी सूची तयार गर्ने व्यक्ति बाहेक सबैका लागि तपाईको पहिचान गोप्य राख्नका लागि नामको सट्टामा यो नम्बर प्रयोग गरिन्छ। तपाईले दिनुभएका जानकारीहरु पासवर्डले सुरक्षित कम्प्यूटरमा सुरक्षित राखिन्छन् र आधिकार प्राप्त सीमित व्यक्तिलाई मात्र त्यो हेर्न अनुमति दिईनेछ। तपाईले यस मञ्जुरिनामा मा हस्ताक्षर गरेपश्चात, तपाईले मञ्जुरीनामा रद्द नगरेसम्म तपाईको जानकारीहरु यस अध्ययनमा समावेश हुनेछ।

नैतिक स्विकृती

यस अध्ययनलाई नेपाल स्वास्थ्य अनुसन्धान परिषद् (परियोजना आई.डि.नम्बर:...........) र यू.सी.एल अनुसन्धान तथा नैतिक समिति (परियोजना आई.डि नम्बर: ....) र लण्डन स्कुल अफ हाईजिन तथा ट्रपिकल मेडिसिन नैतिक समिती (आई.डी. नम्बर ....) द्वारा स्विकृत गरिएको छ ।

सहभागीताका लागि सहमती

तपाईको सहभागीता नितान्त स्वेच्छिक हुन्छ। यदि तपाईलाई सहभागी हुन मन छैन भने तपाईले कुनै पनि समस्या नमानि अस्वीकार गर्न सक्नुहुन्छ। यदि तपाई सहभागी हुनुभयो र कुनै पनि समयमा तपाई यस अध्ययनबाट हट्न चहानुभएमा कृपया हामीलाई भनिदिनुहोला। हामी तपाईकोमा आउन छाड्नेछौ। यदि तपाईले सहभागी हुने निर्णय लिएमा तपाईलाई यो जानकारी पत्र राख्न दिई लिखित मञ्जुरीनामा दिन अनुरोध गरिनेछ।

यस अध्ययनमा भाग लिने वा नलिने भन्ने कुराको निर्णय तपाई आफैले गर्ने हो। तपाई भाग लिन पनि सक्नु हुन्छ, नलिन पनि सक्नु हुन्छ र लिएर बिचमा छोड्न पनि सक्नु हुन्छ। यदि बिचमा छोड्न चाहनु हुन्छ भने तपाईले छोडे पछि तपाईको नाम अध्ययन बाट हटाईनेछ। तपाईले भाग नलिदा वा बिचमा छोड्दा यस अध्ययनका कर्मचारीहरु संग तपाईको सम्बन्धमा केहि फरक पर्ने छैन, तपाईलाई कुनै जरिवाना लाग्दैन, र तपाईले स्वास्थ्य संस्था बाट पाउने अन्य सुविधाहरुमा कुनै कमि आउने छैन।

तथ्याङ्क संकलन गर्ने तरिका

अध्ययनको सबै सहभागीहरुसंग दुई पटक तथ्याङ्क संकलन गरिनेछ - पहिलो पल्ट गर्भवती भएको १४ – २५ हप्तामा र दोश्रो पटक ३२- ३६ हप्ताको गर्भावस्थामा । तथ्याङ्क संकलन कोभीड – १९ को संक्रमणको अबस्थालाई हेरेर अवस्था अनुसार फोनबाट वा प्रत्यक्ष भेटेर गरिनेछ। गर्भवती महिलालाई गर्भको उमेर यकीन भएपछि, अध्ययनमा तथ्याङ्क सहायकले गर्भवती महिलासंग अध्ययनमा समावेश हुनका लागि फोनबाट मन्जुरिनामा लिनुहुनेछ। यस अध्ययनका सबै तथ्यांकहरु गोप्य राखिनुका साथै हर्डको कार्यालयमा सुरक्षित ढङ्गले सर्भर र कम्पयुटरहरुमा स्टोर गरिनेछ जसमा आधिकारिक व्यक्तिको मात्र पहुँच हुनेछ। सहभागीहरुलाई यस अध्ययनमा समावेश गर्नु अगावै लिखित मञ्जुरिनामा लिईनेछ।

अनुसन्धानको नतिजाहरुको अपेक्षित परिणाम

यस अध्ययन अन्तर्गत गर्भवती महिलालाई ट्याबलेट मार्फत भर्चुअल तरिका प्रयोग गरेर गरिएको पोषण परामर्शले गर्भवती महिलालले गर्भावस्थामा चाहिने आइरन र् फोलिक ऐसिडको नियमित सेवन, आइरन युक्त खाने कुराहरु खान र गर्भ जाँचमा के कस्तो सुधार ल्याउन सकिन्छ भन्ने कुरा बुझ्न मद्दत गर्नेछ। पोषण सहायकले भर्चुअल परामर्शका समयमा गर्भ जाँच र संस्थागत सुत्केरी गराउनमा प्रोत्साहन गर्ने भएकाले गर्भ जाँच र संस्थागत सुत्केरी बढ्छ भन्ने आशा राखेका छौं। यस अध्ययनको परिणामहरुमा आएको संयुक्त सुधारको प्रभावले रक्तअल्पता घटाउनुका साथै गर्भवती महिलाको स्वास्थ्यमा सुधार ल्याउनेछ।

अनुसन्धानको नतिजाहरुको उपयोगको योजना

यस अनुसन्धानका नतिजाहरु मातृ पोषण र गर्भावस्थामा हुने रक्तअल्पता सम्बन्धि कार्यक्रमहरुको भावी योजना बनाउदा प्रमाणमा आधारित निर्णय लिन सम्बन्धित सरोकारवालाहरुलाई सूचित गराउनका लागि प्रयोग हुनेछन्।

अन्य जानकारी

यस अध्ययनबारे अन्य व्यक्तिसँग छलफल गर्नुपरेमा वा हामीलाई कुनै प्रश्नहरु सोध्नुपरेमा निर्धक्कसाथ सोध्न सक्नुहुन्छ।

यदि तपाईका अन्य प्रश्नहरु वा जिज्ञासा भएमा, निम्न व्यक्ति वा संस्थालाई सम्पर्क गर्नुहोला: ट्रायल म्यानेजर, हर्ड इन्टरनेशनल, प्रसुतिगृह मार्ग, थापाथली, काठमाण्डौ. सम्पर्क नं ०१-४२३८०४५

वा

डा. नावमी सेभिल, सिनियर रिसर्च असोसिएट, युनिभर्सिटी कलेज लन्डन ईन्स्टिच्यूट फर ग्लोबल हेल्थ र प्राविधिक सल्लाहाकार; हर्ड, काठमाण्डौ, नेपाल। सम्पर्क नं ०१-४२३८०४५

वा

हर्ड ईन्टरनेशनल जिल्ला कार्यालय, तौलीहवा, कपिलबस्तु,सम्पर्क नं ०७६-५९००९०

For peer review only

Awadhi version of the Participnt information sheet

परियोजना परिचय: गर्भावस्थामे आईरन-फोलिक एसिड कै सेवनमे सुधार कै खातिर भर्चुअल परामर्श कार्य कै अध्ययन (VALID)

काडमाण्डौमे रहेवाला हर्ड ईन्टरनेशनल राष्ट्रीय स्तर कै अनुसन्धान करेवाला संस्था होय। यी संस्था स्वास्थ्य, वातावरण अउर सामाजिक क्षेत्रमे उल्लेख्य कार्यक्रम औ अनुसन्धान करत आवत है। हर्ड ईन्टरनेशनल गर्भवती महतारीमे आईरन-फोलिक एसिड सेवन कै अवस्थामे सुधार कै खातिर लुम्बिनी प्रदेश कै कपिलवस्तु जिलामे "गर्भावस्थामा भर्चुअल परामर्श कार्यको अध्ययन" नाव कै अनुसन्धान करेवाला जानकारी अनुरोध काराजात है। यी अनुसन्धान सञ्चालन कै खातिर यी संस्था नेपाल स्वास्थ्य अनुसन्धान परिषद्से अनुमति पायी चुकाहै अउर स्वास्थ्य सेवा विभाग अन्तर्गत कै परिवार कल्याण महाशाखाके अवगत कराय चुकागयहै। यी अध्ययन शुरु करे से पहिले हमरे प्रदेश अउर स्थानिय सरकारसे समन्वय कईकय आवश्यक सहयोग भी लेवाजाइ।

पृष्ठभूमि

रक्तअल्पता वा खून कै कमीके मतलब खून मै लाल कोशिका कै मात्रा सामान्य अवस्थासे कम होयक वा हरेक लाल कोशिकामे सामान्यसे कम हेमोग्लोबिन होयक अवस्था होय। आईरन कै कमी ही रक्तअल्पता होयक सामान्य अउर प्रमुख कारण होय। गर्भवती अवस्था मै महतारी कय मृत्यु कय कारण मध्ये २० प्रतिशत कारण रक्तअल्पतासे सिधै सम्बन्धित होतहय। गम्भिर रक्तअल्पता कै कारणसे विश्वमे होयवाला आधासे ज्यादा महतारी कै मृत्यु दक्षिण एसिया मै होतहय। आईरन कै वजहसे गर्भावस्थामे जटिलता अउर अस्वस्थता होयक वजहसे गर्भवती अवस्थामे होयवाला रक्तअल्पता घटायक बहुत ही जरुरी है। महतारी सौरी होत कै समयमे रक्तअल्पता नहोअल गर्भवती महतारी से ज्यादा रक्तअल्पता होअल गर्भवती महतारी कै मृत्यु होयक सम्भावना ज्यादा देखात है अउर वनसे कम तौल कै शिशु कै जन्म होला।

नेपाल कै सन्दर्भमे गर्भवती महतारीमे होयवाला रक्तअल्पता अन्य जगहसे तराईमे ज्यादा (३६.४%) देखातहय। सब प्रदेशमे से उच्च रक्तअल्पता होयवालामे लुम्बिनी प्रदेश दुसरा (४३.५%) स्थानमे परत है। नेपालमे नियमित गर्भजाच (कमसे कम ४ दाँयी) अउर पाउभारी होअल १३ हप्तासे नियमित आईरन अउर फोलिका एसिड (१८० गोली) वितरण करेवाला सरकारी नियम है लेकिन सरकारी नियम अनुसार नियमित गर्भजाच करेवाला संख्या बहुत कम है अउर वितरण होयवाला आईरन फोलिक एसिड गोलीमे पहुच भी बहुत कम है। जेकरे वजहसे गर्भावस्था मै होयवाला रक्तअल्पता बहुत ज्यादा देखातहय। वर्तमान कोभिड-१९ कै महामारी कै समय मै स्वास्थ्य संस्था जायक लिए मनईनमे रहल डर, महामारी नियन्त्रण कै खातिर बार बार किया गवल बिभिन्न स्वरुप कै निषेधाज्ञा कै वजहसे स्वास्थ्य सेवामे मनईन कै पहुच सिमित भयक वजहसे भी महतारी अउर शिशु कै पोषण स्थिति मै ह्रास आवल है।

उद्देश्य

यी अध्ययन कै उद्देश्य गर्भावस्था मै आईरन-फोलिक एसिड कै सेवन नियमित करवाइ कै आईरन कै कमी से होयवाला रक्तअल्पता घटाई कै गर्भवती महतारी कै पोषणमे सुधार करेक साथै उपलब्ध स्वास्थ्य सेवा कै उपयोग औ पहुच बढायक होय।

विधि

यी अध्ययन कपिलवस्तु जिला कै ९ ठु पालिकामे रहेवाला १३ से लईकय ४९ वरष तक कै शादिशुदा गर्भवती महतारीमे संचालन कराजाई। हमरे यी अध्ययन मै ३०० जने गर्भवती महतारी कै समावेश करेक योजना है, जे का दुई ठु समुह मै बाँटा जाइ:

३. पहिला समुहमे १५० जने महिला कै यी अध्ययन कै अवधिके लिए सिमकार्ड सहित कै ट्याबलेट (बडका स्क्रिनवाला मोबाईल) देवा जाइ अउर हम्मन कै पोषण सहायक उ ट्याबलेट कै माध्यमसे वन्हनकै रक्तअल्पता, बढिया खानपान कै व्यवहार अउर गर्भ जाँचसे सम्बन्धित शिक्षा, परामर्श औ सल्लाह देवेकै साथै खानपान सम्बन्धि सल्लाह दिहै। साथै गर्भवती महतारी कै नजिक कै स्वास्थ्य संस्थामे जाई कै गर्भवती जाँच करेक लिय प्रोत्साहित करिहै। वन्हरे गर्भावस्थामे होयवाला रक्तअल्पताके समस्या कै साथै खानपानमे सुधार कईकै, आईरन गोली सेवन कईकै अउर गर्भ जाँच कईकै कैसे रक्तअल्पता घटाइ सकाजातहै वकरे बारेमे छलफल करीहै।

४. दुसर समूह कै १५० जने महिला कै नेपाल सरकार कै स्वास्थ्य कार्यक्रमद्वारा प्रदान होअल स्वास्थ्य सेवा सहित नियमित गर्भ जाँच अउर आईरन गोली प्राप्त करिहै।

हमरे के का सहभागी होयक लिय आमन्त्रण कराजातहै?

हमरे १२ से २८ हफ्ता बीच कै गर्भवती महतारी लोगन कै मात्र यी अध्ययनमे सहभागी करावाजाइ । यी अध्ययन कै लिए आवश्यक परेवाला गर्भवती महतारी हमरे स्वास्थ्य संस्था अउर स्वयंसेविक कै सहयोगसे समावेस करावाजाइ । आप यी अध्ययन मै सहभागी होयक योग्य होअल नहोअल एकिन आप कै स्थानिय महिला स्वयंसेविका, स्वास्थ्यकर्मीके साथ मै आयवाला हर्ड कै तथ्याङ्क सहायक करिहै । आप योग्य होअल नहोअल एकिन करेक अउर अध्ययनमे सहभागी होयक नहोयक निर्णय लेक लिए हमरे आप कै यी ट्रायल कै बारेमे पत्र पढे कै देवाजाइ साथमे यह मै दिहल जानकारी सब समझेक बुझेक बादमे आप कै मंजुरीनामामे हस्ताक्षर करे कै पडी । यी अध्ययन मै सहभागी होयसे होयवाला जोखिम वा खतरा एकदम कम है । यी अध्ययनमे आप कै सहभागीता पूर्ण रुपसे आपके विचार उपर है अउर अगर कौनो कारणसे बीचमे छोडेक चाहाजात है तो छोडक पावाजाइ ।

अगर आप यी अध्ययनमे सहभागी होयक लिए सहमत होवाजाइ तो का होइ ?

For peer review only

अगर आप यी ट्रायलमे सहभागी होयक मञ्जुरीनामा दियाजाइ तो हमरे आप कै कुछ व्यक्तिगत जईसे परिवार, कमाई, प्रजनन स्वास्थ्य आदि अउर आईरन-फोलिक एसिड सेवन कै बारेमे प्रश्न पुछाजाइ। यी कुल प्रश्न पुछे कै बादमे हरेक सहभागी महिला लोगन कै गोला प्रथासे अध्ययन कै दुई समूह मे से कौनो एक समूह मै समावेश कराजाइ। यी कूल प्रक्रिया, प्रश्न पुछेवाला काम अउर समुह बिभाजन करेक लिए लगभग ९० मिनेट समय लागी। आप जौन कौनो समूहमे परेपर भी हमरे फिर एक पटक प्रश्न पुछेक लिए लगभग एक महिनामे भेट करेक लिए आवाजाई (फोन वा प्रत्यक्ष)। उ समयमे हमरे आप कै गर्भवती जाँच, खानपान कै बारेमे, आईरन अउर फोलिक एसिड, परिवार कै सहयोग अउर अध्ययनमे सहभागी होत कै अनुभवके बारेमे प्रश्न पुछाजाइ। यकरे लिए आप लगभग ९० मिनेट जतना समय दियक पडी।

लेकिन अगर आप भर्चुअल परामर्श समूहमे पराजाइ तो हमरे आप कै यी अध्ययन अवधिभर कै लिए सिमकार्ड सहित कै ट्याबलेट देवाजाइ। वकरे बादमे हमरे आप कै दुई दाँयी भर्चुअल परामर्शके लिए उ ट्याबलेटमे फोन कराजाइ अउर हरेक दाँयी लगभग ९० से १२० मिनेट समय देकय पडी। भर्चुअल परामर्श सत्र मै आप अउर आप कै परिवार कै अन्य सदस्य संघरी गर्भावस्थामे आईरन युक्त खान कै महत्व अउर आईरन युक्त खाना खायम होअल समस्या सम्बन्धि छलफल करेक साथै आवश्यक्ता अनुसार कै परामर्श देवाजाइ अउर छलफलके आधार मै समस्या समाधान करेक खातिर कार्ययोजना बनावाजाइ।

यी अध्ययनमे सहभागी होय पर अगर भर्चुअल परामर्श समूहमे परागय तो लगभग ४२० मिनेट समय देयक पडी अउर अगर नियमित सेवा समुहमे परा गय तो १८० मिनेट जतना समय देयक पडी।

हमरे हरेक बार आपसे प्रश्न पुछतके अउर भर्चुअल परामर्श देत कै फोन कराजाइ तो आप कै मोबाईल मै १०० रुपैया बराबर पैसा पठावा जाइ।

आपके सहभागी होये पर कौनो जोखिम है ?

यी अध्ययन मै भाग लिहेसे आप कै कौनो भी हानी पहुचेवाला नाहि है लेकिन आपन गर्भावस्था कै जानकारी हम्मन कै देयक परेशानी औ असहज महसुस होय सकत है। अगर आप कै सहभागी होयक मन नाहि रहि तो बिचमे छोड भी सकाजात है। अगर आप कै हम्मनके पुछल कौनो प्रश्नसे कौनो भावना पैदा होय कै केहुसे बात करेक मन लागेपर कृपया हर्ड कै कर्मचारीसे सम्पर्क कराजाइ।

फाईदा

यी अध्ययन मै सहभागी होय पर आप कै प्रत्यक्ष कुछु फाइदा नाहि होयी लेकिन आप कै समुदाय अउर नेपाल कै अउर जगहीमे रहल स्वास्थ्य सेवासे बन्चित भवल गर्भवती महतारीयन कै यी अध्ययन कै तथ्याङ्कसे भर्चुअल तरिकासे सेवा पहुचायक सकाजात है कहेवाला आधार होयी अउर उचित स्वास्थ्य सेवा, स्याहार, पोषण रहल खाना कै बारेमे जानकारी पायसकत है।

आप कै जानकारी गोप्य रख्खाजाइ

हर्डसे अन्तरवार्ता लेवाला ब्यक्ति औ स्थानिय महिला सामुदायिक स्वास्थ्य स्वयंसेविका कै देवल सब जानकारी गोप्य रख्खाजाइ, मतलब आप वन कै देवल जानकारी वन्नहरे औरो केहुसे कहेक अनुमति नाहि मिला है। हम्मन कै रेकर्डमे आप कै चिन्हेक लिये युनिक नम्बर (दुसरे से नमिलेवाला परिचयात्मक संख्या) कै प्रयोग कराजाइ। बादमे भेट (फलो अप) करेवाला सहभागी सूची तयार करेवाला व्यक्तिके सिवाय सबके लिये आप कै पहिचान गोप्य रख्खे कै लिये नाव कै औजि पर यी नम्बर प्रयोग कियाजाइ। आप कै देवल जानकारी पासवर्डसे सुरक्षित करल कम्प्युटर मै सहेज कै रखिजाइ अउर अधिकार पावल कुछ सीमित व्यक्ति कै भर यी देखे कै अनुमति देवाजाइ। आप यी मञ्जुरीनामामे हस्ताक्षर करेक बादमे, आप यइ मञ्जुरीनामा रद्द नकरे तक आप कै जानकारी यी अध्ययन मै समावेश होयी।

नैतिक स्विकृती

यी अध्ययन कै नेपाल स्वास्थ्य अनुसन्धान परिषद् (परियोजना आई.डि.नम्बर: ५७०।२०२१) से स्विकृत भवा है।

सहभागीता कै लिये सहमती

आप कै सहभागीता पुरापुर आप कै बिचार उपर रहि। अगर आप कै सहभागी होयक मन नाहि है तो कौनो भी दिक्कत परेसानी नमान कै अस्वीकार कै सका जात है। अगर आप यी अध्ययनमे सहभागी होवागै अउर कौनो भी समयमे आप यी अध्ययनसे हटेक चाहाजात है तो हम्मन से कहाँ जाइ। हमरे आपके यहा नाहि आवाजाइ। अगर आप सहभागी होयेक निर्णय लेवाजाइ तो यी जानकारी पत्र आप कै रख्खे देवाजाइ अउर लिखित मञ्जुरीनामा देयकै अनुरोध कराजाइ। यी अध्ययन मै भाग लियेक औ नलियेक निर्णय आप कै अपने करेक है। आप सहभागी होय सकाजात है, न भी होयसकाजात है अउर बिचमे भी छोड सकाजात है। अगर बिचमे छोडे कै चाँहा जात है तो आप कै छोडे कै बादमे आप कै नाव अध्ययनसे हटाइजाइ। आप कै सहभागी नहोयेसे अउर बिच मै छोडेसे यी अध्ययन कै कर्मचारीयनसे आप कै सम्बन्ध मै कुछ फरक परेवाला नाहि है, आप कै कौनो जरिवाना भी नाहि लागी अउर आप कै स्वास्थ्य संस्थासे पायेवाला और कौनो सुविधा मै कुछ भी कमी नाहि आयेवाला है।

तथ्याङ्क संकलन करेवाला तरिका

अध्ययन कै सब सहभागीयन से दुई दाँयी तथ्याङ्क संकलन कराजाइ – पहिला दाँयी गर्भवती होअल १२ – २८ हफ्तामे अउर दुसरा दाँयी १६ – ३२ हफ्ताके गर्भावस्थामे। तथ्याङ्क संकलन कोभीड – १९ कै संक्रमण कै अवस्था देख कै फोनसे वा प्रत्यक्ष भेटसे कराजाइ। गर्भवती महतारीके गर्भ कै महिना पता चलेक बादमे, अध्ययनमे तथ्याङ्क सहायक गर्भवती महतारीसे अध्ययनमे सहभागी होयेक लिये फोनसे मञ्जुरीनामा लिहैं। यी अध्ययन

For peer review only

कै सब तथ्याङ्क गोप्य रख्खे कै साथै हर्ड कै कार्यालयमे सुरक्षित रुपसे सर्भर अउर कम्प्युटरमे रख्खाजाइ जेह्मा आधिकारिक व्यक्ति कै भर पहुच रही। सहभागीयन कै यी अध्ययनमे सहभागी होयेसे पहिले ही लिखित मञ्जुरिनामा लिहाजाइ।

अनुसन्धान नतिजा कै अपेक्षित परिणाम

यी अध्ययनमे गर्भवती महतारी कै ट्याबलेटसे भर्चुअल तरिका प्रयोग कइ कै करल पोषण परामर्शसे गर्भवती महतारी कै गर्भावस्थामे चाहेवाला आईरन औ फोलिक एसिडके नियमित सेवन, आईरन होअल खाना खायेक अउर गर्भ जाँचमे कैसन सुधार कै सकाजात है कहेवाला बतिया बुझेक मद्त होइ। पोषण सहायकसे भर्चुअल परामर्शके समयमे गर्भ जाँच अउर संस्थागत सौरी करवायेक प्रोत्साहन करेक वजहसे गर्भ जाँच अउर संस्थागत सौरी बढी कहि कै आशा रख्खा गै है। यी अध्ययन कै परिणाम मै आवल संयुक्त सुधार कै प्रभावसे रक्तअल्पता घटावै कै साथै गर्भवती महतारी कै स्वास्थ्य मै सुधार लायी।

अनुसन्धान कै नतिजा कै उपयोग कै योजना

यी अनुसन्धानसे आवल नतिजा मातृ पोषण अउर गर्भावस्थामे होयेवाला रक्तअल्पता जैसन कार्यक्रम कै भावी योजना बनावत कै प्रमाणके आधारमे निर्णय लेकलिये सम्बन्धित सरोकारवालन कै सूचित करायेक खातिर प्रयोग कयिजाइ।

और जानकारी

यी अध्ययन बारेमे और व्यक्ति संघरी छलफल करेक वा हम्मन कै कौनो प्रश्न पुछेक है तो बिना हिचकिचाहट पुछसकाजात है।

अगर आप कै अउर प्रश्न वा जिज्ञासा है तौ, निचे दिहल व्यक्ति वा संस्था कै सम्पर्क कियाजाइ:

ट्रायल म्यानेजर, हर्ड इन्टरनेशनल, प्रसुतिगृह मार्ग, थापाथली, काठमाण्डौ. सम्पर्क नं ०१-४२३८०४५

वा

डा. नावमी सेभिल, सिनियर रिसर्च असोसिएट, युनिभर्सिटी कलेज लन्डन ईन्स्टिच्यूट फर ग्लोबल हेल्थ औ प्राविधिक सल्लाहाकार; हर्ड, काठमाण्डौ, नेपाल। सम्पर्क नं ०१-४२३८०४५

वा

हर्ड ईन्टरनेशनल जिल्ला कार्यालय, तौलीहवा, कपिलबस्तु, सम्पर्क नं ०७६-५९००९०

For peer review only

STANDARD PROTOCOL ITEMS: RECOMMENDATIONS FOR INTERVENTIONAL TRIALS

SPIRIT 2013 Checklist: Recommended items to address in a clinical trial protocol and related documents*

Section/item	Item No	Description	Addressed on page number
**Administrative information**			
Title	1	Descriptive title identifying the study design, population, interventions, and, if applicable, trial acronym	1
Trial registration	2a	Trial identifier and registry name. If not yet registered, name of intended registry	1
	2b	All items from the World Health Organization Trial Registration Data Set	1
Protocol version	3	Date and version identifier	1
Funding	4	Sources and types of financial, material, and other support	1
Roles and responsibilities	5a	Names, affiliations, and roles of protocol contributors	1
	5b	Name and contact information for the trial sponsor	2
	5c	Role of study sponsor and funders, if any, in study design; collection, management, analysis, and interpretation of data; writing of the report; and the decision to submit the report for publication, including whether they will have ultimate authority over any of these activities	2
	5d	Composition, roles, and responsibilities of the coordinating centre, steering committee, endpoint adjudication committee, data management team, and other individuals or groups overseeing the trial, if applicable (see Item 21a for data monitoring committee)	2

For peer review only

**Introduction**			
Background and rationale	6a	Description of research question and justification for undertaking the trial, including summary of relevant studies (published and unpublished) examining benefits and harms for each intervention	5
	6b	Explanation for choice of comparators	7
Objectives	7	Specific objectives or hypotheses	6
Trial design	8	Description of trial design including type of trial (eg, parallel group, crossover, factorial, single group), allocation ratio, and framework (eg, superiority, equivalence, noninferiority, exploratory)	7
**Methods: Participants, interventions, and outcomes**			
Study setting	9	Description of study settings (eg, community clinic, academic hospital) and list of countries where data will be collected. Reference to where list of study sites can be obtained	7
Eligibility criteria	10	Inclusion and exclusion criteria for participants. If applicable, eligibility criteria for study centres and individuals who will perform the interventions (eg, surgeons, psychotherapists)	7
Interventions	11a	Interventions for each group with sufficient detail to allow replication, including how and when they will be administered	8
	11b	Criteria for discontinuing or modifying allocated interventions for a given trial participant (eg, drug dose change in response to harms, participant request, or improving/worsening disease)	14
	11c	Strategies to improve adherence to intervention protocols, and any procedures for monitoring adherence (eg, drug tablet return, laboratory tests)	9
	11d	Relevant concomitant care and interventions that are permitted or prohibited during the trial	9
Outcomes	12	Primary, secondary, and other outcomes, including the specific measurement variable (eg, systolic blood pressure), analysis metric (eg, change from baseline, final value, time to event), method of aggregation (eg, median, proportion), and time point for each outcome. Explanation of the clinical relevance of chosen efficacy and harm outcomes is strongly recommended	9 Tables 1 & 2
Participant timeline	13	Time schedule of enrolment, interventions (including any run-ins and washouts), assessments, and visits for participants. A schematic diagram is highly recommended (see Figure)	17

For peer review only - http://bmjopen.bmj.com/site/about/guidelines.xhtml

Sample size	14	Estimated number of participants needed to achieve study objectives and how it was determined, including clinical and statistical assumptions supporting any sample size calculations	11
Recruitment	15	Strategies for achieving adequate participant enrolment to reach target sample size	11-12
**Methods: Assignment of interventions (for controlled trials)**			
Allocation:			
Sequence generation	16a	Method of generating the allocation sequence (eg, computer-generated random numbers), and list of any factors for stratification. To reduce predictability of a random sequence, details of any planned restriction (eg, blocking) should be provided in a separate document that is unavailable to those who enrol participants or assign interventions	8
Allocation concealment mechanism	16b	Mechanism of implementing the allocation sequence (eg, central telephone; sequentially numbered, opaque, sealed envelopes), describing any steps to conceal the sequence until interventions are assigned	8
Implementation	16c	Who will generate the allocation sequence, who will enrol participants, and who will assign participants to interventions	8
Blinding (masking)	17a	Who will be blinded after assignment to interventions (eg, trial participants, care providers, outcome assessors, data analysts), and how	8
	17b	If blinded, circumstances under which unblinding is permissible, and procedure for revealing a participant's allocated intervention during the trial	8
**Methods: Data collection, management, and analysis**			
Data collection methods	18a	Plans for assessment and collection of outcome, baseline, and other trial data, including any related processes to promote data quality (eg, duplicate measurements, training of assessors) and a description of study instruments (eg, questionnaires, laboratory tests) along with their reliability and validity, if known. Reference to where data collection forms can be found, if not in the protocol	11
	18b	Plans to promote participant retention and complete follow-up, including list of any outcome data to be collected for participants who discontinue or deviate from intervention protocols	13

Data management	19	Plans for data entry, coding, security, and storage, including any related processes to promote data quality (eg, double data entry; range checks for data values). Reference to where details of data management procedures can be found, if not in the protocol	13
Statistical methods	20a	Statistical methods for analysing primary and secondary outcomes. Reference to where other details of the statistical analysis plan can be found, if not in the protocol	12
	20b	Methods for any additional analyses (eg, subgroup and adjusted analyses)	12
	20c	Definition of analysis population relating to protocol non-adherence (eg, as randomised analysis), and any statistical methods to handle missing data (eg, multiple imputation)	13
**Methods: Monitoring**			
Data monitoring	21a	Composition of data monitoring committee (DMC); summary of its role and reporting structure; statement of whether it is independent from the sponsor and competing interests; and reference to where further details about its charter can be found, if not in the protocol. Alternatively, an explanation of why a DMC is not needed	2, 12
	21b	Description of any interim analyses and stopping guidelines, including who will have access to these interim results and make the final decision to terminate the trial	16
Harms	22	Plans for collecting, assessing, reporting, and managing solicited and spontaneously reported adverse events and other unintended effects of trial interventions or trial conduct	16
Auditing	23	Frequency and procedures for auditing trial conduct, if any, and whether the process will be independent from investigators and the sponsor	16
**Ethics and dissemination**			
Research ethics approval	24	Plans for seeking research ethics committee/institutional review board (REC/IRB) approval	16
Protocol amendments	25	Plans for communicating important protocol modifications (eg, changes to eligibility criteria, outcomes, analyses) to relevant parties (eg, investigators, REC/IRBs, trial participants, trial registries, journals, regulators)	17

Consent or assent	26a	Who will obtain informed consent or assent from potential trial participants or authorised surrogates, and how (see Item 32)	8, 16-17
	26b	Additional consent provisions for collection and use of participant data and biological specimens in ancillary studies, if applicable	Not applicable
Confidentiality	27	How personal information about potential and enrolled participants will be collected, shared, and maintained in order to protect confidentiality before, during, and after the trial	13, 17
Declaration of interests	28	Financial and other competing interests for principal investigators for the overall trial and each study site	2
Access to data	29	Statement of who will have access to the final trial dataset, and disclosure of contractual agreements that limit such access for investigators	13
Ancillary and post-trial care	30	Provisions, if any, for ancillary and post-trial care, and for compensation to those who suffer harm from trial participation	Not applicable
Dissemination policy	31a	Plans for investigators and sponsor to communicate trial results to participants, healthcare professionals, the public, and other relevant groups (eg, via publication, reporting in results databases, or other data sharing arrangements), including any publication restrictions	17
	31b	Authorship eligibility guidelines and any intended use of professional writers	18
	31c	Plans, if any, for granting public access to the full protocol, participant-level dataset, and statistical code	17
**Appendices**			
Informed consent materials	32	Model consent form and other related documentation given to participants and authorised surrogates	Supplemental Annex 1
Biological specimens	33	Plans for collection, laboratory evaluation, and storage of biological specimens for genetic or molecular analysis in the current trial and for future use in ancillary studies, if applicable	Not Applicable

*It is strongly recommended that this checklist be read in conjunction with the SPIRIT 2013 Explanation & Elaboration for important clarification on the items. Amendments to the protocol should be tracked and dated. The SPIRIT checklist is copyrighted by the SPIRIT Group under the Creative Commons "Attribution-NonCommercial-NoDerivs 3.0 Unported" license.

For peer review only - http://bmjopen.bmj.com/site/about/guidelines.xhtml
